# Exploring *ortho*-dianthrylbenzenes for molecular solar thermal energy storage†

**DOI:** 10.1039/d4ta03879g

**Published:** 2024-08-15

**Authors:** Nicolò Baggi, Lidiya M. Muhammad, Zacharias Liasi, Jacob Lynge Elholm, Paulius Baronas, Elies Molins, Kurt V. Mikkelsen, Kasper Moth-Poulsen

**Affiliations:** a The Institute of Materials Science of Barcelona, ICMAB-CSIC Bellaterra 08193 Barcelona Spain kasper.moth-poulsen@upc.edu; b Department of Chemical Engineering, Universitat Politècnica de Catalunya EEBE EduardMaristany 10-14 08019 Barcelona Spain; c Department of Chemistry and Chemical Engineering, Chalmers University of Technology SE-41296 Gothenburg Sweden; d Department of Chemistry, University of Copenhagen Universitetsparken 5 Copenhagen Ø 2100 Denmark; e Catalan Institution for Research & Advanced Studies, ICREA Pg. Lluís Companys 23 08010 Barcelona Spain

## Abstract

Molecular solar thermal systems, which absorb light, store it, and release it as heat, have been extensively researched, yet many potential candidates remain unexplored. To expand this range, five specifically designed *ortho*-dianthrylbenzenes were investigated. Anthracene dimers have been underexplored due to issues like photooxidation and varying photodimerization efficiency. The presented systems address these challenges by aryl-linking two anthracene moieties, achieving photodimerization quantum yields ranging from 11.5% to 16% in mesitylene. The impact of donor or acceptor groups on energy storage time (9–37 years), energy storage density (0.14–0.2 MJ kg^−1^), and solar energy storage efficiency (0.38–0.66%) was evaluated. The experimental results, supported by density functional theory-based modeling, highlight the potential of anthracene-based photoswitches for molecular solar thermal applications and encourage further exploration of similar systems.

## Abstract 2

Aiming to explore anthracene-based systems for molecular solar thermal energy storage, five ortho-dianthrylbenzenes were designed, demonstrating promising properties for future development of anthracene-based photoswitches for such applications.

## Graphical abstract

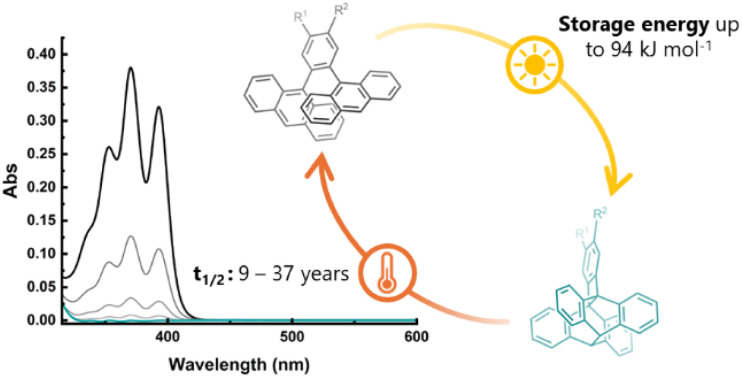

## Introduction

Driven by the need to address the escalating global energy consumption, molecular solar thermal (MOST) systems are emerging as a promising renewable energy storage concept.^1–11^ MOST systems are based on molecular photochromes capable of absorbing sunlight, storing it, and releasing it on-demand as thermal energy in a closed cycle. The four most studied^12^ photoswitches for this application are azobenzenes,^13,14^ norbornadiene–quadricyclane (NBD-QC) couples,^15,16^ dihydroazulenes–vinylheptafulvenes,^17–19^ and (fulvalene)tetracarbonyl-diruthenium derivatives.^20^

Anthracene dimers were first proposed for solar energy storage in 1909,^21^ and the photo-induced dimerization of anthracene has been extensively analyzed.^22–28^ However, while their potential use as solid-state MOST systems has been recently demonstrated by Han and co-workers,^29^ no systematic study has been conducted on their application in solution in solar energy storage, to the best of our knowledge. Despite their promising absorption in the violet portion of the visible spectrum, several factors have limited the exploration of these derivatives for this application. These include the degradation of anthracene monomers upon photooxidation in solution^30^ and their concentration-dependent photodimerization quantum yield.^31^

Generally, both challenges can be mitigated in solid-state switching, as described for multiple anthracene monomers either in single crystals^22,32–34^ or in anthracene-containing polymers.^35–39^ The latter issue can be resolved in solution by linking two anthracene moieties within a single molecule.^40,41^ This linkage can also increase bond strain in the photodimer, thereby enhancing the energy storage density. While most examples in the literature focus on alkyl-linked anthracenes (*e.g.* di(9-anthryl)methane by Bergmark *et al.*),^23^ the use of aromatic linkers has only been recently explored.^42–45^

**Fig. 1:**
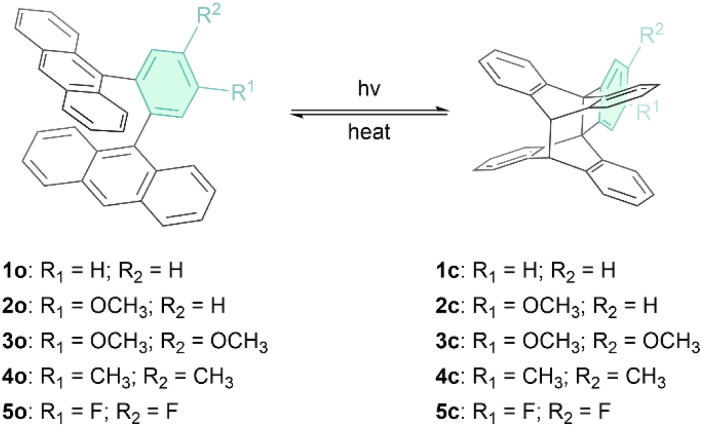
The two states of the five investigated *ortho*-dianthrylbenzenes (1o–5o), where “o” denotes the open form and “c” denotes the corresponding closed photoisomers (1c–5c).

Inspired by the work on 1,2-di(9-anthryl)benzene (1o in Fig. 1) by Nishiuchi, Kubo and co-workers,^42^ who first reported the photoisomerization of this molecule and determined the storage energy (Δ*G*_storage_) and the storage energy density (*ρ*_storage_) in its “closed” photoisomer, 1c (*i.e.* Δ*G*_storage_ = 22.8 kcal mol^−1^ = 95.4 kJ mol^−1^ and *ρ*_storage_ = 0.222 MJ kg^−1^, that is higher than or comparable to the Δ*H* of other reported anthracene systems^23,41,42^ and other MOST candidates^7^), we develop here the series of *ortho*-dianthryl-based photoswitches (1o–5o) depicted in Fig. 1. In these compounds, the phenyl linker is functionalized with electron-donating or electron-withdrawing groups.

Herein, we present a study of the properties of 1o–5o for potential MOST applications, with a focus on the influence of substitutions on the aryl bridge on energy storage time, solar spectrum match and energy storage density. The experimental work is complemented by theoretical modelling to rationalize the experimental observations.

## Results and discussion

Compounds 1o–5o were synthesized *via* Suzuki–Miyaura cross-coupling between 9-anthraceneboronic acid pinacol ester (instead of 9-anthraceneboronic acid, as previously reported in the literature^42,46^) and the corresponding di-halogenated phenyl rings. The detailed experimental procedures and the full characterization of the switches by NMR spectroscopy and high-resolution mass spectrometry (HRMS) are provided in the ESI† (Section “Synthetic procedures”). Among these derivatives, dianthracene 3o, featuring a 4,5-dimethoxy-phenyl linker, had been previously synthesized through Negishi coupling, but no characterization for solar thermal energy storage was reported.^47^ Overall, the *ortho*-dianthrylbenzenes molecules were obtained in yields ranging from 15% to 53%, with higher yields achieved when the product was isolated by recrystallization rather than column chromatography. We speculate that the limited solubility of the molecules may lead to product loss during chromatography, either because of strong adsorption to silica or precipitation from the solution. It is worth noting that 3o was obtained in 68% yield (over two steps) *via* Negishi coupling,^47^ thus indicating that such a synthetic route could be more effective for this class of derivatives.

**Fig. 2:**
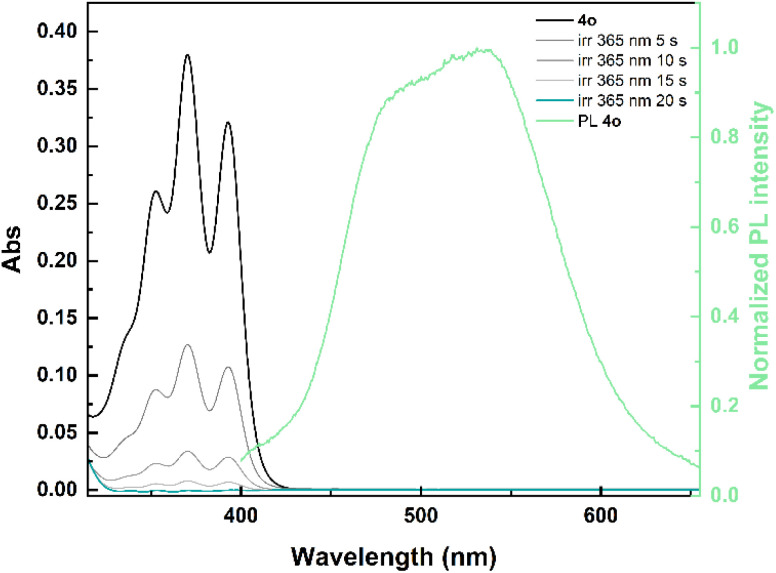
Spectral evolution of 4o (2.2 × 10^−5^ M, black solid line) in mesitylene under light irradiation (365 nm) at room temperature to 4c (blue-green solid line) and normalized emission (clear green solid line) of 4o in mesitylene upon excitation at 365 nm.

The synthesized *ortho*-dianthrylbenzenes were investigated in non-degassed mesitylene at room temperature. The recorded UV-vis spectra were comparable across the series (Fig. S1†), displaying the distinctive bands of the anthracene moiety (Fig. 2). For instance, the absorption of *ortho*-xylene-linked 4o starts at approximately 430 nm and peaks around 370 nm. Spectroscopic data including the maximum absorption wavelength (*λ*_max_), molar absorption coefficient (*ε*) and onset wavelength (*λ*_onset_) are listed in Table 1.

**Table 1:** Main optical data of the investigated photoswitches 1o–5o in mesitylene

	*λ*_max_ (nm) [*ε* (10^3^ M^−1^ cm^−1^)]	*λ*_onset_ a (nm)	*Φ*_o–c_ (%)	*Φ*_F_ (%)
1o	372 [15.6]	425	16	1.8
2o	372 [16.4]	426	13	1.7
3o	373 [17.1]	425	11.5	2.9
4o	372 [17]	424	13	2.2
5o	372 [14.9]	426	16	1.7

a *λ*_onset_: log(*ε*) = 2.

While *λ*_max_ and *λ*_onset_ were unaffected by the introduction of functional groups, the presence of electron-donating groups (*e.g.* –OCH_3_ in 2o and 3o, and –CH_3_ in 4o) resulted in a higher *ε* compared to that of 1o. Conversely, the electron-poorer dianthrylbenzene 5o exhibited the lowest molar extinction coefficient within the series.

Switches 1o–5o are also fluorescent, albeit with low quantum yields (<3%). The emission is observed between approximately 390 nm and 725 nm (emission spectrum of 4o in Fig. 2, emission spectra of 1o–3o and 5o in Fig. S2†). This emission is attributed to a combination of the photoluminescence of anthracene and an intramolecular excimer formation.

Upon irradiation at 365 nm, all photoswitches underwent rapid isomerization (Fig. 2 and S1†) to their corresponding photoisomers 1c–5c, with a concurrent fluorescence quenching as these adducts are non-emissive due to the loss of anthracene conjugation.^42^ Moreover, a large optical contrast can be observed between 1o–5o and 1c–5c since the latter have only negligible absorption between 300 nm and 425 nm.

**Fig. 3:**
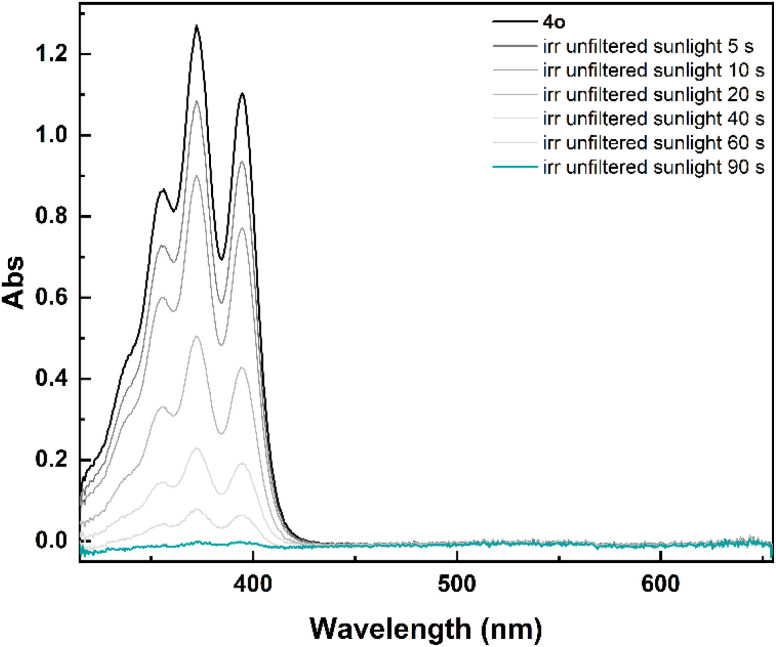
Spectral evolution of 4o (7.5 × 10^−5^ M) to 4c in mesitylene under unfiltered sunlight irradiation.

To assess if the isomerization could be achieved in real solar conditions, a solution of 4o in non-degassed mesitylene was exposed to unfiltered sunlight. The recorded spectra showed also in this case a fast isomerization to 4c (Fig. 3), with no degradation. Considering the comparable optical properties of 1o–3o and 5o, a similar behaviour can be expected also for the other investigated photoswitches.

Both the isomerization (*Φ*o–c) and the fluorescence (*Φ*_F_) quantum yields were determined in mesitylene and are summarized in Table 1.

*Φ*o–c values around 16% were measured for 1o and 5o. In contrast, the three switches bearing donor groups showed lower efficiencies, particularly 3o, with an isomerization quantum yield of 11.5%. These values are comparable to those reported for certain anthracene monomers – where concentration is also a critical factor – and other linked systems.^23,25,26^

Additionally, photo-induced isomerization and fluorescence are two competitive excited-state processes. The fluorescence quantum yield of 3o is the highest within the series (2.9%), while *Φ*_F_ of the other studied molecules range from 1.7 to 1.8% for 2o, 5o and 1o, and is equal to 2.2% for 4o. In general, these values appear lower than those of anthracene or other monomers, likely due to the increased strain in the aryl-linked systems. Details on the determination of *Φ*o–c and *Φ*_F_ are provided in the ESI† (Sections “Determination of the photoisomerization quantum yields” and “Determination of the fluorescence quantum yields” of the ESI†, respectively).

**Fig. 4:**
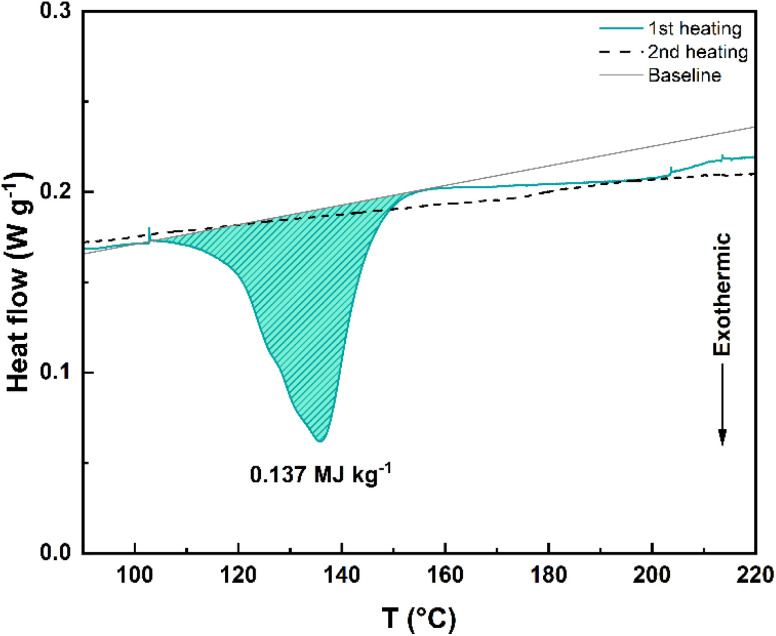
DSC graph of 4c showing the heat release during the first heating cycle (blue-green solid line). No heat release was observed during the second heating cycle (black dashed line). The baseline used for the integration to determine the energy release is provided with a solid grey line for clarity.

Next, the thermal stability of 1c–5c, photogenerated in mesitylene, was studied through differential scanning calorimetry (DSC). The DSC graph of 4c is depicted in Fig. 4 and it shows the exothermic process (only during the first heating cycle, starting at around 100 °C and ending around 155 °C) associated to the putative^42^ stepwise bond dissociation that accompanies the back-reaction. The corresponding storage energy (Δ*G*_storage_) and storage energy density (*ρ*_storage_) are 62.8 kJ mol^−1^ and 0.137 MJ kg^−1^, respectively (Table 2). In the case of 5c, the heat release occurs between 140 °C and 185 °C and an endothermic process (possibly 5c melting) is observed between 80 °C and 100 °C *ca.* (Fig. S3d†).

**Table 2:** Thermodynamic data for thermal back-conversion (1c–5c → 1o–5o) determined from the Eyring equation including the activation enthalpy (ΔH*) and entropy (ΔS*), and half-lives at 25 °C (*t*_1/2_); storage energies (Δ*G*_storage_), storage energy densities (*ρ*_storage_) and solar energy storage efficiencies (*η*_MOST_). The theoretical values, computed at the ωB97X-D3(BJ)/cc-pVTZ level of theory, are given in square brackets for vacuum and *implicit mesitylene*, respectively

	ΔH* (kJ mol^−1^)	ΔS* (J K^−1^ mol^−1^)	*t*_1/2_ (years)	Δ*G*_storage_ (kJ mol^−1^)	*ρ*_storage_ (MJ kg^−1^)	*η*_MOST_ (%)
1	121	−7.6	15	86.1^b^ [63.4, *62.4*]	0.200 [0.147, *0.145*]	0.66
2	127	8.8	25	94.0^b^ [66.9, *63.7*]	0.204 [0.145, *0.138*]	0.58
3	125	3.8	18	69.2^b^ [68.7, *67.7*]	0.141 [0.140, *0.138*]	0.38
4	122	−0.9	9	62.8^a^	0.137^a^	0.41
				65.6^b^ [67.7, *66.0*]	0.143^b^ [0.148, *0.144*]	
5	128	9.0	37	64.8^a^	0.139^a^	0.57
				74.6^b^ [64.3, 63.5]	0.160^b^ [0.138, *0.136*]	

a Prepared in mesitylene.

b Prepared in DMSO.

However, the DSC experiments for 1c–3c (Fig. S3a–c†) showed exothermic events that were difficult to interpret, perhaps due to interactions of the photoisomers with residual traces of mesitylene. Therefore, the dissociation of the C9–C9′ and the C10–C10′ single bonds in 1c–5c to restore the parent molecules 1o–5o was also studied on photoisomers prepared upon irradiation in DMSO (Fig. S4†). In this case, the obtained DSCs allowed the determination of the corresponding storage energies and storage energy densities (Table 2).

For comparison, the observed Δ*G*_storage_ for 1c, 2c and 3c (86.1 kJ mol^−1^, 94.0 kJ mol^−1^ and 69.2 kJ mol^−1^, respectively) are higher than the Δ*G*_storage_ of anthracene (65.2 kJ mol^−1^)^41^ and other linked anthracene-based MOST candidates (*e.g.* Δ*G*_storage_ = 35.5 kJ mol^−1^ in the photoisomer of bi(anthracene-9,10-dimethylene)).^41^

The storage energy densities (*ρ*_storage_) ranging from 0.14 MJ kg^−1^ to 0.2 MJ kg^−1^ within the series appear to be similar to those of some azobenzene-based molecular solar thermal systems or fulvalene-tetracarbonyl-diruthenium, but lower than that of norbornadiene–quadricyclane couples due to the higher molecular weight.^7^

**Fig. 5:**
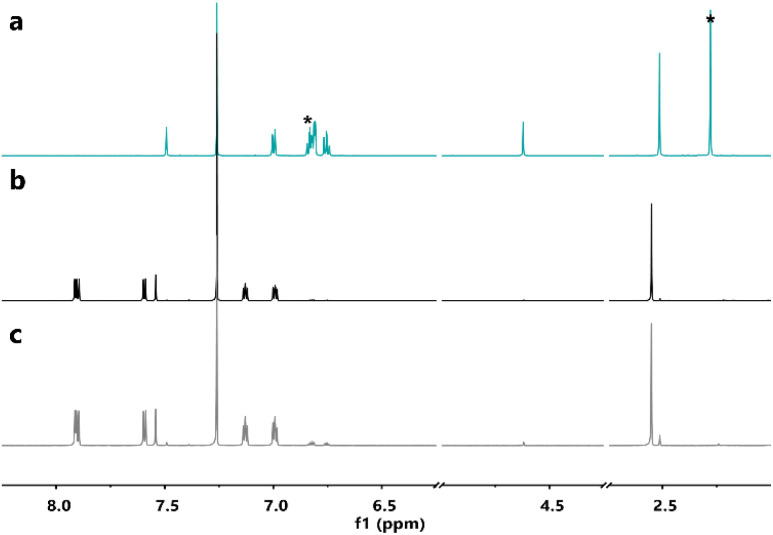
^1^H-NMRs in CDCl_3_ of (a, blue-green solid line) 4c after irradiation at 365 nm in non-degassed mesitylene; (b, black solid line) 4o before irradiation at 365 nm in non-degassed mesitylene; and (c, grey solid line) recovered 4o after the DSC measurement. *: peaks of residual mesitylene.

Furthermore, ^1^H-NMR spectroscopy of the samples after the DSC measurements indicated that the parent molecules could be recovered without degradation (Fig. 5, S5 and S6†), confirming a clean solid-state back-reaction.

The high temperatures required for back-conversion indicate that the photoisomers of the five investigated *ortho*-dianthrylbenzenes are not particularly thermally labile.

Kinetic studies in mesitylene (“Kinetic studies” section in the ESI†) were carried out to determine the half-lives of the photoisomers (Table 2), confirming that 1c–5c are stable for several years at room temperature, suggesting their suitability for long-term energy storage. Notably, 2c and 5c appeared to be significantly more stable than reference 1c, with half-life times of 25 years and 37 years at 25 °C, respectively, compared to 15 years, while the half-life of the *ortho*-xylene-linked 4c dropped to 9 years.

Having determined all the required parameters, the solar energy storage efficiencies (*η*_MOST_) of the investigated *ortho*-dianthrylbenzenes were calculated (Table 2 and “Solar energy storage efficiency” section in the ESI†) assuming no competitive absorption of the photoisomers 1c–5c between 300 nm and *λ*_onset_ (UV-vis spectra in Fig. 2 and S1†). *η*_MOST_ ranging from 0.38% for 3 to 0.66% for 1 are on par with those of some azo-based MOST systems (0.11% to 0.43% for azobenzenes, 0.59% to 0.94% for azopyrazoles)^48^ and norbornadiene-quadricyclane couples (0.51–0.7%),^7^ although somewhat lower than the recent record solar capture efficiency for NBDs (2.3%).^49^ Analysing eqn S2† (“Solar energy storage efficiency” section in the ESI†) in context of 1–5, absorbance and onset of absorption are attractive properties of these systems, but the relatively low *Φ*o–c (from 11.5% to 16%) are the main factor reducing solar capture efficiency, illustrating that future improvements in the photoisomerization quantum yield could significantly boost this parameter.

To complement the synthesis and experimental investigation of the considered *ortho*-dianthrylbenzenes, a computational investigation of the primary properties desired for a MOST system was also carried out. A GFN2-xTB-based^50^ screening approach was used to identify the lowest-energy conformers. Subsequent geometry optimizations were performed using r^2^SCAN-3c^51^ and ωB97X-D3(BJ)^52^ to determine the energy storage capacity of the given systems. The minima obtained using ωB97X-D3(BJ) were used for a climbing image nudged elastic band (CI-NEB) search,^53^ utilizing GFN2-xTB, to identify the transition states. For the resulting transition states a frequency analysis using ωB97X-D3(BJ) was carried out. Additionally, absorption spectra were simulated for the open and closed states of the photoswitches to evaluate their overlap with the solar spectrum. The conformer search was conducted using an in-house script linked to the xtb program package (Release 6.7.0),^54^ while all subsequent calculations were carried out in ORCA (Release 5.0.1).^55^ This overall approach aligns with earlier work on other organic photoswitches for MOST applications^56–58^ and is described in-depth in the ESI† (“Theoretical modelling details” section).

The experimental storage energies and related densities display significant variation in the energy storage capabilities of the selected systems under the employed conditions. Compared to the experimental values, the computed values in vacuum are generally lower, ranging from 63.4 kJ mol^−1^ to 68.7 kJ mol^−1^ (Table 2). This discrepancy can be attributed to the idealized conditions assumed in the calculations, which do not fully capture the complexity of the environment present in the experiments. This may also explain—at least partially—that the variation in the computed values is less pronounced than in the experimental ones. When implicit mesitylene is included, the computed values are slightly adjusted but still remain lower than the experimental values. The range in this case (63.4 kJ mol^−1^ to 67.7 kJ mol^−1^) indicates a modest impact of the solvent environment on the computed Gibbs free storage energy. The solvent appears to slightly lower the energy values, particularly for the 2o and 4o systems.

The predicted energy barriers for both the initial switching and the back-conversion are higher than expected. They all lie relatively close to each other, with the predicted reaction barriers in the range of 304.4 to 312.7 kJ mol^−1^, while the back-reaction barriers lie in the range of 241.0 to 245.0 kJ mol^−1^, as presented in Table S1.† Although higher than expected, the predicted values align well with the DSC results, indicating a thermally stable set of photoswitches. This agreement extends to the measured kinetics, despite the lack of a pronounced discrepancy between the highest and lowest barriers.

As for the storage energies, the inclusion of implicit mesitylene only has a modest impact on the predicted values. Notably, according to the CI-NEB search, the switching mechanism is stepwise regarding bond formation. Specifically, for each system, the transition is initiated by the formation of a bond between the two carbons directly connected to the linker, *i.e.*, a C9–C9′ single bond, as shown in Fig. S21 in the ESI.† This is consistent with the X-ray structures (“Crystal structure details” section in the ESI†), where the distance between those carbon atoms is around 3.06 Å, and the C10 and C10′ atoms are about 6.28–6.36 Å from each other. This indicates that the transition state of each system can be characterized by the presence of only a single bond. The corresponding distances in the theoretical transition states are 1.70 to 1.71 Å for the C9–C9′ bond and 3.00 to 3.02 Å for the C10–C10′ bond, as presented in Table S2.† Although interesting, the stepwise transition from the open to closed state makes the transition state search significantly more challenging, as most attempts result in a minimum—if they converge.

**Fig. 6:**
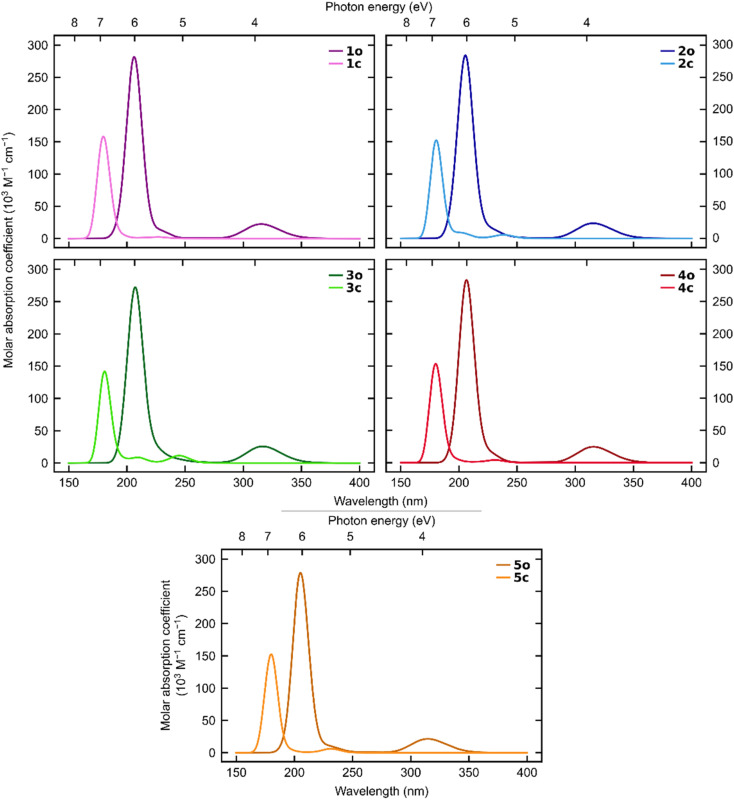
Simulated absorption spectra for 1–5 at the ωB97X-D3(BJ)/aug-cc-pVDZ level of theory.

The simulated UV-vis spectra (Fig. 6) show a clear separation between the open and closed states regarding absorption of visible light for all five systems.

Although both states absorb light below 270 nm, only the open states absorb at longer wavelengths. This demonstrates that all five *ortho*-dianthrylbenzenes are theoretically capable of absorbing sunlight and then storing it without undergoing a competitive photo-induced back-reaction. This finding aligns with the experimentally observed behaviour. This is a desirable trait for a potential MOST system, and in combination with the relatively high storage capacity, indicates that further investigation of anthracene-based photoswitches for MOST applications may be worthwhile.

Aiming to investigate any possible photo-induced isomerization in a condensed state, single crystals of 3o and 5o were grown through liquid–liquid diffusion in dichloromethane and methanol (“Crystal structure details” section in the ESI†). As reported in the literature for 1o, no formation of 3c and 5c was observed upon light irradiation.

**Fig. 7:**
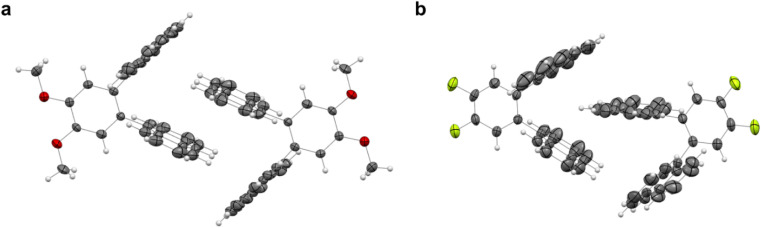
Crystal structures of (a) 3o and (b) 5o. Thermal ellipsoids were set at the 30% probability level and bonds were depicted in wireframe style.

This can be ascribed to the steric hindrance caused by the molecules' packing in the crystals (Fig. 7a and b). Indeed, the V-shaped *ortho*-dianthrylbenzenes appear to be intertwined because of the existing van der Waals forces between the anthracene moieties of two neighbour molecules, thus probably inhibiting any possible isomerization. 3o also shows C–H⋯π bonds.

**Fig. 8:**
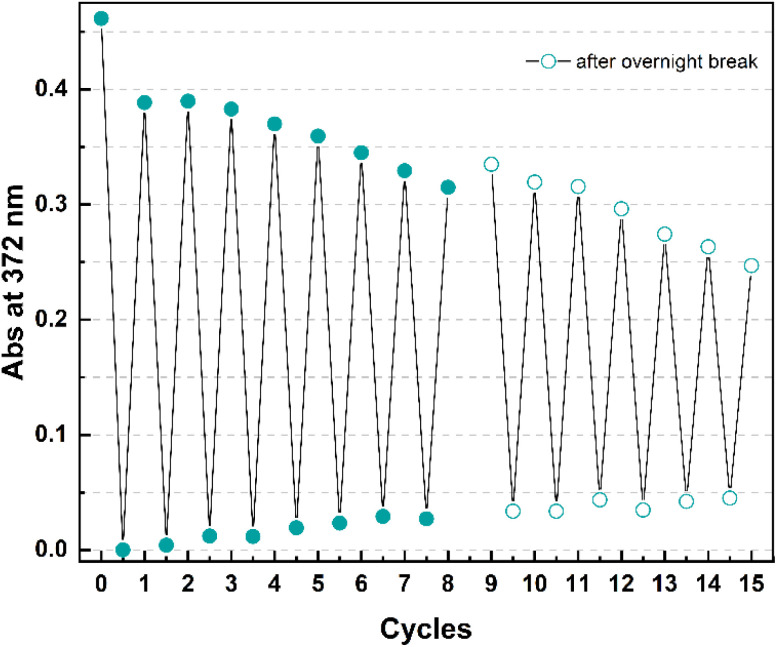
Cyclability study for 4 in mesitylene showing the absorption change at 372 nm upon 15 irradiation (365 nm, 2 min) – heating (130 °C, 1 h) cycles. After the 8th cycle, the experiment was stopped. A gap is present between cycles 8 and 9 because the overnight break led to some evaporation which required the addition of fresh mesitylene to re-adjust the concentration before the restart of the experiment.

Finally, cyclability experiments were carried out for 4 because of its shortest half-life time within the series. Fifteen irradiation – heating cycles were performed in mesitylene (Fig. 8) by irradiating a solution of 4o at 365 nm at room temperature for 2 minutes and then by heating it at 130 °C for 1 hour.

After a drop in absorption of around 16% with the first cycle (that can also be partially ascribed to a non-quantitative conversion in 1 h), the following photoisomerization – backreaction sequences occurred without as significant degradation (*e.g.* 0.08% in cycle 2, 0.5% in cycle 3). As the cycles were carried out manually, the experiment was stopped after eight cycles and continued the following day.

Having observed a slight evaporation of solvent leading to an increase in the absorbance, fresh mesitylene was added in the attempt to re-adjust the concentration to what it was at the end of the 8th cycle before performing the other irradiation-heating sequences. At the end of the experiment, an overall decrease in absorbance of 50% *ca.* was observed.

## Conclusions

In summary, five *ortho*-dianthrylbenzenes were synthesized and evaluated for molecular solar thermal (MOST) energy storage. The introduction of electron-donating or -withdrawing substituents on 1,2-di(anthryl)benzene 1o slightly impacted the optical properties of the switches but significantly influenced the thermal stability of the photogenerated isomers, particularly in the case of difluoro-functionalized 5c (*t*_1/2_ = 37 years). The observed storage energy values between around 65 and 94 kJ mol^−1^ and the corresponding energy storage densities (0.14–0.20 MJ kg^−1^), combined with their solar energy storage efficiencies ranging from 0.38% to 0.66%, make these photoswitches potentially suitable for MOST applications and open the door towards future developments on anthracene-based photochromes in this field. Next, these di(anthryl)benzenes will be further investigated to boost their *Φ*o–c (which appears as the main factor to be improved to achieve better MOST properties), evaluate the impact of the linker ring-size, and achieve on-demand energy release, for example through application of external stimuli different from temperature.

## Data availability

The data supporting this article have been included as part of the ESI.†

## Author contributions

N. B. performed the synthesis of the investigated switches and their preliminary characterization by UV-vis spectroscopy. L. M. M. completed the characterization of the photoswitches by determining their isomerization quantum yields, by carrying out the kinetics studies, the DSC and NMR experiments to study their back-conversion and the cyclability experiment. N. B. and J. L. E. performed the real solar conversion experiment to investigate the isomerization under unfiltered sunlight. Z. L. and J. L. E. carried out the computational modelling of the systems and their properties. J. L. E. calculated the solar energy storage efficiencies. K. V. M. supervised the computational part of the project. P. B. and N. B. measured the fluorescence quantum yields. E. M. solved the single crystal structures. N. B. and K. M. P. designed the project. K. M. P. supervised the project and was responsible for funding acquisition. N. B. prepared the original draft. All the authors contributed to the review and editing of the original draft.

## Conflicts of interest

There are no conflicts to declare.

## Supplementary Material

TA-012-D4TA03879G-s001

TA-012-D4TA03879G-s002
