# Supplementary material for: Exploring ortho-dianthrylbenzenes for molecular solar thermal energy storage
Source: J Mater Chem A Mater. 2024 Aug 15;12(39):26457–64. doi: 10.1039/d4ta03879g (PMC11350467; doi:10.1039/d4ta03879g)
Supplement: TA-012-D4TA03879G-s001 [file TA-012-D4TA03879G-s001.pdf]

Supplementary Information (SI) for Journal of Materials Chemistry A.
This journal is © The Royal Society of Chemistry 2024

Supplementary Information for

Exploring *ortho*-dianthrylbenzenes for Molecular Solar Thermal Energy Storage

Nicolò Baggi,[ab] Lidiya M. Muhammad,[c] Zacharias Liasi,[d] Jacob Lynge Elholm,[b] Paulius Baronas,[b] Elies Molins,[a] Kurt V. Mikkelsen,[d] and Kasper Moth-Poulsen[abce]

a) The Institute of Materials Science of Barcelona, ICMAB-CSIC

Bellaterra, 08193 Barcelona (Spain)

b) Department of Chemical Engineering, Universitat Politècnica de Catalunya, EEBE EduardMaristany 10–14, 08019 Barcelona (Spain)

c) Department of Chemistry and Chemical Engineering, Chalmers University of Technology

SE-41296 Gothenburg (Sweden)

d) Department of Chemistry, University of Copenhagen

Universitetsparken 5, Copenhagen Ø 2100 (Denmark)

e) Catalan Institution for Research & Advanced Studies, ICREA

Pg. Lluís Companys 23, 08010 Barcelona (Spain)

** E-mail: kasper.moth-poulsen@upc.edu*

Table of contents

Materials and equipment	– 2 –
Synthetic procedures	– 4 –
Additional experimental data	– 8 –
Determination of the photoisomerization quantum yields	– 15 –
Determination of the fluorescence quantum yields	– 19 –
Kinetic studies	– 21 –
Solar energy storage efficiency	– 26 –
Theoretical modelling details	– 27 –
Crystal structure details	– 30 –
^{1}H-, ^{13}C- and ^{19}F-NMR spectra	– 34 –
References	– 55 –

Materials and equipment

All the reagents and solvents were purchased from commercial suppliers and used as received unless otherwise stated.

Thin layer chromatography (TLC) was performed on silica gel 60 F254 while column chromatography was carried out on silica gel (70-230 mesh, 60-200 µm).

^{1}H-, ^{13}C-NMR and ^{19}F-NMR spectra were recorded using a Bruker Ascend 300 MHz - Console nano NEO or a Bruker Avance NEO 600 MHz or an Oxford 800 magnet, Bruker Avance III HD 800 MHz spectrometer. Eurisotop deuterated chloroform ($CDCl_3$) was used to prepare the samples and its residual proton signal was used as standard (7.26 ppm). Chemical shifts are provided in δ (ppm) and the coupling constants J in Hz. The splitting patterns are indicated as: s (singlet); d (doublet); dd (doublet of doublets); m (multiplet).

High-resolution mass spectra were obtained using a Bruker MicroTof-Q spectrometer with an ESI (ElectroSpray Ionization) ionization source.

Crystallographic data for **3o** and **5o** were collected on a Bruker APEX-II CCD diffractometer at 294(2) K, using a graphite monochromated Mo Kα radiation (λ = 0.71073 Å). Data reduction was performed using SAINT V6.45A and SORTAV in the diffractometer package.[1] Data were corrected for Lorentz and polarization effects and for absorption by SADABS.[2] The structural resolution procedure was made using SHELXT.[3] Non-hydrogen atoms were refined using anisotropic thermal parameters. Hydrogen atoms were introduced in calculated positions and refined riding on their parent atoms. Crystal data and refinement parameters are shown in Tables S3 and S4. ORTEP views of the molecules can be seen at Fig. S22 and Fig. S23. Complete crystallographic data for the structural analysis have been deposited with the Cambridge Crystallographic Data Centre, CCDC nº 2359712 and 2359711, respectively. Torsion angles between the anthracenes and the bridging group range from 80.3 to 95.7º for **3o** and from 78.6 to 97.8º for **5o**. **3o** crystal packing shows several C-H···π bonds, while **5o** presents two C-H···F short contacts, connecting the two independent molecules in the asymmetric unit.

UV-Visible spectroscopic measurements were performed in a quartz cuvette (optical path: 1 cm) on a Cary 60-UV-Vis spectrophotometer coupled with QNW Luma 40/E temperature control or a JASCO V-770 UV-Vis-NIR spectrophotometer. Irradiations at 365 nm were carried out with a 365 nm LED by Thorlabs, Inc.

The spectral evolution upon exposition to unfiltered sunlight was monitored on an Avantes AvaSpec-ULS2048CL-EVO-RS-UA UV-Vis spectrophotometer. To achieve the irradiation, a quartz cuvette (optical path: 1 cm) containing a solution of **4o** in mesitylene was exposed to unfiltered sunlight in Barcelona, Spain (on 10 July 2024, experiment started at around 5.15 pm and finished at 5.30 pm ca.).
Cycling experiment for **4** was manually tested by irradiating a quartz cuvette containing a mesitylene solution of **4o** at 365 nm at room temperature for 2 minutes and then by heating the cuvette at 130°C for 1 hour. Once cooled down to room temperature, the sample was irradiated and heated again.
The quantum yield of photoisomerization was determined in mesitylene using potassium ferrioxalate as a chemical actinometer.[4,5] Details can be found in section "Determination of photoisomerization quantum yields".

Fluorescence spectra were recorded on an Avantes AvaSpec-ULS2048CL-EVO-RS-UA UV-Vis spectrophotometer. Photoluminescence quantum yields were determined employing relative quantum method using 9,10-diphenylanthracene in 1 μM toluene solution (Φ_F = 0.85) as a reference standard.[6] Details on the measurements are provided in section "Determination of fluorescence quantum yields".

Differential scanning calorimetry (DSC) were carried out on a Mettler Toledo DSC 5+ apparatus on 1-2 mg ca. of photoisomers **1c** – **5c** sealed in a 40 μL aluminum DSC pan. The photoisomers were previously prepared by irradiation at 365 nm of **1o** – **5o** in mesitylene (or DMSO) and isolated by solvent removal through Biotage® V-10 Touch. After irradiation, **3c** and **4c** precipitated as white solids while **1c**, **2c**, and **5c** remained completely dissolved in mesitylene. During the DSC experiments, samples were first heated from 25°C to 80°C at a 6°C min^{-1} heating rate, then until 280°C at a 1°C min^{-1} heating rate. The heating was followed by cooling to 25°C at a -30°C min^{-1} cooling rate. Next, a second cycle of heating and cooling was done at a 1°C min^{-1} heating rate and a -50°C min^{-1} cooling rate. For all cases, a heat release was observed only in the first cycle of heating (Figure S3 – S4).

Thermogravimetric analyses (TGA) were performed on a Mettler Toledo TGA/DSC 3+ apparatus by heating 1-3 mg ca. of photoisomers **1c** – **5c**, previously weighed on a 70 μL alumina crucibles, from 25°C to 500°C at a 10°C min^{-1} heating rate. The TGA results (Figure S7) showed that all the photoisomers are stable at elevated temperatures, and in all cases a weight loss between 70°C and 130°C was observed, which can be attributed to the evaporation of residual solvent traces.

Synthetic procedures

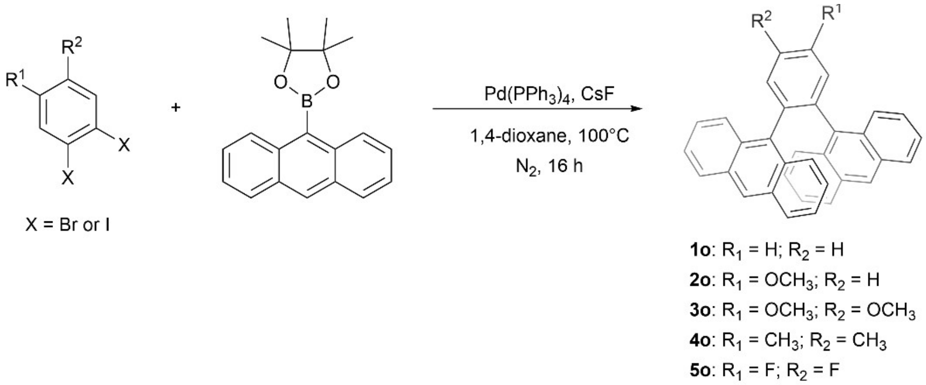

Scheme S1: Synthetic route to prepare **1o** – **5o**.

*Synthesis of **1o***

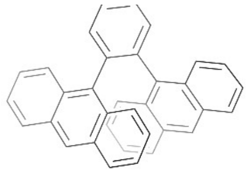

1o

1,2-diiodobenzene (247.4 mg, 0.98 mL, 0.75 mmol), 9-anthraceneboronic acid pinacol ester (548 mg, 1.80 mmol), CsF (570 mg, 3.75 mmol) and $Pd(PPh_3)_4$ (46 mg, 0.04 mmol) were partially solubilized in dry 1,4-dioxane (10 mL) and the obtained yellow mixture was stirred at reflux under nitrogen for 16 h. The reaction was quenched with water (20 mL) and extracted with chloroform. The combined extracts were washed with water, dried over anhydrous Na_2SO_4, and filtered. The filtrate was concentrated under vacuum and hexane was added to the residue. The crude product was isolated by filtration and then purified by column chromatography (solid loading; eluent: from hexane to hexane / dichloromethane 7 : 3). After a wash with a minimum amount of Et_2O, pure **1o** (50 mg, 0.12 mmol, 15%) was isolated as a white crystalline solid. Characterization data consistent with the literature.

^{1}H-NMR ($CDCl_3$, 300 MHz): δ (ppm) 7.92 (s, 2H), 7.84 – 7.81 (m, 4H), 7.79 (s, 4H), 7.61 – 7.58 (m, 4H), 7.16 – 7.10 (m, 4H), 7.01 – 6.96 (m, 4H).
^{13}C-NMR ($CDCl_3$, 201 MHz) δ (ppm) 140.0, 135.6, 133.6, 130.7, 129.9, 127.9, 127.8, 127.6, 126.3, 124.5, 124.3.
HRMS (ESI+): calcd. for $C_{34}H_{23}^+$ $[M+H]^+$ 431.1794, found $[M+H]^+$ 431.1779.

*Synthesis of **2o***

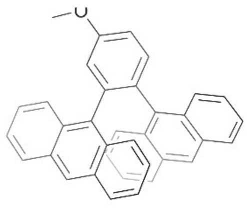

2o

1,2-dibromo-4-methoxybenzene (200 mg, 0.75 mmol), 9-anthraceneboronic acid pinacol ester (580 mg, 1.90 mmol), CsF (685 mg, 4.51 mmol) and $Pd(PPh_3)_4$ (174 mg, 0.15 mmol) were partially solubilized in dry 1,4-dioxane (10 mL) and the obtained yellow mixture was stirred at reflux under nitrogen for 16 h. The reaction was quenched with water (20 mL) and extracted with chloroform. The combined extracts have been washed with water, dried over anhydrous Na_2SO_4, and filtered. The filtrate was concentrated under vacuum and methanol was added to the residue. The crude product was isolated by filtration. Recrystallization with dichloromethane, toluene and methanol allowed to make the remaining impurities precipitate while keeping the product in solution. After filtration and removal of the solvents, pure **2o** (125 mg, 0.27 mmol, 36%) was isolated as a yellow crystalline solid.

^{1}H-NMR ($CDCl_3$, 300 MHz): δ (ppm) 7.91 – 7.86 (m, 6H), 7.70 – 7.67 (m, 1H), 7.58 (d, *J* = 8.5 Hz, 4H), 7.36 – 7.33 (m, 2H), 7.16 – 7.10 (m, 4H), 7.04 – 6.96 (m, 4H).
^{13}C-NMR ($CDCl_3$, 201 MHz) δ (ppm) 134.6, 130.8, 130.2, 129.8, 127.9, 127.7, 127.6, 126.4, 126.1, 124.6, 124.5, 124.4, 124.2, 118.5, 113.7, 55.7.
HRMS (ESI+): calcd. for $C_{35}H_{24}NaO^+$ $[M+Na]^+$ 483.1719, found $[M+Na]^+$ 483.1736.

*Synthesis of **3o***

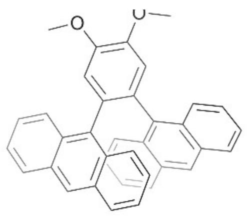

3o

1,2-dibromo-4,5-dimethoxybenzene (200 mg, 0.68 mmol), 9-anthraceneboronic acid pinacol ester (610 mg, 2.00 mmol), CsF (517 mg, 3.40 mmol) and $Pd(PPh_3)_4$ (81 mg, 0.07 mmol) were partially solubilized in dry 1,4-dioxane (10 mL) and the obtained yellow mixture was stirred at reflux under nitrogen for 16 h. The reaction was quenched with water (20 mL) and extracted with chloroform. The combined extracts were washed with water, dried over anhydrous Na_2SO_4, and filtered. The

filtrate was concentrated under vacuum and the residue was purified by column chromatography (solid loading; eluent: from hexane to hexane / dichloromethane 7 : 3). Upon recrystallization in dichloromethane and methanol, pure **3o** (71 mg, 0.15 mmol, 21%) was isolated as a yellow crystalline solid. Single crystals suitable for XRD diffraction were also obtained. Characterization data consistent with the literature.

^{1}H-NMR ($CDCl_3$, 300 MHz): δ (ppm) 7.95 – 7.91 (m, 6H), 7.60 (d, *J* = 7.8 Hz, 4H), 7.28 (s, 2H), 7.17 – 7.12 (m, 4H), 7.05 – 6.99 (m, 4H), 4.01 (s, 6H).
^{13}C-NMR ($CDCl_3$, 201 MHz) δ (ppm) 148.3, 135.5, 132.1, 130.8, 130.1, 128.0, 127.7, 126.3, 124.6, 124.4, 116.2, 56.4.
HRMS (ESI+): calcd. for $C_{36}H_{26}NaO_2^+$ $[M+Na]^+$ 513.1785, found $[M+Na]^+$ 513.1815.

*Synthesis of **4o***

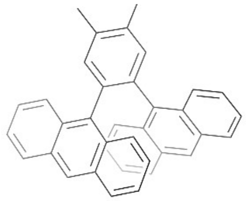

4o

1,2-dibromo-4,5-dimethylbenzene (200 mg, 0.76 mmol), 9-anthraceneboronic acid pinacol ester (578 mg, 1.90 mmol), CsF (693 mg, 4.56 mmol) and $Pd(PPh_3)_4$ (176 mg, 0.15 mmol) were partially solubilized in dry 1,4-dioxane (10 mL) and the obtained yellow mixture was stirred at reflux under nitrogen for 16 h. The reaction was quenched with water (20 mL) and extracted with chloroform. The combined extracts have been washed with water, dried over anhydrous Na_2SO_4, and filtered. The filtrate was concentrated under vacuum and methanol was added to the residue. The crude product was isolated by filtration. Recrystallization with dichloromethane and methanol followed by washing of the filtered crystals with Et_2O allowed to isolate pure **4o** (105 mg, 0.23 mmol, 30%) as a yellow crystalline solid.

^{1}H-NMR ($CDCl_3$, 300 MHz): δ (ppm) 7.91 – 7.89 (m, 6H), 7.59 (d, *J* = 8.3 Hz, 4H), 7.54 (s, 2H), 7.15 – 7.10 (m, 4H), 7.02 – 6.96 (m, 4H), 2.55 (s, 6H).
^{13}C-NMR ($CDCl_3$, 201 MHz) δ (ppm) 137.2, 136.0, 135.9, 134.6, 130.8, 130.0, 127.9, 127.8, 126.0, 124.5, 124.2, 19.9.
HRMS (ESI+): calcd. for $C_{36}H_{26}Na^+$ $[M+Na]^+$ 481.1927, found $[M+Na]^+$ 481.1928.

Synthesis of ***5o***

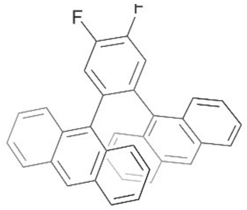

5o

1,2-dibromo-4,5-difluorobenzene (200 mg, 0.73 mmol), 9-anthraceneboronic acid pinacol ester (560 mg, 1.84 mmol), CsF (670 mg, 4.41 mmol) and $Pd(PPh_3)_4$ (173 mg, 0.15 mmol) were partially solubilized in dry 1,4-dioxane (10 mL) and the obtained yellow mixture was stirred at reflux under nitrogen for 16 h. The reaction was quenched with water (20 mL) and extracted with chloroform. The combined extracts have been washed with water, dried over anhydrous Na_2SO_4, and filtered. The filtrate was concentrated under vacuum and methanol was added to the residue. The crude product was isolated by filtration. Recrystallization with dichloromethane and methanol allowed to isolate pure **5o** (182 mg, 0.39 mmol, 53%) as a yellow crystalline solid. Single crystals suitable for XRD diffraction were also obtained.

^{1}H-NMR ($CDCl_3$, 300 MHz): δ (ppm) 7.93 (s, 2H), 7.80 (dd, *J* = 8.8, 1.0 Hz, 4H), 7.65 – 7.57 (m, 6H; superposed triplet peak for the 2H of the linker – coupled with F – and 4H doublet of doublets), 7.17 – 7.12 (m, 4H), 7.06 – 7.00 (m, 4H).
^{13}C-NMR ($CDCl_3$, 201 MHz): δ (ppm) 136.9, 133.1, 130.7, 129.8, 128.1, 127.0, 126.9, 124.8, 124.7, 122.3, 122.2.
^{19}F-NMR ($CDCl_3$, 282 MHz) δ (ppm) -138.9 (t, *J* = 9.5 Hz).
HRMS (ESI+): calcd. for $C_{34}H_{20}F_2^+$ $[M]^+$ 466.1528, found $[M]^+$ 466.1543.

Additional experimental data

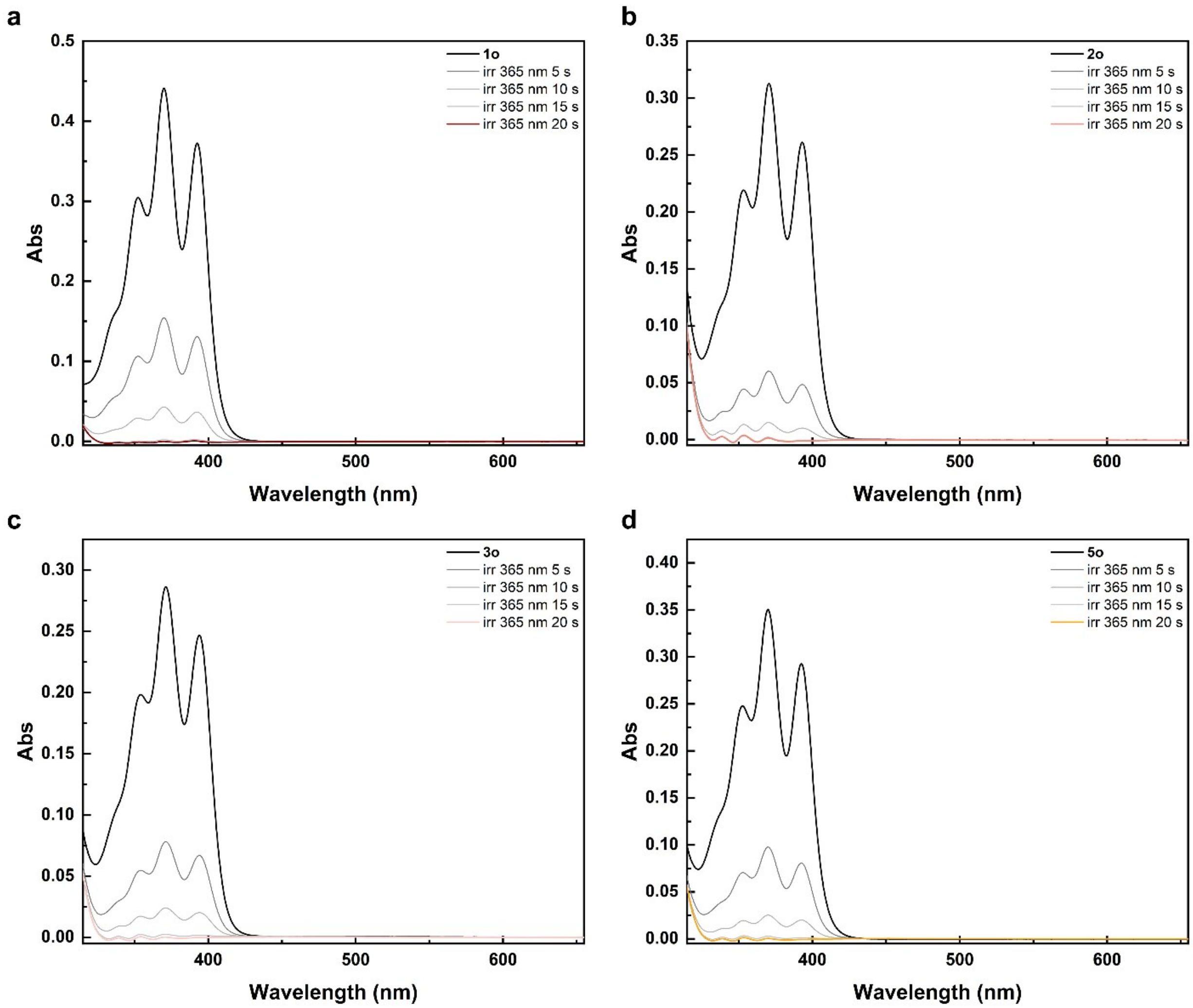

Figure S1: Spectral evolution of a) **2o** (1.9×10^{-5} M), b) **3o** (1.6×10^{-5} M), c) **4o** (2.2×10^{-5} M), and d) **5o** (2.3×10^{-5} M) in mesitylene under light irradiation (365 nm) at room temperature to their corresponding photoisomers.

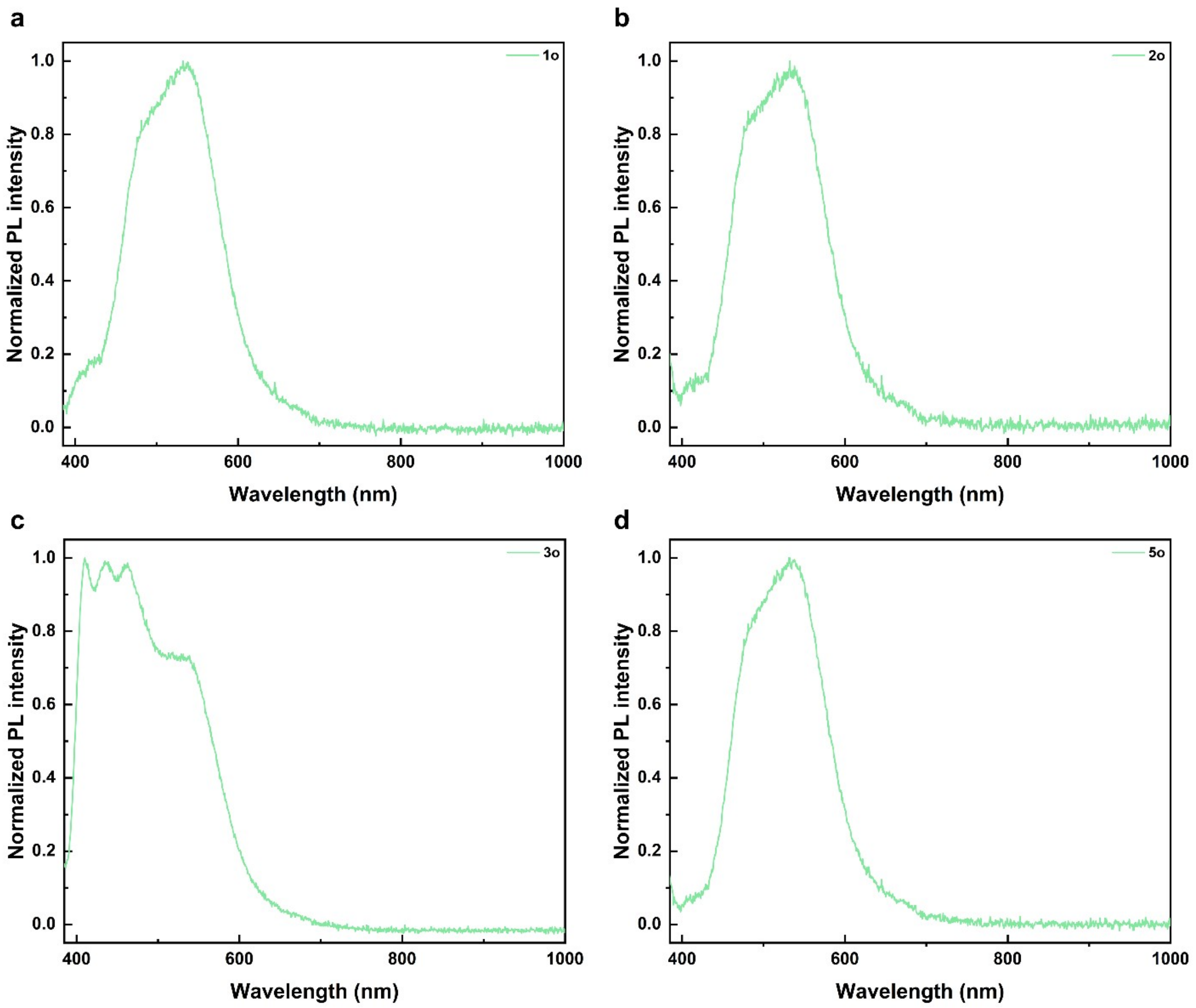

Figure S2: Normalized emission of a) **1o**, b) **2o**, c) **3o** and d) **5o** in mesitylene upon excitation at 365 nm.

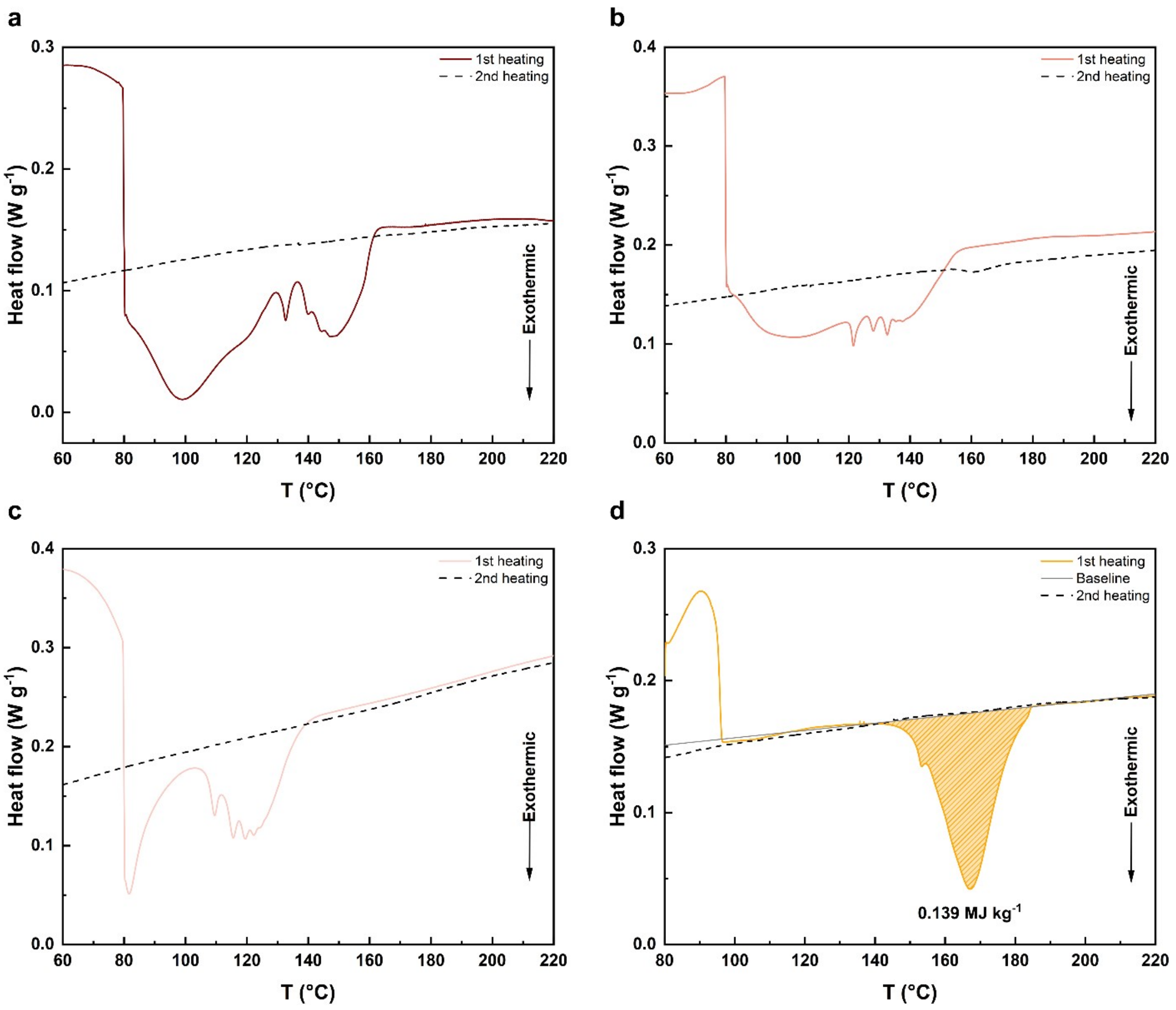

Figure S3: DSC graphs of a) **1c**, b) **2c**, c) **3c** and d) **5c** prepared by irradiation in mesitylene and showing the exothermic events occurring during the first heating cycles (coloured solid lines). No exothermic process was observed during the second heating cycles (black dashed lines). In the case of **5c**, an endothermic process (possibly its melting) is observed between 80°C and 100°C ca. and the heat release is observed between 140°C and 185°C ca.

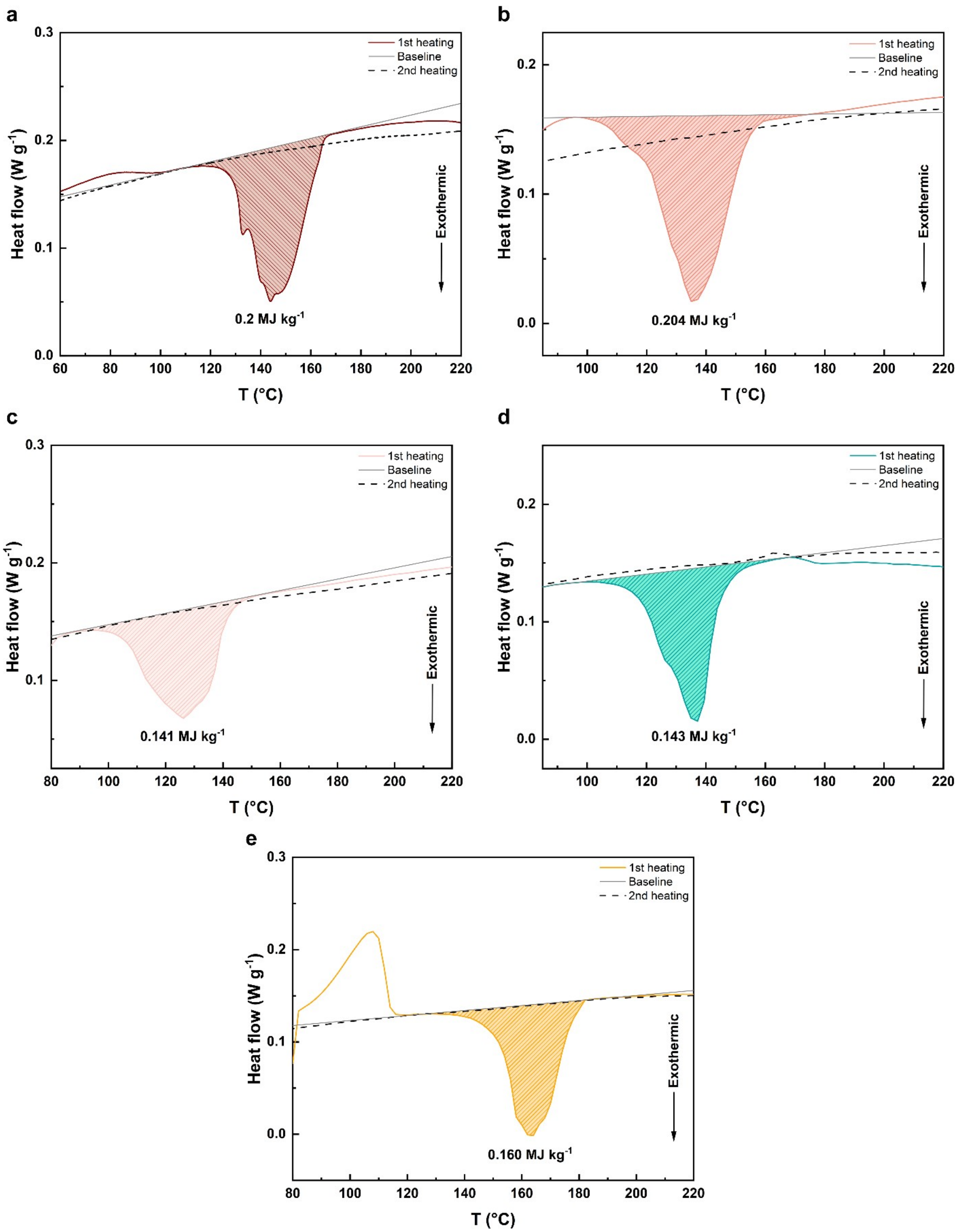

Figure S4: DSC graphs of a) **1c**, b) **2c**, c) **3c** and d) **4c** and e) **5c** prepared by irradiation in DMSO and showing the heat releases occurring during the first heating cycles (coloured solid lines). No

exothermic process was observed during the second heating cycles (black dashed lines). The baseline used for the integration to determine the energy releases is provided with solid grey lines for clarity. In the case of **5c**, and endothermic process (possibly its melting) is observed between 80°C and 110°C ca.

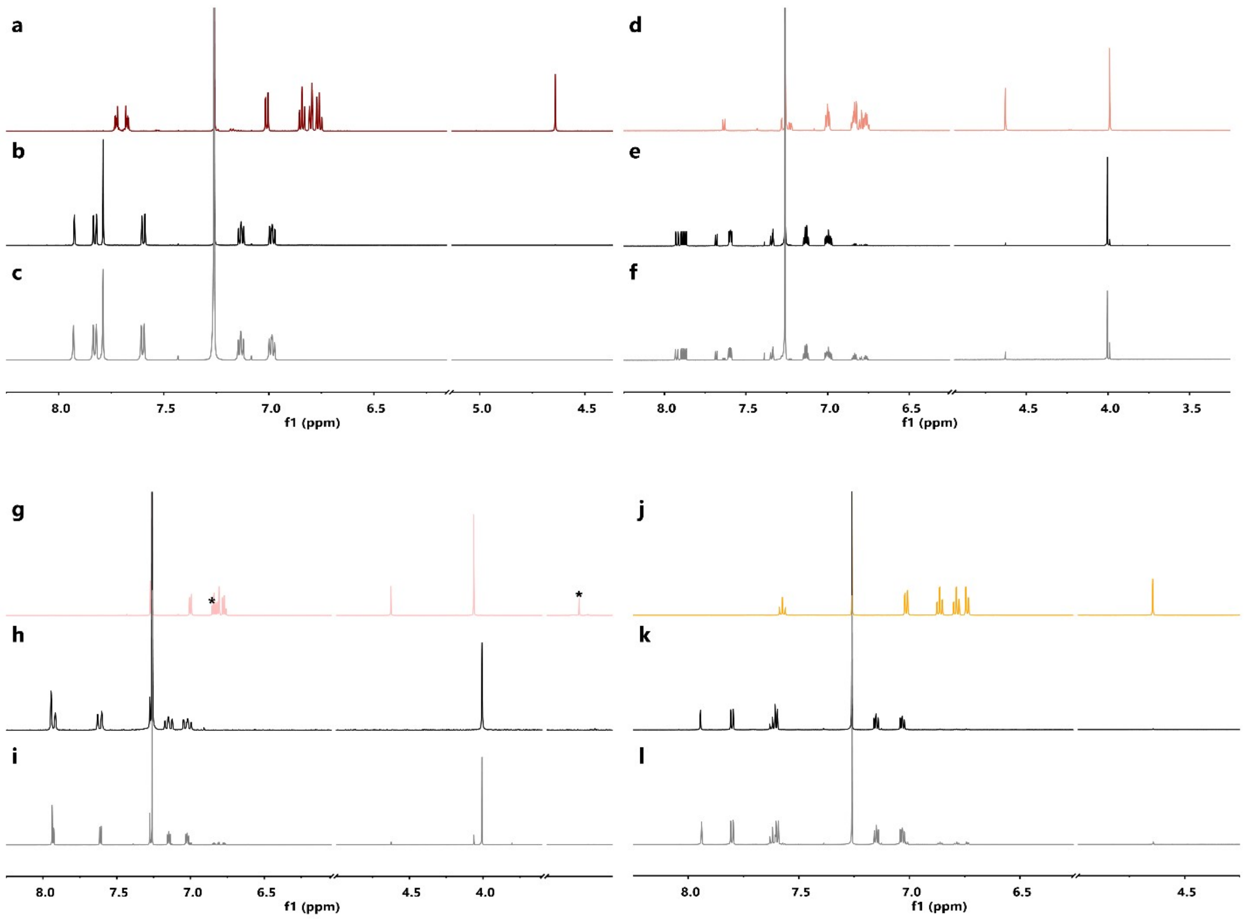

Figure S5: ^{1}H-NMRs in $CDCl_3$ of a) **1c** after irradiation at 365 nm in non-degassed mesitylene; b) **1o** before irradiation at 365 nm in non-degassed mesitylene; c) recovered **1o** after the DSC measurement; d) **2c** after irradiation at 365 nm in non-degassed mesitylene; e) **2o** before irradiation at 365 nm in non-degassed mesitylene; f) recovered **2o** after the DSC measurement; g) **3c** after irradiation at 365 nm in non-degassed mesitylene; h) **3o** before irradiation at 365 nm in non-degassed mesitylene; i) recovered **3o** after the DSC measurement; j) **5c** after irradiation at 365 nm in non-degassed mesitylene; k) **5o** before irradiation at 365 nm in non-degassed mesitylene; l) recovered **5o** after the DSC measurement. *: peaks of residual mesitylene.

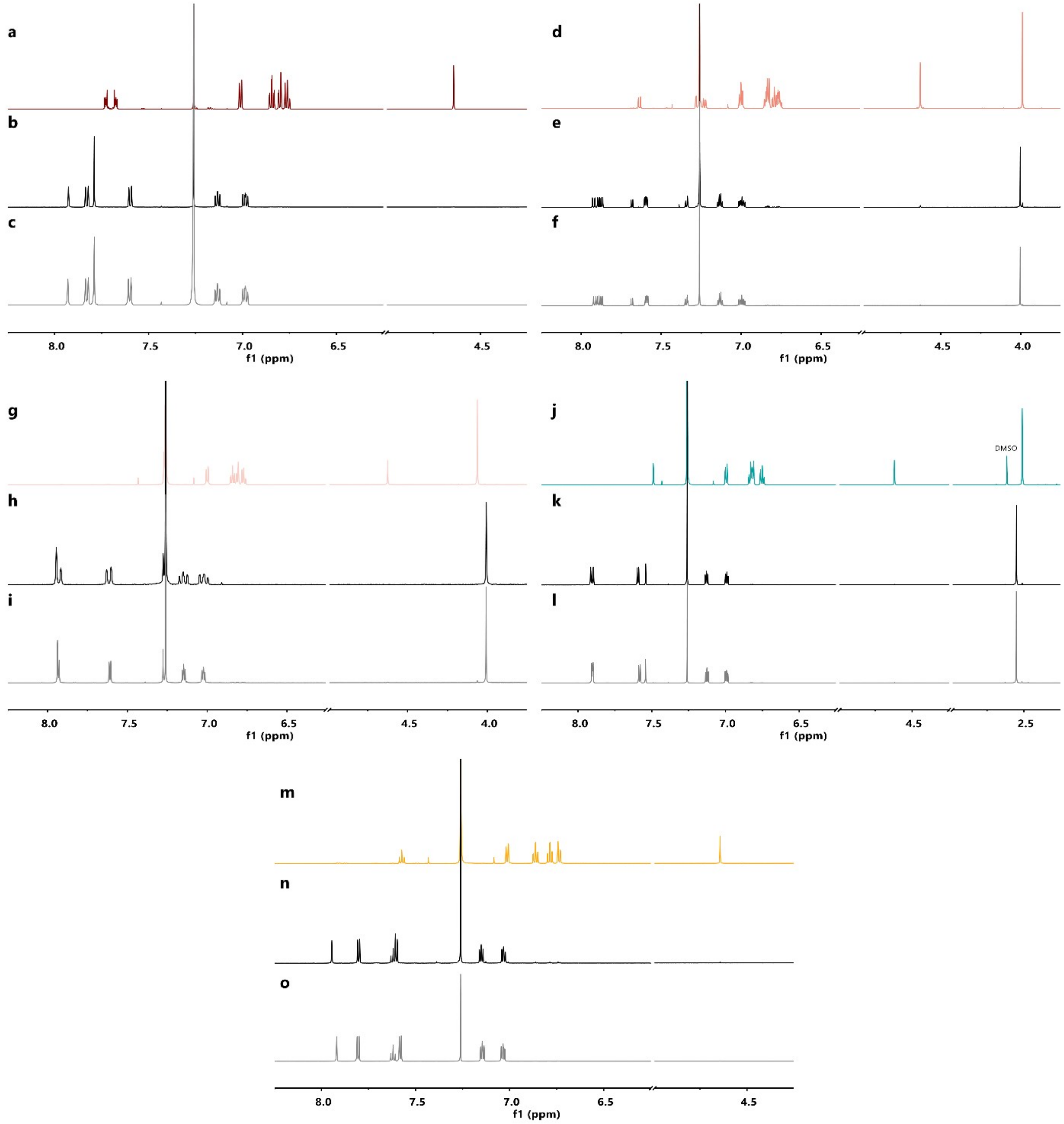

Figure S6: ^{1}H-NMRs in $CDCl_3$ of a) **1c** after irradiation at 365 nm in non-degassed DMSO; b) **1o** before irradiation at 365 nm in non-degassed DMSO; c) recovered **1o** after the DSC measurement; d) **2c** after irradiation at 365 nm in non-degassed DMSO; e) **2o** before irradiation at 365 nm in non-degassed DMSO; f) recovered **2o** after the DSC measurement; g) **3c** after irradiation at 365 nm in non-degassed DMSO; h) **3o** before irradiation at 365 nm in non-degassed DMSO; i) recovered **3o** after the DSC measurement; j) **4c** after irradiation at 365 nm in non-degassed DMSO; k) **4o** before irradiation at 365 nm in non-degassed DMSO; l) recovered **4o** after the DSC

measurement; m) **5c** after irradiation at 365 nm in non-degassed DMSO; n) **5o** before irradiation at 365 nm in non-degassed DMSO; o) recovered **5o** after the DSC measurement.

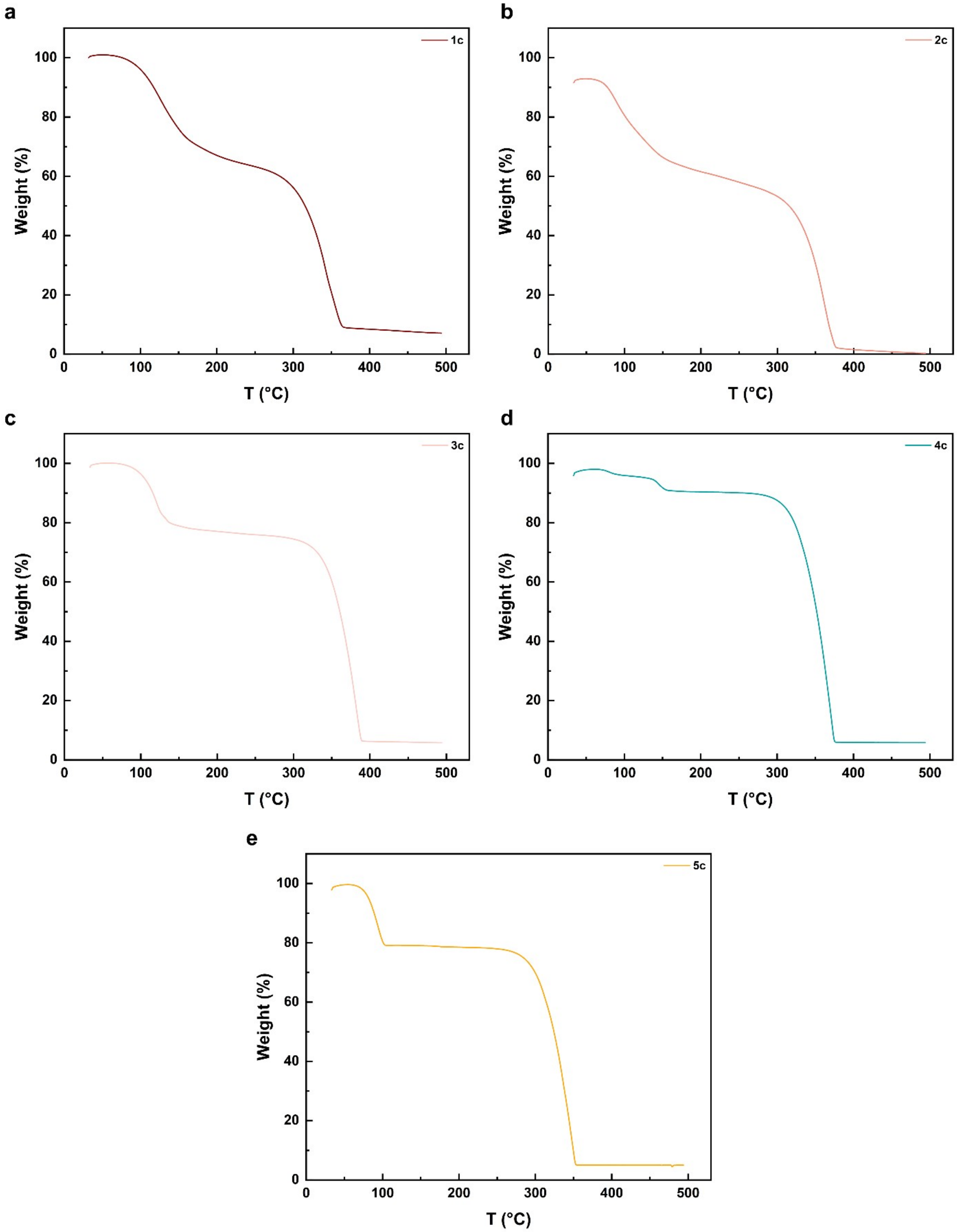

Figure S7: TGA graphs of a) **1c**, b) **2c**, c) **3c**, d) **4c** and e) **5c**.

Determination of the photoisomerization quantum yields

The experiments were conducted using a 365 nm light-emitting diode (Thorlabs M365LP1). The results presented in the article are an average of two measurements per photoswitch.

The photon flux of the light source was determined using potassium ferrioxalate actinometry both before and after the actual measurement (Fig. S8).[4,5] In order to determine the quantum yields, solutions of **1o** – **5o** in mesitylene (at concentrations guaranteeing absorption over 2 at 365 nm) were prepared and kept in the dark to prevent photoconversion before measurements. 2 mL of these solutions were then irradiated in a cuvette with a 365 nm LED, and the decrease in absorption was monitored using a UV-vis spectrophotometer. The quantum yields of photoisomerization were calculated using the reported equation in the literature.

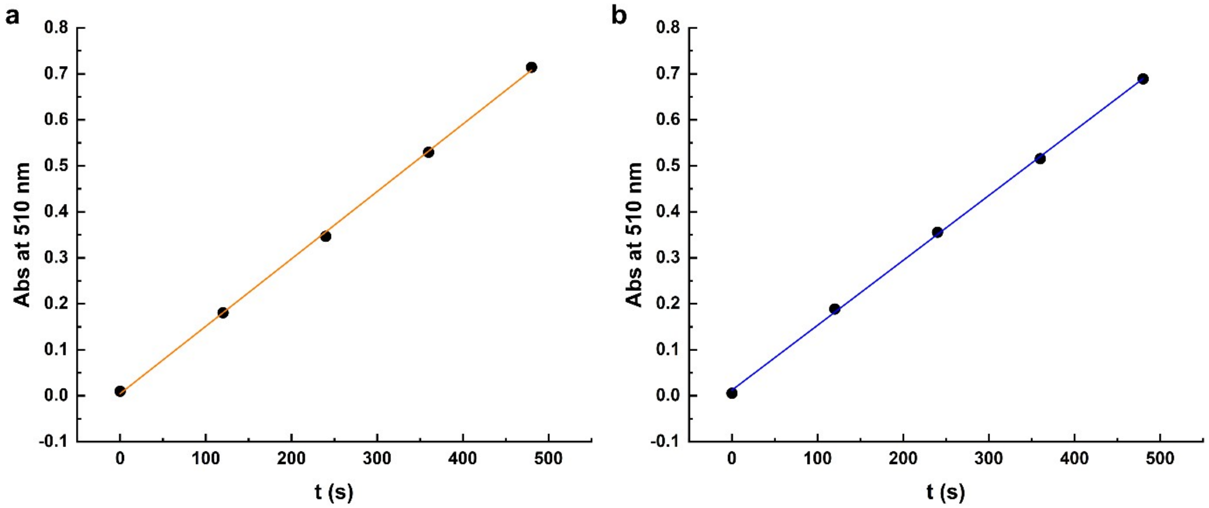

Figure S8: Photon flux for the LED 365 nm a) before (1.60×10^{-8} s^{-1}) and b) after (1.54×10^{-8} s^{-1}) the measurements on **1o** – **5o**.

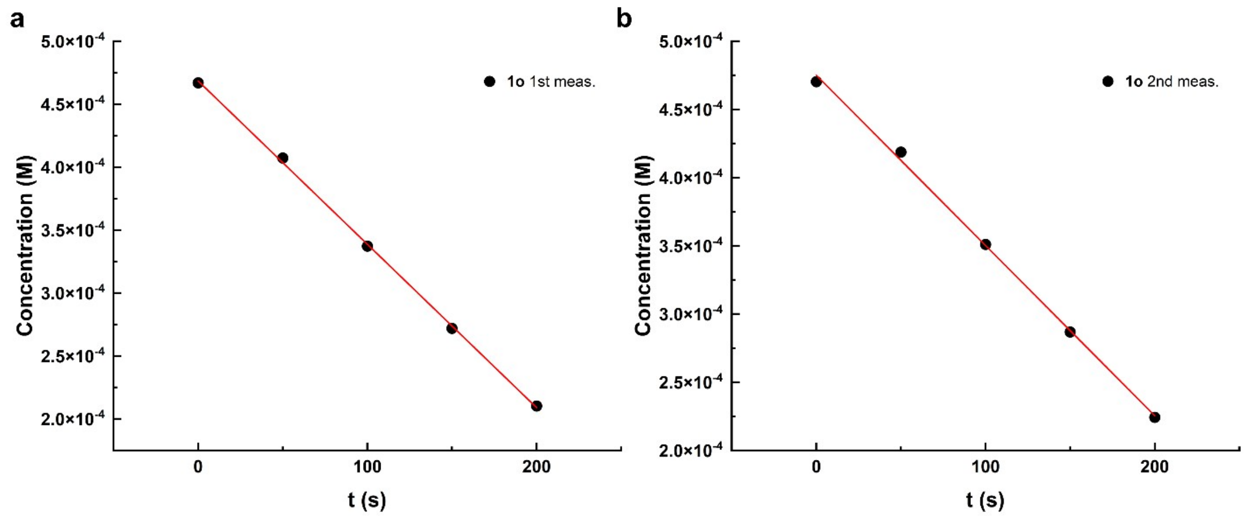

Figure S9: Plot of the quantum yield measurements for compound **1o**; a) exp. 1: Φ = 15.7% and b) exp. 2: Φ = 15.7%. Average Φ = 15.7% ---> 16%

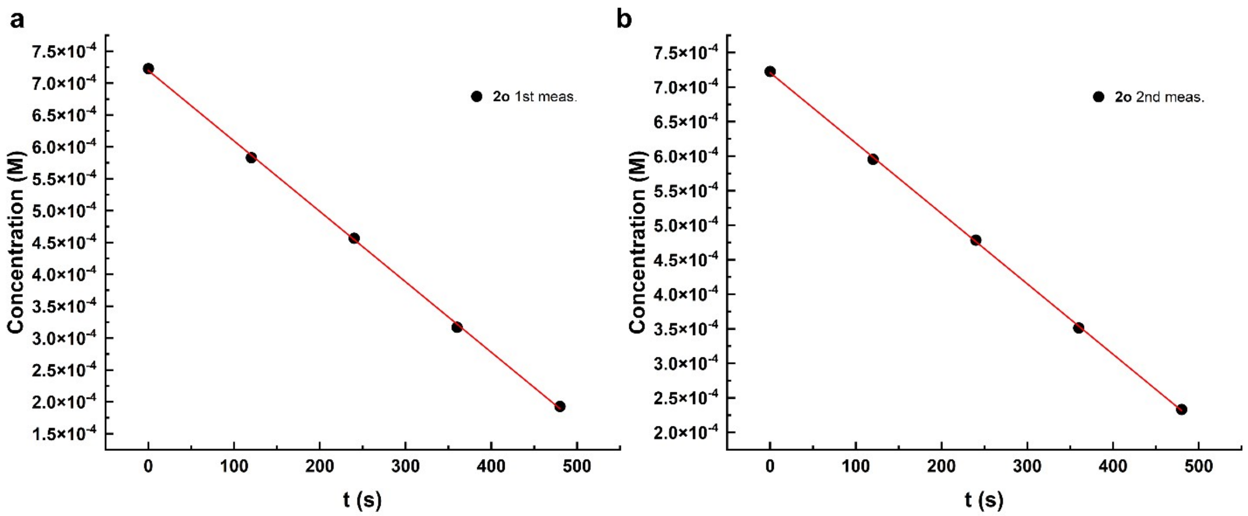

Figure S10: Plot of the quantum yield measurements for compound **2o**; a) exp. 1: Φ = 13.7% and b) exp. 2: Φ = 13.0%. Average Φ = 13.3% ---> 13%

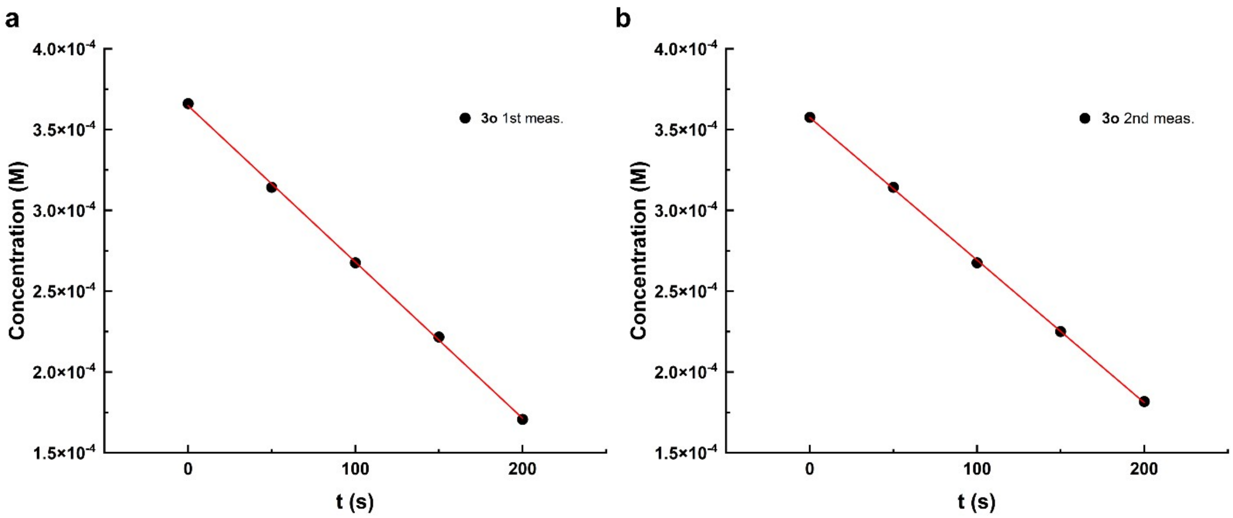

Figure S11: Plot of the quantum yield measurements for compound **3o**; a) exp. 1: Φ = 12.0% and b) exp. 2: Φ = 11.0%. Average Φ = 11.5%

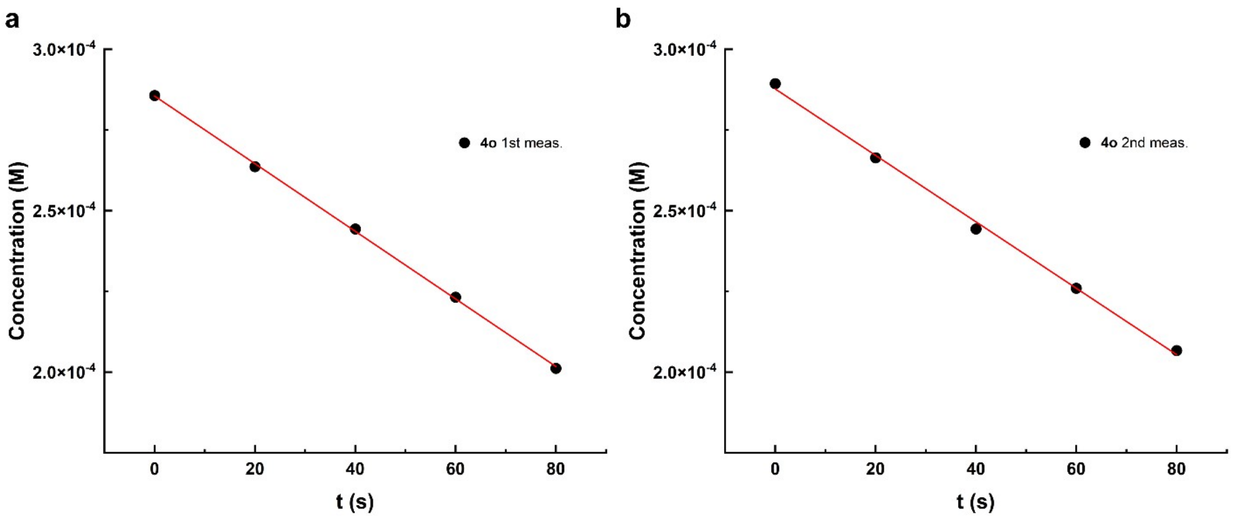

Figure S12: Plot of the quantum yield measurements for compound **4o**; a) exp. 1: Φ = 13.1% and b) exp. 2: Φ = 12.8%. Average Φ = 12.9% ---> 13%

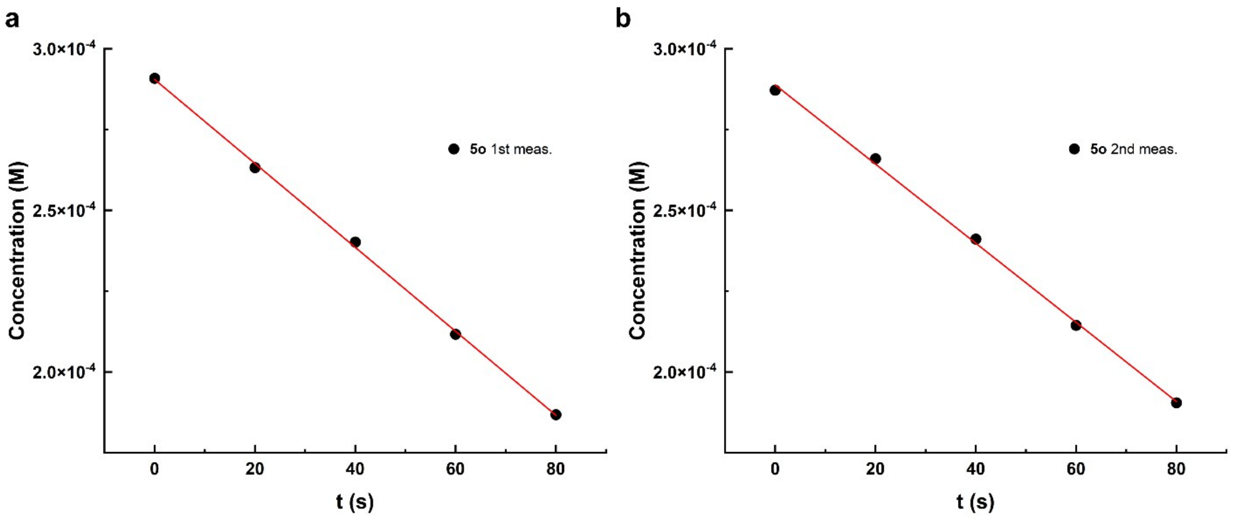

Figure S13: Plot of the quantum yield measurements for compound **5o**; a) exp. 1: Φ = 16.1% and b) exp. 2: Φ = 15.6%. Average Φ = 15.8% ---> 16%

Determination of the fluorescence quantum yields

Solution samples were excited with a 365 nm light emitting diode (Thorlabs M365FP1). Five to six solutions with different concentrations were used to determine the emission per unit of absorbed light of the samples, i.e. the slopes (m) of the integrated emission intensities vs absorbances lines. Φ_F values were then measured with the following equation (eq. S1):

$$\Phi_F = \Phi_F^s \frac{mn^2}{m_s n_s^2} \qquad (eq.\ S1)$$

Where "s" is used to refer to the standard and n is the refractive index.

9,10-diphenylanthracene in toluene

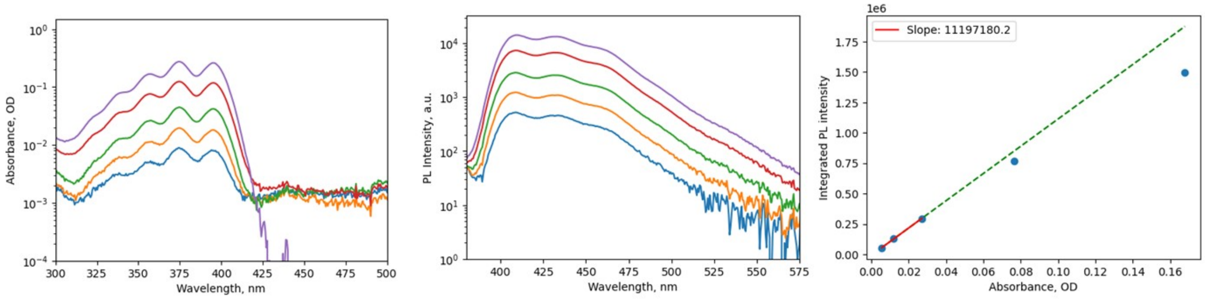

1o in mesitylene

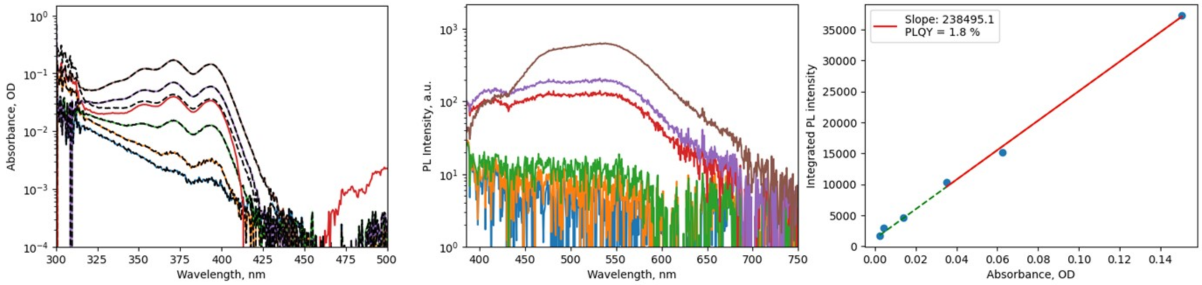

2o in mesitylene

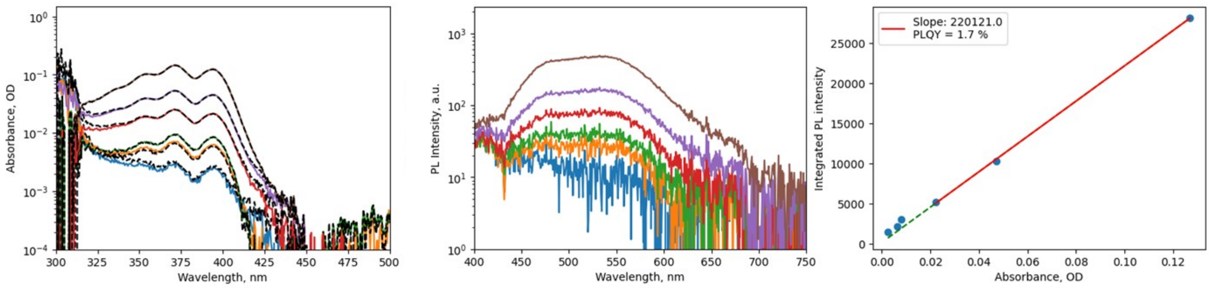

3o in mesitylene

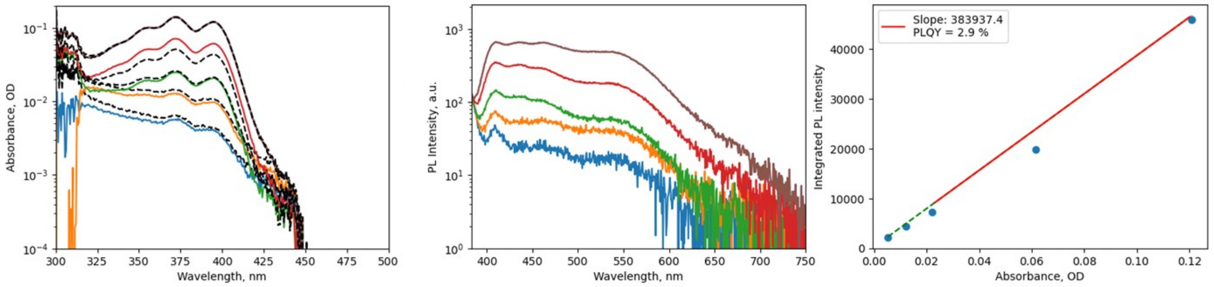

4o in mesitylene

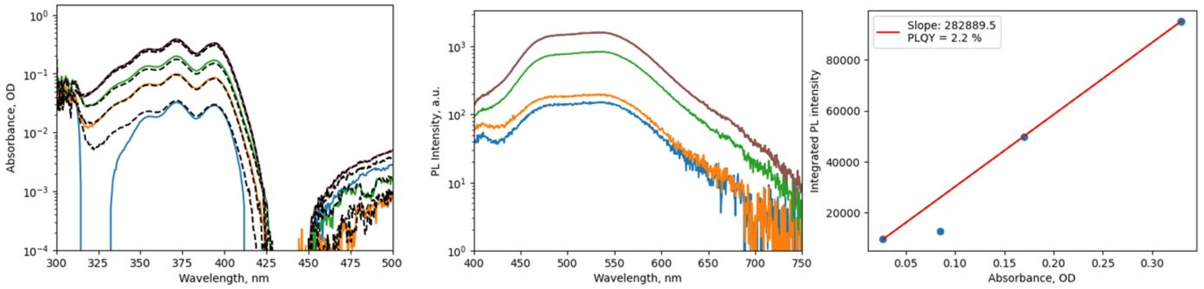

5o in mesitylene

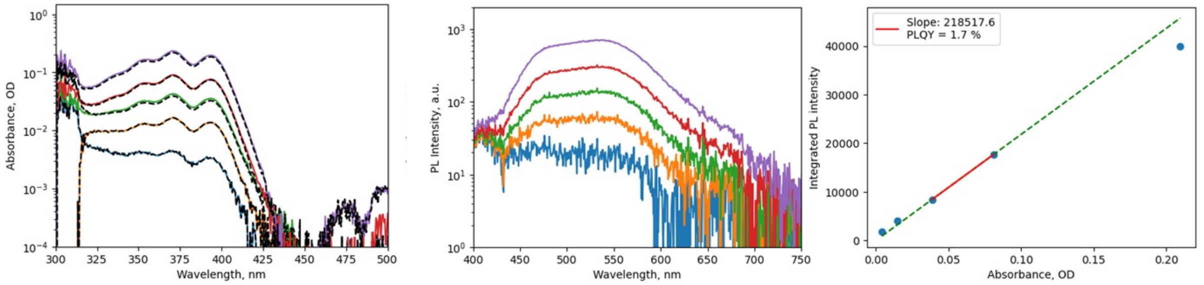

Figure S14: left column) absorption spectra of 9,10-diphenylanthracene in toluene and **1o** – **5o** in mesitylene at different concentrations. In the cases of **1o** – **5o**, dashed lines are used to indicate the UV-Vis spectra that were recorded after the emission spectra experiments to verify if any conversion was induced by the excitation at 365 nm; central column) emission spectra of 9,10-diphenylanthracene in toluene and **1o** – **5o** in mesitylene at different concentrations; right column) data fit for the determination of Φ_F.

Kinetic studies

3 × 10^{-5} M solutions of **1o** – **5o** in mesitylene were irradiated at 365 nm until full conversion to the corresponding **1c** – **5c**. Then, kinetics of the back-conversions were studied by monitoring the UV-Vis spectral change while heating the samples at temperatures of 130°C, 135°C, 140°C. The enthalpies and entropies of activation were finally calculated using the Eyring equation.

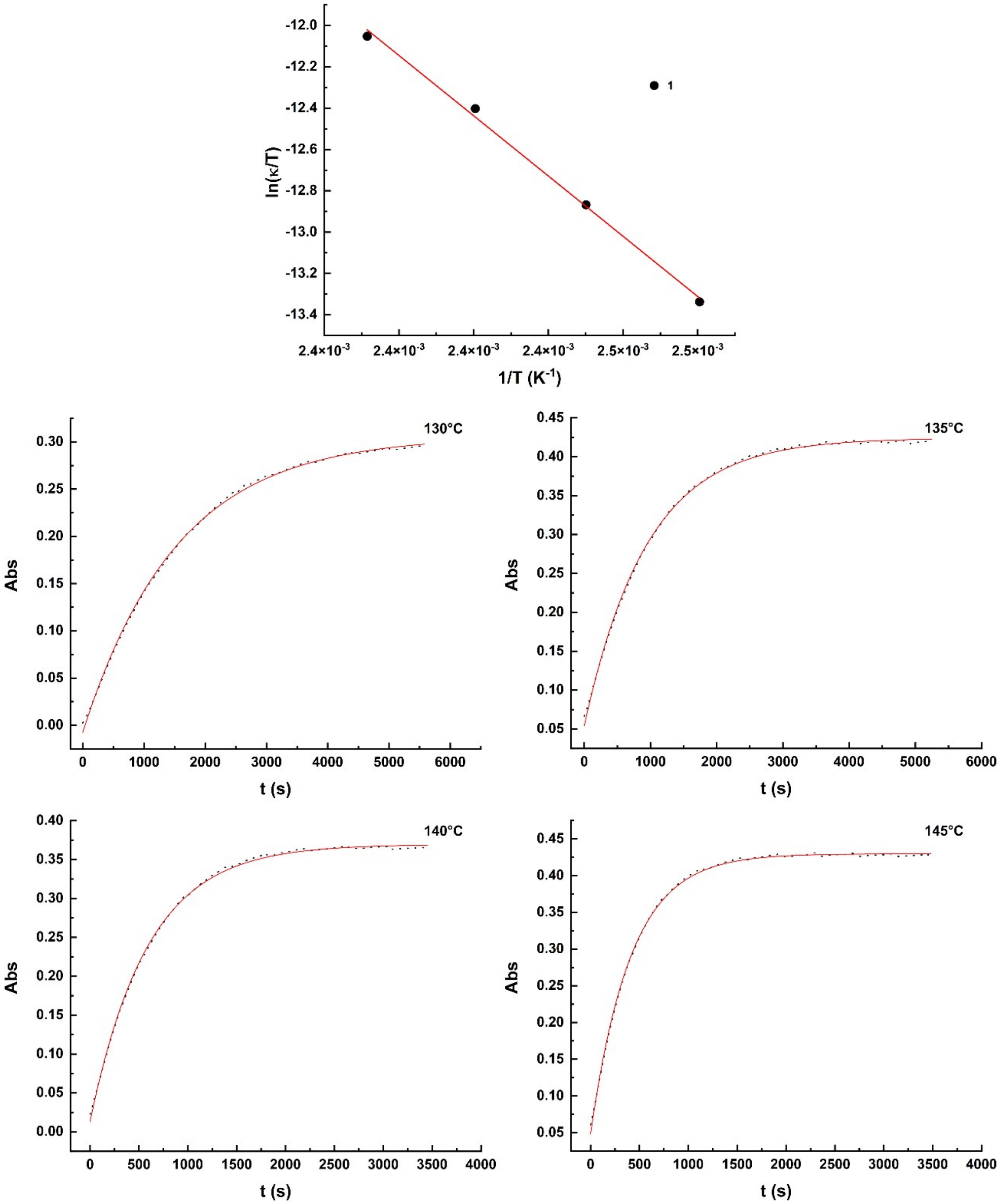

Figure S15: Kinetic study for the back-conversion **1c** ---> **1o**.

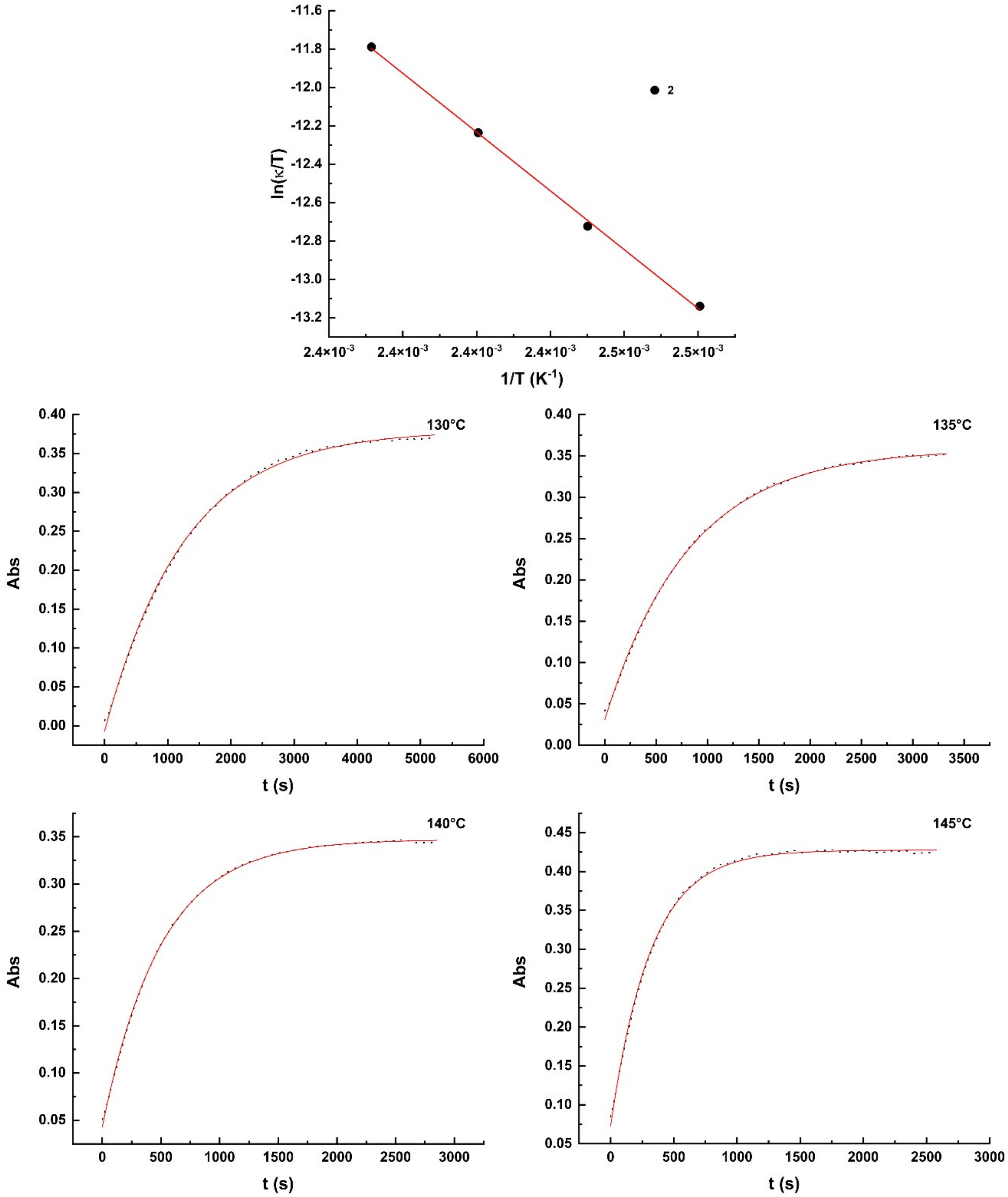

Figure S16: Kinetic study for the back-conversion **2c** ---> **2o**.

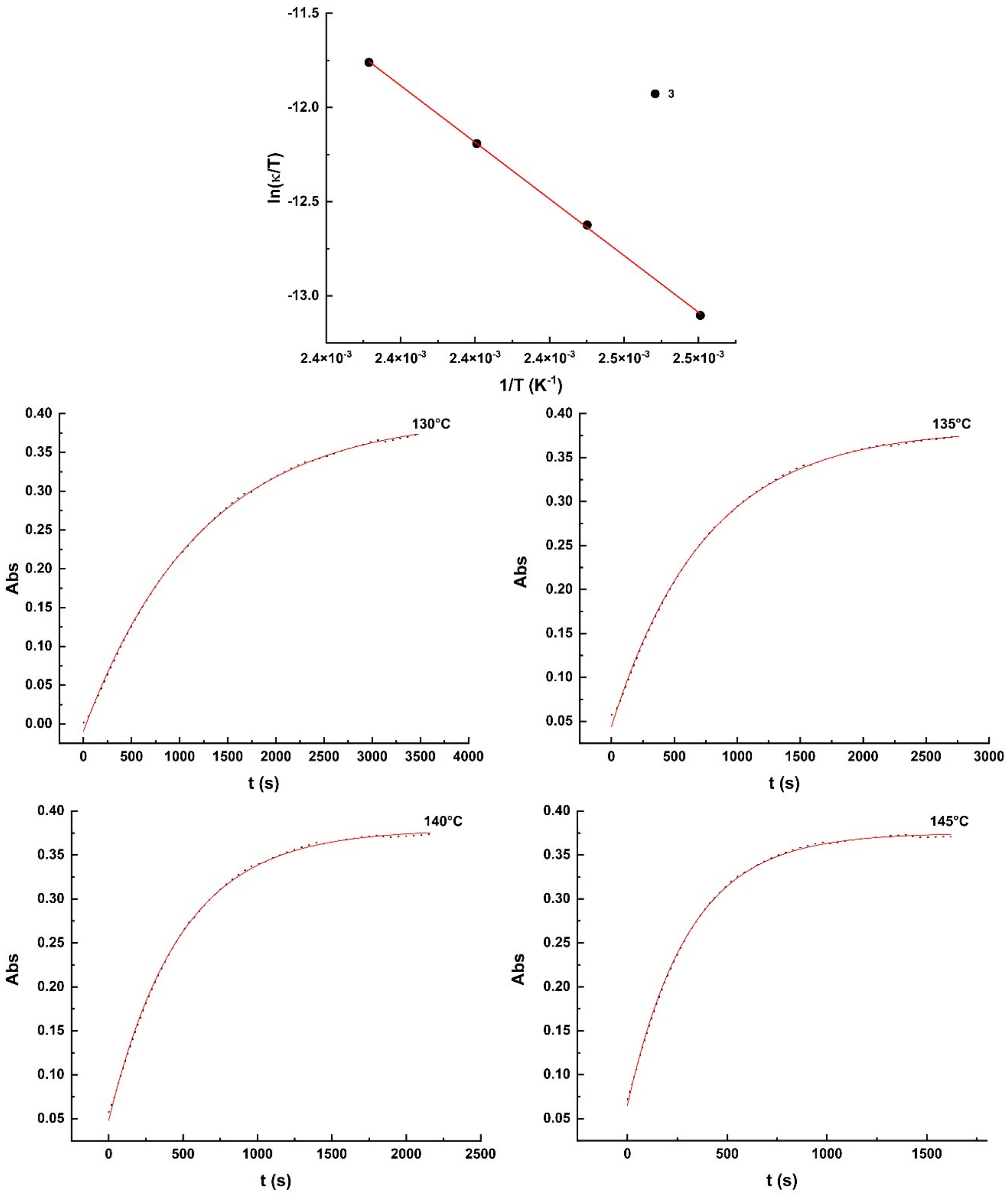

Figure S17: Kinetic study for the back-conversion **3c** ---> **3o**.

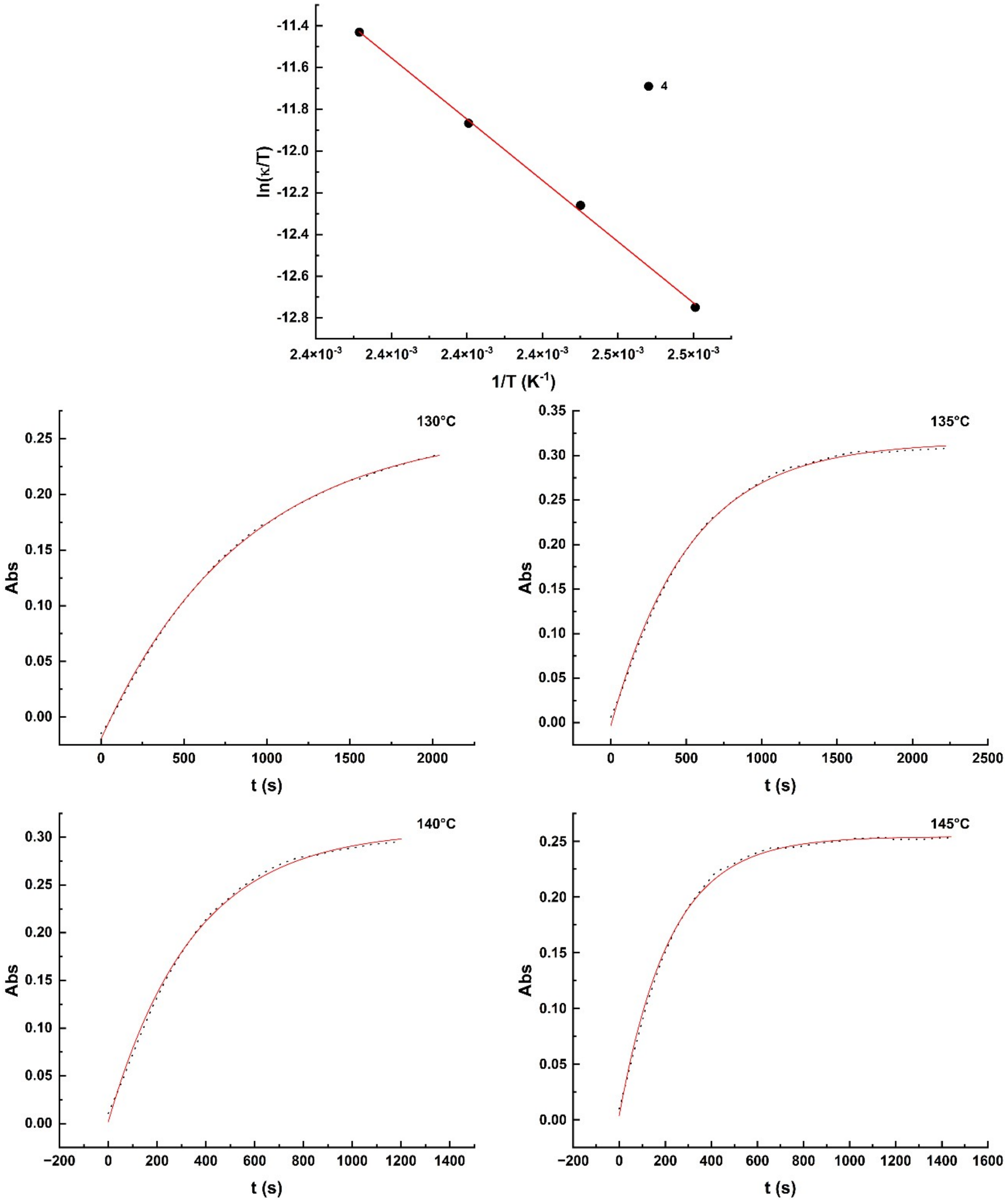

Figure S18: Kinetic study for the back-conversion **4c** ---> **4o**.

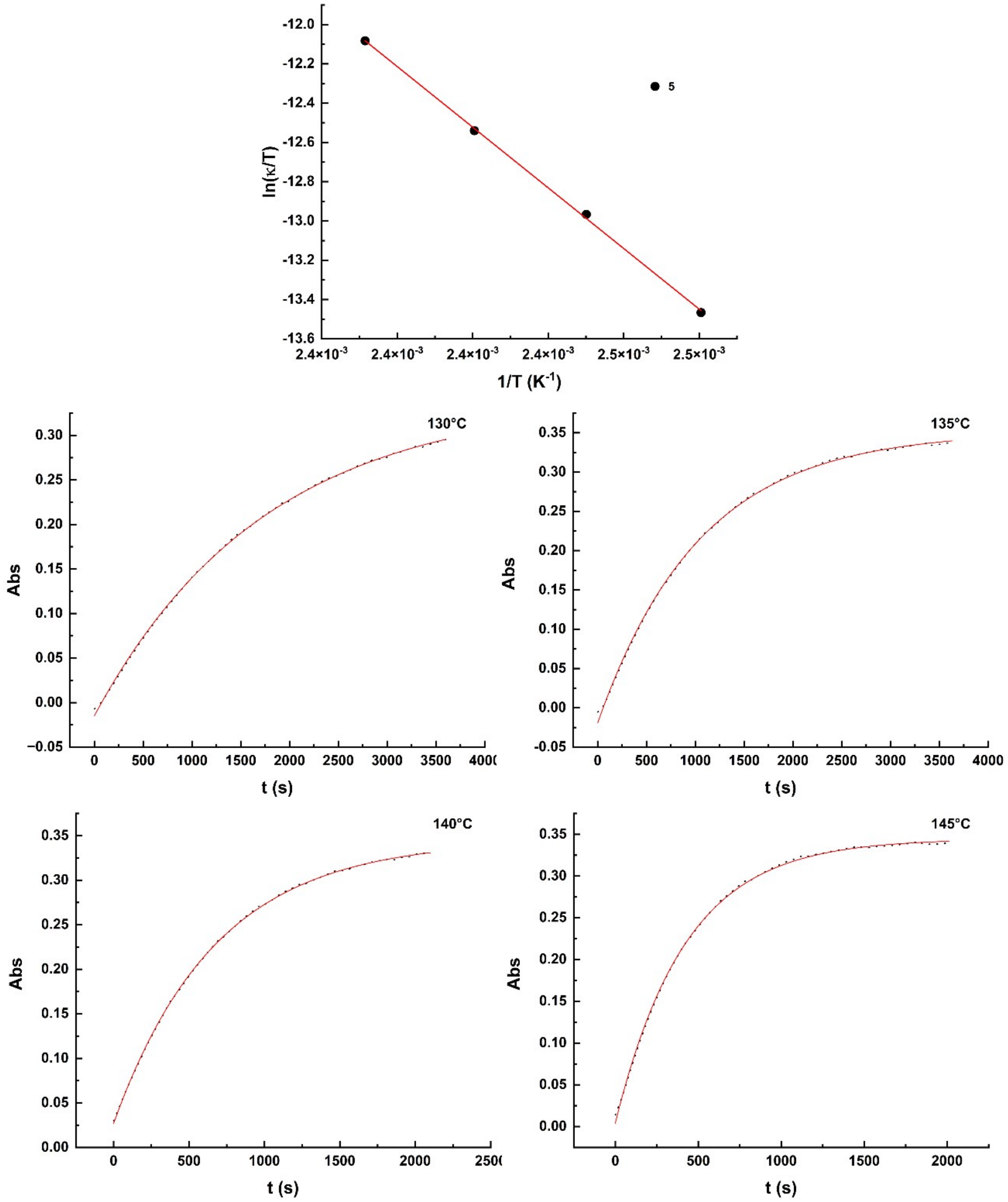

Figure S19: Kinetic study for the back-conversion **5c** ---> **5o**.

Solar energy storage efficiency

The solar energy storage efficiencies (η_{MOST}) for **1** – **5** were calculated using the following equation (eq. S2):

$$\eta_{\text{MOST}}\,(\%) = \frac{\int_{0}^{\lambda_{onset}} \frac{E_{\text{AM 1.5}}(\lambda)\cdot\phi_{o-c}\cdot\Delta G_{storage}}{h\nu}\cdot d\lambda}{\int E_{\text{AM 1.5}}(\lambda)\cdot d\lambda} \qquad (eq.\,S2)$$

Where $E_{AM\ 1.5}(\lambda)$ is the incoming solar power with an air mass (AM) of 1.5 in J s^{-1} m^{-2} nm^{-1}; Φo-c is the photoisomerization quantum yield for the forward reaction; $\Delta G_{storage}$ is the storage energy in J mol^{-1}; *h* is the Planck's constant in J s; ν is the frequency of incoming light in s^{-1} and N_A is Avogadro's constant in mol^{-1}.

Quantitative conversion of **1o** – **5o** to the corresponding **1c** – **5c** (and consequently no competitive absorption of the involved species between 300 nm and λ_{onset}) was assumed.

The solar spectrum with AM 1.5 is shown in Fig. S20, where the pink solid line at λ_{onset} = 425 nm and the coral pink area are used to show the portion of the spectrum absorbed by **1o** and **3o**. In the cases of **2o**, **4o** and **5o**, λ_{onset} was shifted to 426 nm, 424 nm and 426 nm, respectively.

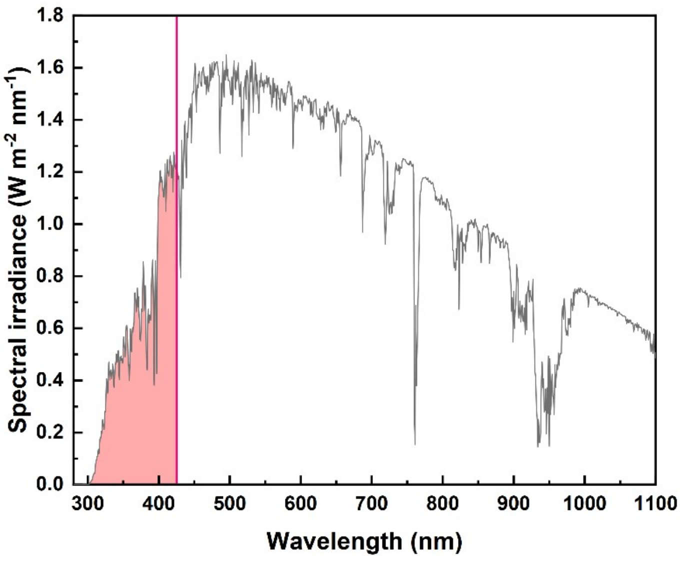

Figure S20: Solar power with an air mass (AM) of 1.5 (grey solid line), limit of absorption of **1o** and **3o** (pink solid line) and absorbed photons by **1o** and **3o** (coral pink area).

Theoretical modelling details

For each of the five compounds, simplified molecular-input line-entry system (SMILES) strings were generated using ChemDraw (version 22.0.0).[7] These SMILES strings were then employed to produce a diverse set of unique conformers for each compound using the RDKit Python package,[8] with a limit of 20 conformers per compound. The conformers were systematically generated by rotating all rotatable bonds. Subsequently, the geometries of the conformers were optimized using the semi-empirical tight-binding method GFN2-xTB.[9]

Following the conformer search, the geometry of the lowest-energy conformers was optimized in three steps: (i) using the r2SCAN-3c method[10] in combination with the cc-pVDZ basis set,[11] (ii) re-optimization using the cc-pVTZ basis set,[11] and (iii) optimization at the ωB97X-D3(BJ)[12]/cc-pVTZ level of theory. The ωB97X-D3(BJ) functional was chosen due to its proven accuracy in representing dispersion effects, which are crucial for proper depiction of intermolecular interactions.[13] A frequency analysis was performed after the final geometry optimization using ωB97X-D3(BJ)/cc-pVTZ.

Using the optimized minima for the open and closed states, the transition state of each system was obtained using the climbing image nudged elastic band (CI-NEB) method,[14] as implemented in ORCA (Release 5.0.1).[15] The CI-NEB calculations were executed using GFN2-xTB paired with the corresponding minimal basis set, followed by an optimization to a saddle point. The CI-NEB calculations were set to include 26 images and a pre-optimization. For the resulting transition state geometries, a frequency analysis was carried out using ωB97X-D3(BJ)/cc-pVTZ. For the optimized minima, the 40 lowest-lying excitations were calculated using the ωB97X-D3(BJ) functional in combination with the aug-cc-pVDZ basis set,[16] and spectra were generated *via* a Gaussian convolution of the vertical excitations with a full width at half maximum of 0.4 eV.

It should be noted that the reasoning for only performing a frequency analysis using the full density functional theory (DFT) method, rather than re-optimizing the GFN2-xTB transition state geometry, is due to the apparent robustness of the GFN2-xTB/CI-NEB approach. Initially, attempts to use the full DFT method resulted in faulty geometries, either resembling the minima of the open states or containing unwanted bonds between different parts of the two anthracene components. The difficulty in finding the transition states using the r2SCAN-3c and ωB97X-D3(BJ) functionals, as well as B3LYP,[17] CAM-B3LYP,[18] M06-2X,[19] and PBE0,[20-23] was consistent across both CI-NEB calculations and saddle point optimizations of the GFN2-xTB/CI-NEB maxima. However, the structure obtained using CI-NEB followed by a saddle point optimization both at the ωB97X-D3(BJ)/cc-pVTZ level of theory for system **1ts**, which successfully converged, exhibits a clear similarity in geometry and energy barrier compared to the GFN2-xTB/CI-NEB result. The barrier

obtained using the full DFT method is 310.88 kJ mol^{-1} and 247.53 kJ mol^{-1} for the back reaction barrier. This comparability justifies the use of the GFN2-xTB method for the CI-NEB calculations and the subsequent frequency analysis at the ωB97X-D3(BJ)/cc-pVTZ level of theory without further re-optimization.

Supplementary calculations were performed in implicit mesitylene using the conductor-like polarizable continuum model (C-PCM)[24,25] to mirror the experimental measurements. A relative permittivity of 2.2660[26] and a refractive index of 2.2482[27] were used. The ωB97X-D3(BJ)/cc-pVTZ minima were re-optimized using the C-PCM method, and a frequency analysis of the GFN2-xTB/CI-NEB maxima was performed to include the solvent. Note that the GFN2-xTB method is not compatible with the C-PCM method in ORCA; thus, the vacuum CI-NEB geometry was used for the modified frequency analysis. The re-optimized minima were subsequently used to calculate the 40 lowest-lying vertical excitations in mesitylene using the ωB97X-D3(BJ)/aug-cc-pVDZ level of theory.

The theoretically predicted energy storage densities were obtained *via* (eq. S3):

$$\rho_{\text{storage}}\left(MJ\ kg^{-1}\right) = \frac{\Delta G_{\text{storage}}\left(kJ\ mol^{-1}\right)}{m\left(g\ mol^{-1}\right)} \qquad (eq. S3)$$

Where ρ_{storage} is the energy storage density, $\Delta G_{\text{storage}}$ is the Gibbs free energy storage (given by the difference in energy between the open and closed minima), and *m* is the molecular mass of the given system. The following values were used for the molecular mass of the five systems, given in units of g mol^{-1}: **1** (430.55), **2** (460.58), **3** (490.6), **4** (458.6), and **5** (466.53).

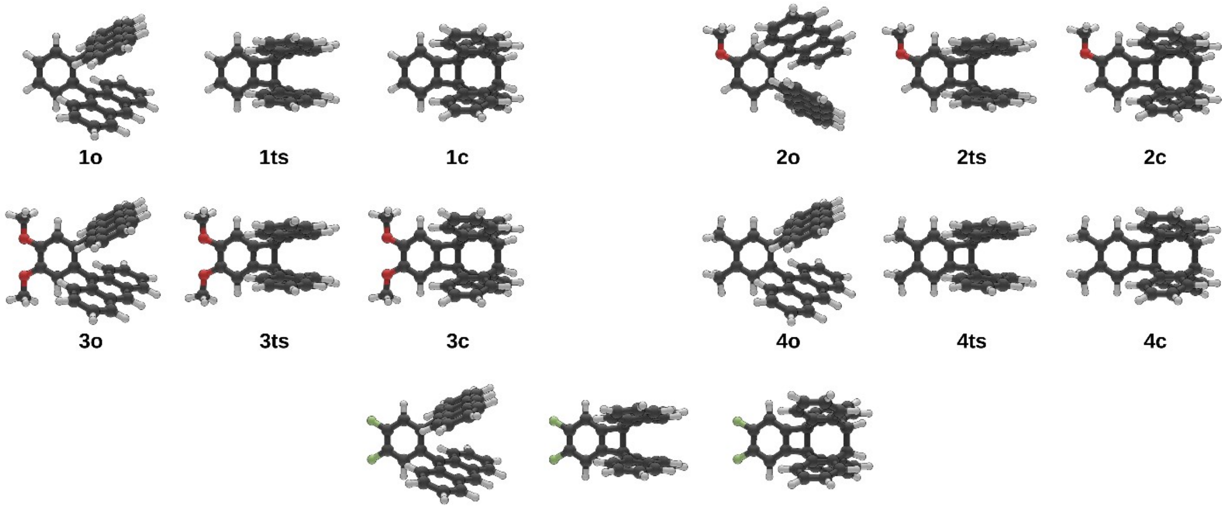

1o **1ts** **1c** **2o** **2ts** **2c**

3o **3ts** **3c** **4o** **4ts** **4c**

5o **5ts** **5c**

Figure S21: Depictions of the computed minima, transition state, and maxima geometries for the five considered systems.

Table S1: Theoretical reaction and back-reaction barriers for the five photoswitches in vacuum and *implicit mesitylene*. All values were obtained at the ωB97X-D3(BJ)/cc-pVTZ level of theory.

	Reaction barrier (kJ mol^{-1})	Back-reaction barrier (kJ mol^{-1})
**1o/1c**	304.4, *305.5*	241.0, *243.1*
**2o/2c**	308.7, *308.0*	241.8, *244.3*
**3o/3c**	310.0, *314.8*	241.3, *247.0*
**4o/4c**	312.7, *314.1*	245.0, *248.1*
**5o/5c**	305.6, *305.4*	241.3, *242.0*

Table S2: Theoretical C9-C9′ and C10-C10′ atomic distances in the CI-NEB transition state structures.

	C9-C9′ (Å)	C10-C10′ (Å)
**1ts**	1.70	3.02
**2ts**	1.70	3.00
**3ts**	1.70	3.00
**4ts**	1.71	3.00
**5ts**	1.70	3.00

Crystal structure details

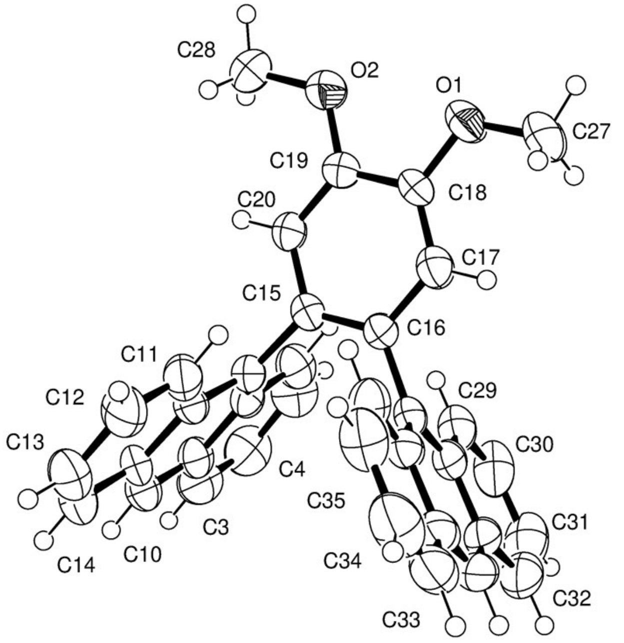

Figure S22: ORTEP drawing of **3o**. CCDC 2359712 contains the supplementary crystallographic data for this crystal structure.

Table S3: Crystal data and structure refinement for **3o**.

Identification code	Km1b
Empirical formula	C36 H26 O2
Formula weight	490.57
Temperature	294(2) K
Wavelength	0.71073 Å
Crystal system	Triclinic
Space group	P -1
Unit cell dimensions	a = 8.927(5) Å α = 77.669(13)°.<br>b = 12.154(7) Å β = 84.626(14)°.<br>c = 12.340(7) Å γ = 74.586(13)°.
Volume	1259.9(13) $Å^3$
Z	2
Density (calculated)	1.293 Mg/m^3
Absorption coefficient	0.079 mm^{-1}
F(000)	516
Crystal size	0.370 × 0.100 × 0.090 mm^3
Theta range for data collection	1.690 to 28.135°
Index ranges	-11<=h<=11, -16<=k<=16, -16<=l<=16
Reflections collected	35811
Independent reflections	6084
Completeness to theta = 25.242°	100%
Absorption correction	Semi-empirical from equivalents
Max. and min. transmission	1 and 0.78
Refinement method	Full-matrix least-squares on F^2
Data / restraints / parameters	6084 / 0 / 346
Goodness-of-fit on F^2	0.954
Final R indices [I>2sigma(I)]	R1 = 0.0868, wR2 = 0.1544
R indices (all data)	R1 = 0.2782, wR2 = 0.2272
Extinction coefficient	0.014(2)
Largest diff. peak and hole	0.194 and -0.205 e.$Å^{-3}$

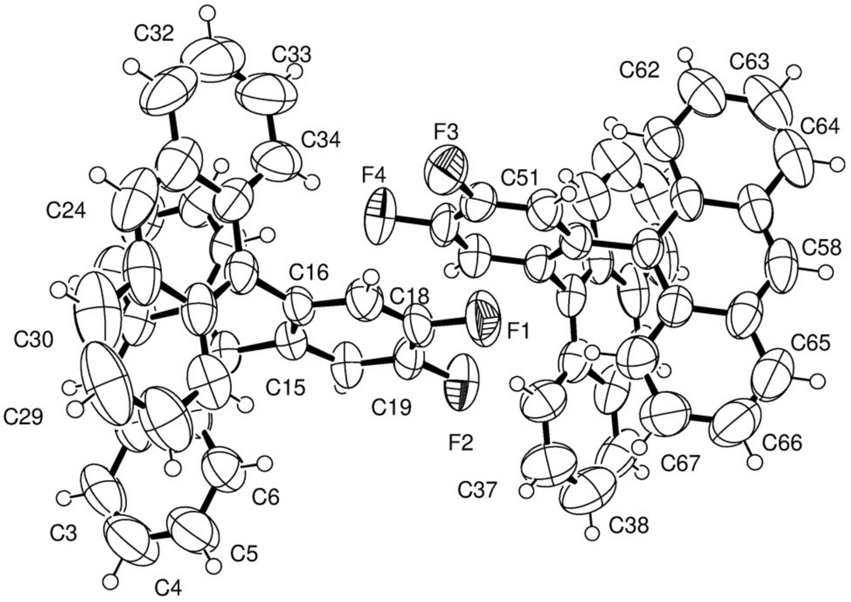

Figure S23: ORTEP drawing of **5o**. CCDC 2359711 contains the supplementary crystallographic data for this crystal structure.

Table S4: Crystal data and structure refinement for **5o**.

Identification code	Km3b
Empirical formula	C36 H20 F2
Formula weight	466.50
Temperature	295(2) K
Wavelength	0.71073 Å
Crystal system	Triclinic
Space group	P -1
Unit cell dimensions	a = 12.8757(14) Å α = 115.734(2)°.<br>b = 14.4236(15) Å β = 106.734(2)°.<br>c = 15.2937(16) Å γ = 90.696(2)°.
Volume	2419.0(4) $Å^3$
Z	4
Density (calculated)	1.281 Mg/m^3
Absorption coefficient	0.084 mm^{-1}
F(000)	968
Crystal size	0.280 × 0.220 × 0.200 mm^3
Theta range for data collection	1.563 to 28.296°
Index ranges	-17<=h<=17, -19<=k<=19, -20<=l<=20
Reflections collected	74056
Independent reflections	11991 [R(int) = 0.0607]
Completeness to theta = 25.242°	100%
Absorption correction	Semi-empirical from equivalents
Max. and min. transmission	0.7457 and 0.6860
Refinement method	Full-matrix least-squares on F^2
Data / restraints / parameters	11991 / 0 / 650

Goodness-of-fit on F^2	1.015
Final R indices [I>2sigma(I)]	R1 = 0.0626, wR2 = 0.1372
R indices (all data)	R1 = 0.1456, wR2 = 0.1806
Extinction coefficient	0.0013(4)
Largest diff. peak and hole	0.182 and -0.181 e.Å^{-3}

^{1}H- ,^{13}C- and ^{19}F-NMR spectra

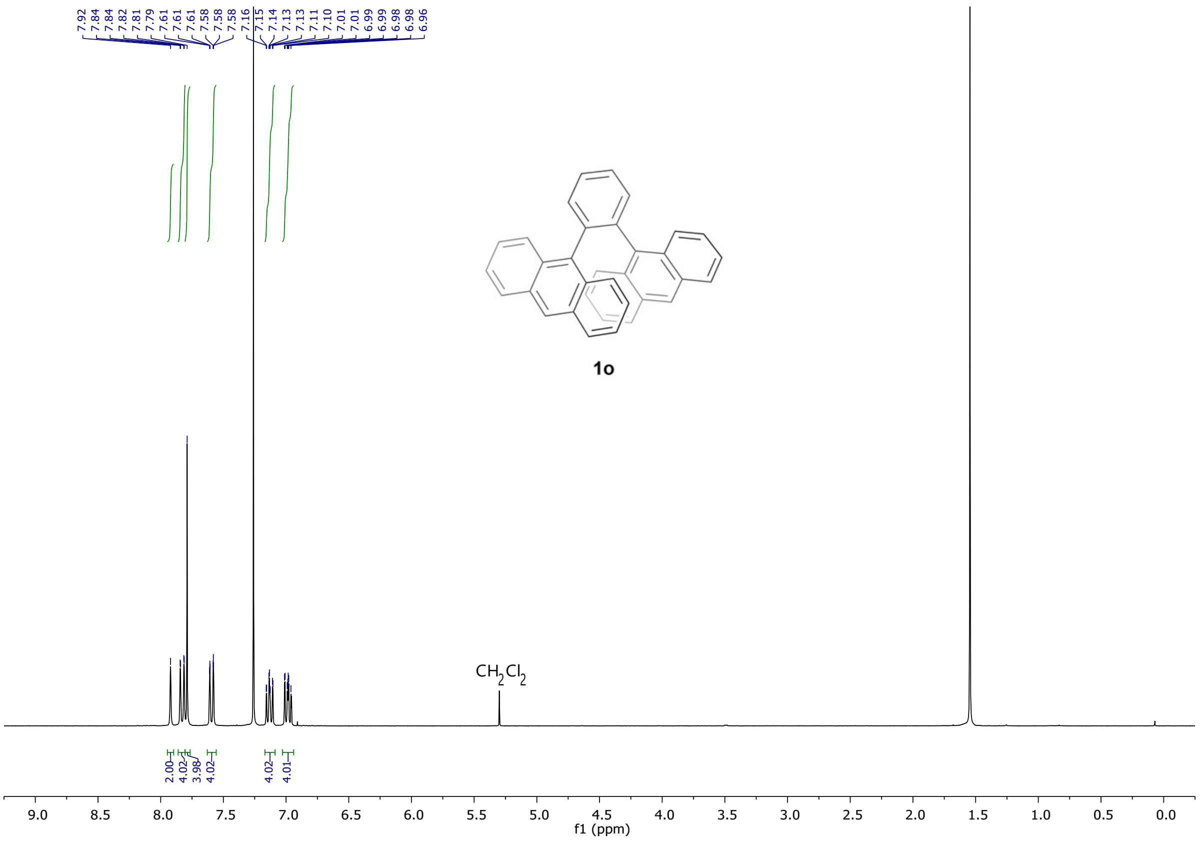

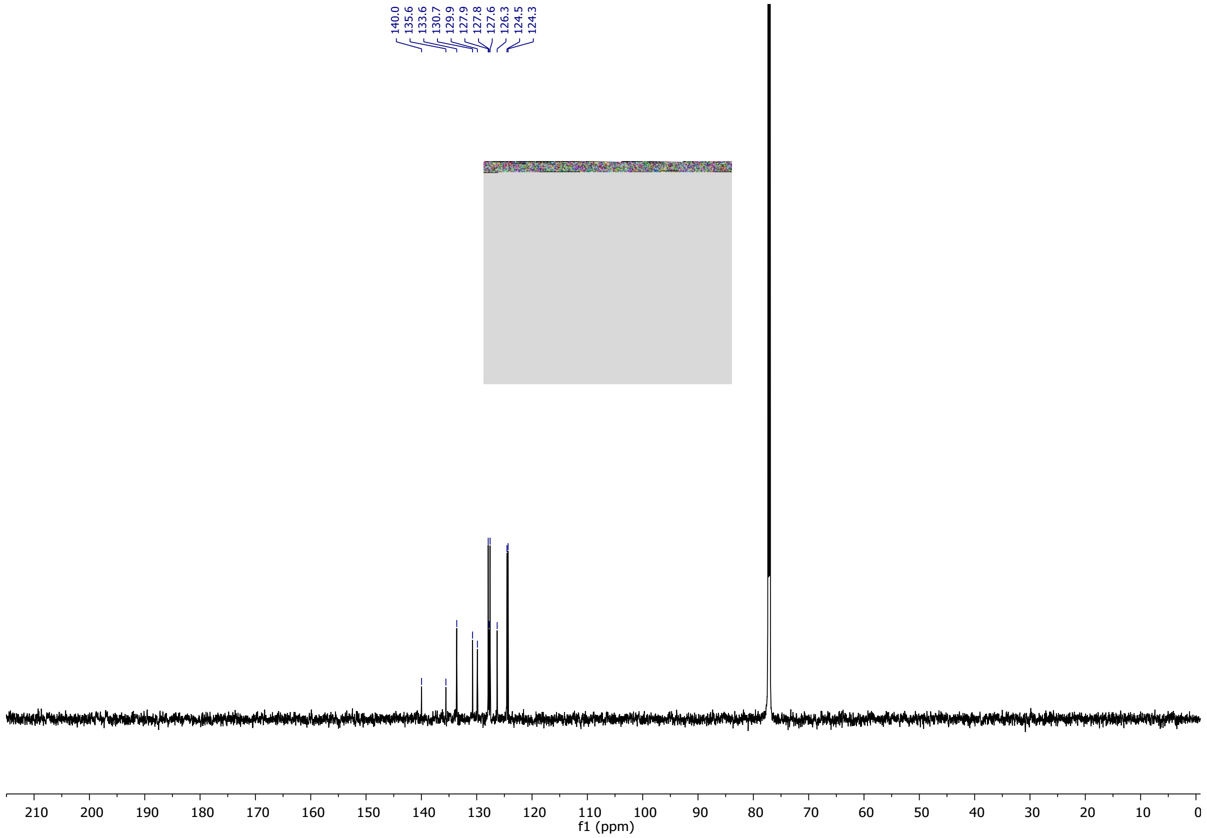
140.0
135.6
133.6
130.7
129.9
127.9
127.8
127.6
126.3
124.5
124.3
210 200 190 180 170 160 150 140 130 120 110 100 90 80 70 60 50 40 30 20 10 0
f1 (ppm)

COSY

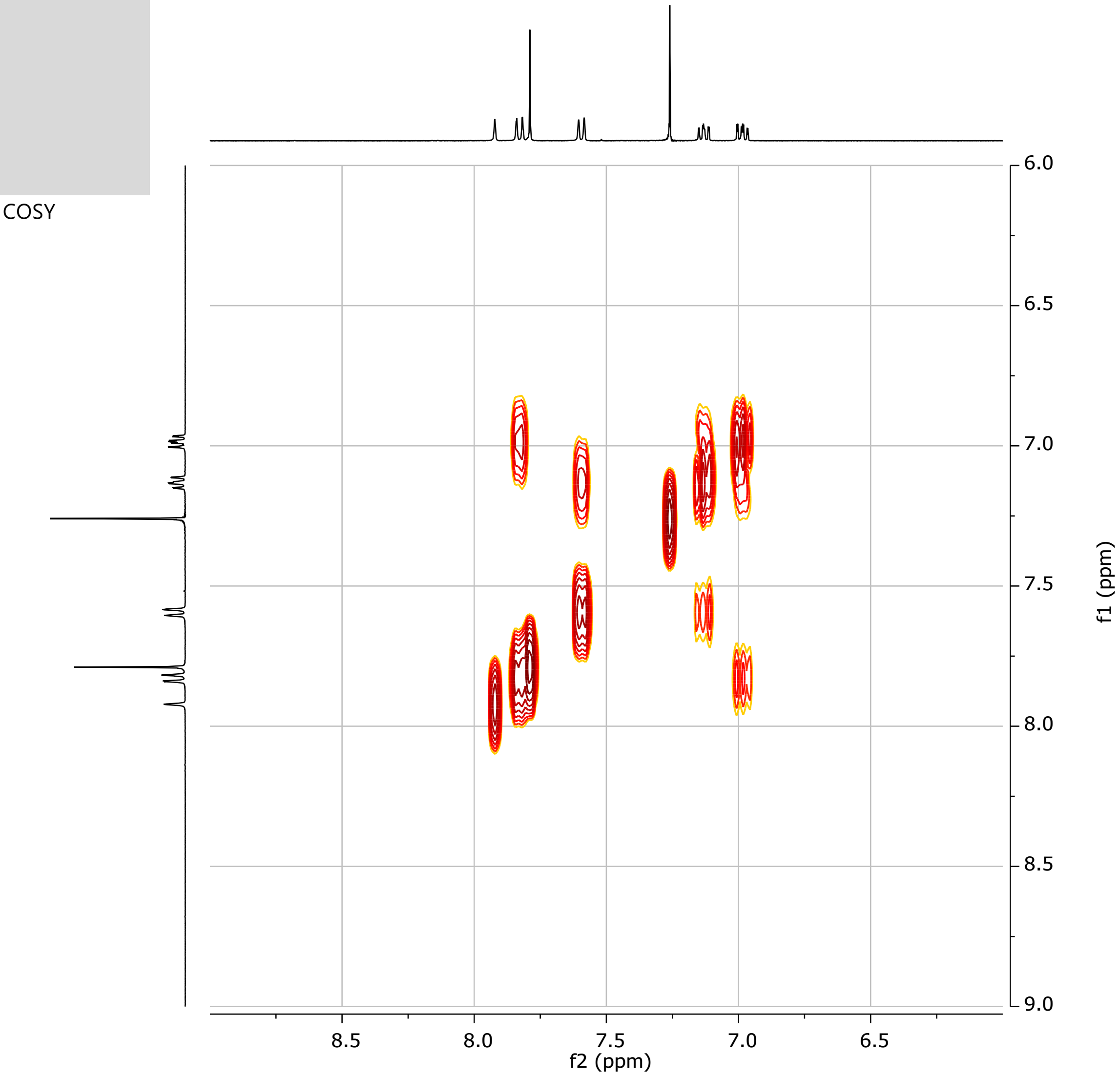

HSQC

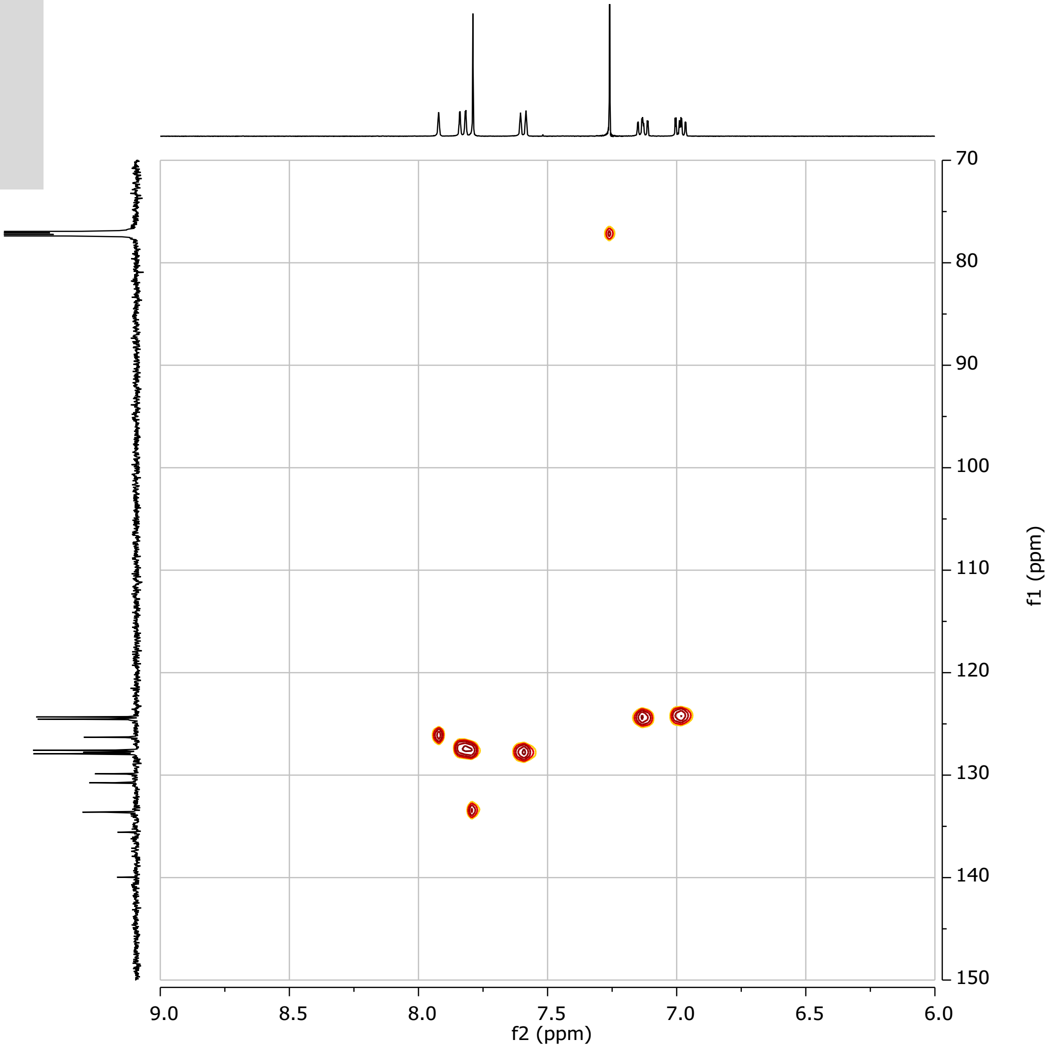

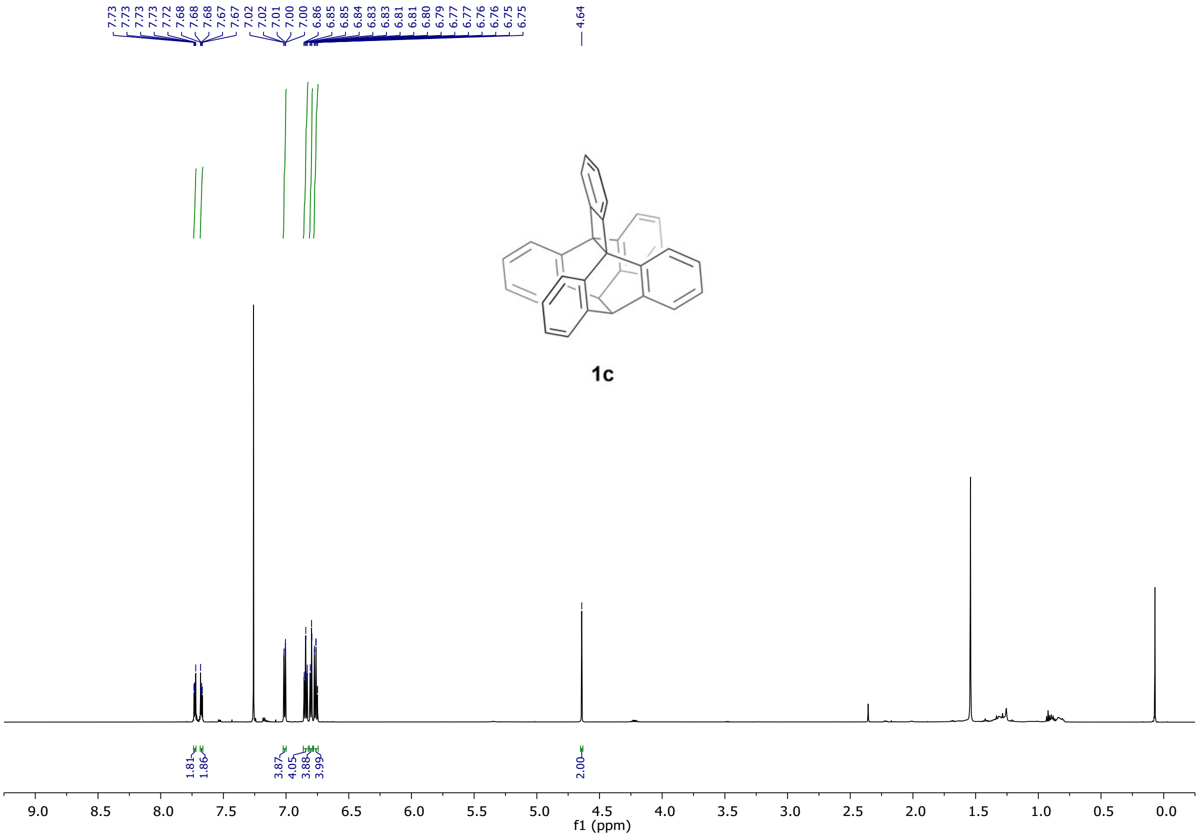

7.73
7.73
7.73
7.73
7.72
7.68
7.68
7.68
7.67
7.67
7.02
7.02
7.01
7.00
7.00
6.86
6.85
6.85
6.84
6.83
6.83
6.81
6.81
6.80
6.79
6.77
6.77
6.76
6.75
6.75
4.64
1.81
1.86
3.87
4.05
3.88
3.99
2.00
9.0 8.5 8.0 7.5 7.0 6.5 6.0 5.5 5.0 4.5 4.0 3.5 3.0 2.5 2.0 1.5 1.0 0.5 0.0
f1 (ppm)

1c

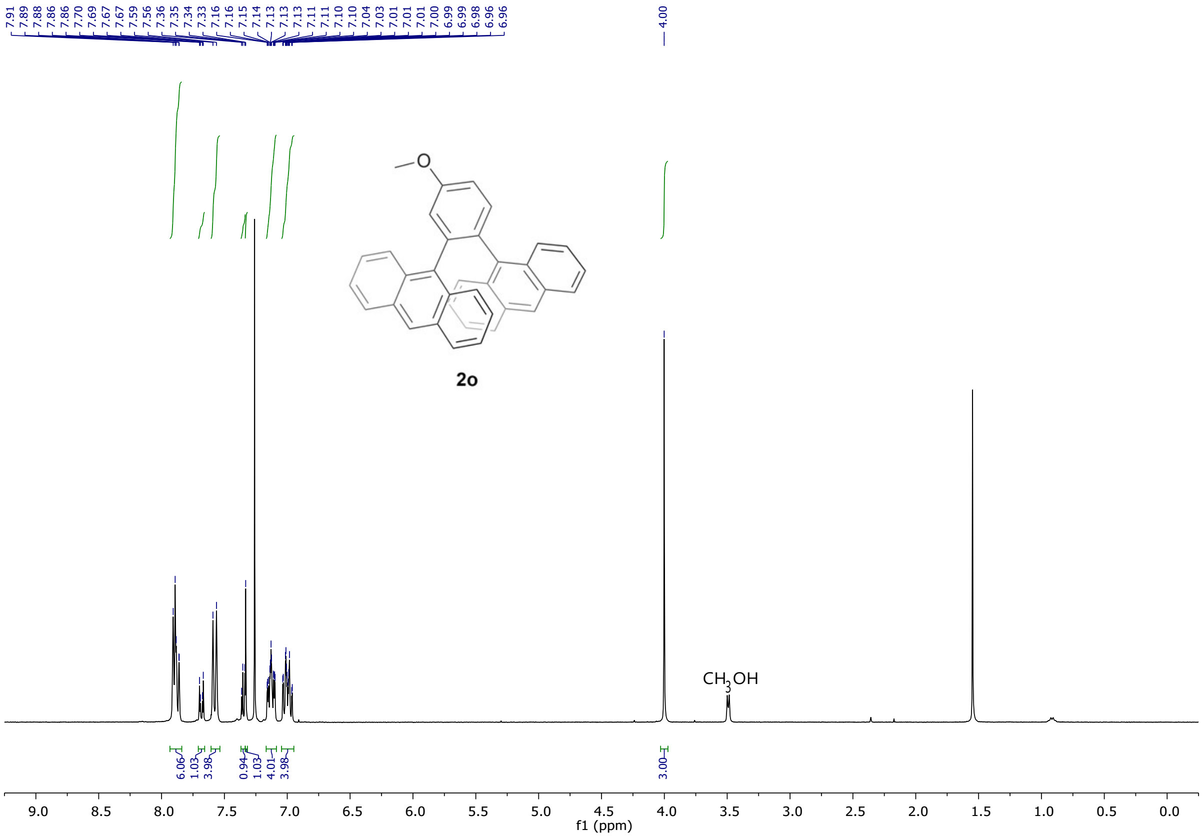
7.91 7.89 7.88 7.86 7.86 7.70 7.69 7.67 7.67 7.59 7.56 7.36 7.35 7.34 7.33 7.16 7.15 7.14 7.13 7.13 7.13 7.11 7.11 7.10 7.04 7.03 7.01 7.01 7.01 7.00 6.99 6.99 6.98 6.96 6.96
4.00
2o
CH3OH
6.06 1.03 3.98 0.94 1.03 4.01 3.98
3.00
9.0 8.5 8.0 7.5 7.0 6.5 6.0 5.5 5.0 4.5 4.0 3.5 3.0 2.5 2.0 1.5 1.0 0.5 0.0
f1 (ppm)

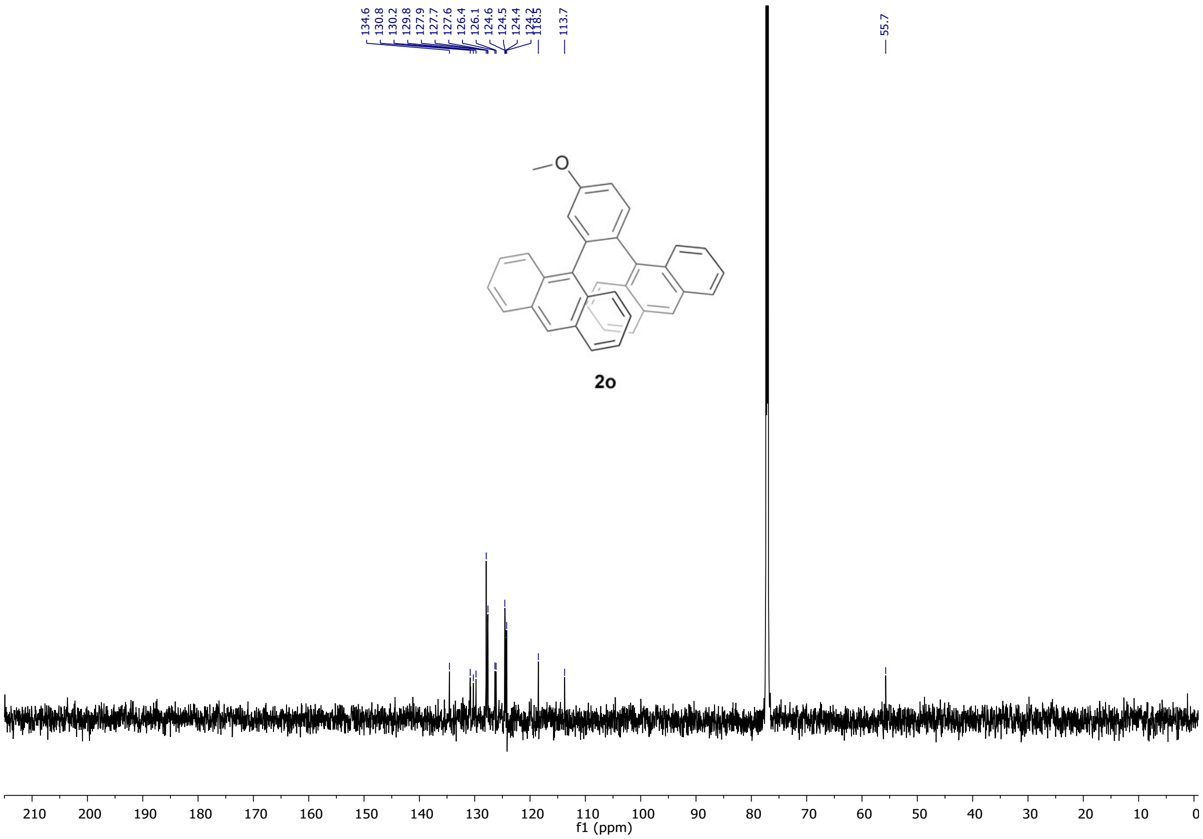
134.6 130.8 130.2 129.8 127.9 127.7 127.6 126.4 126.1 124.6 124.5 124.4 124.2 118.5
113.7
55.7
2o
210 200 190 180 170 160 150 140 130 120 110 100 90 80 70 60 50 40 30 20 10 0
f1 (ppm)

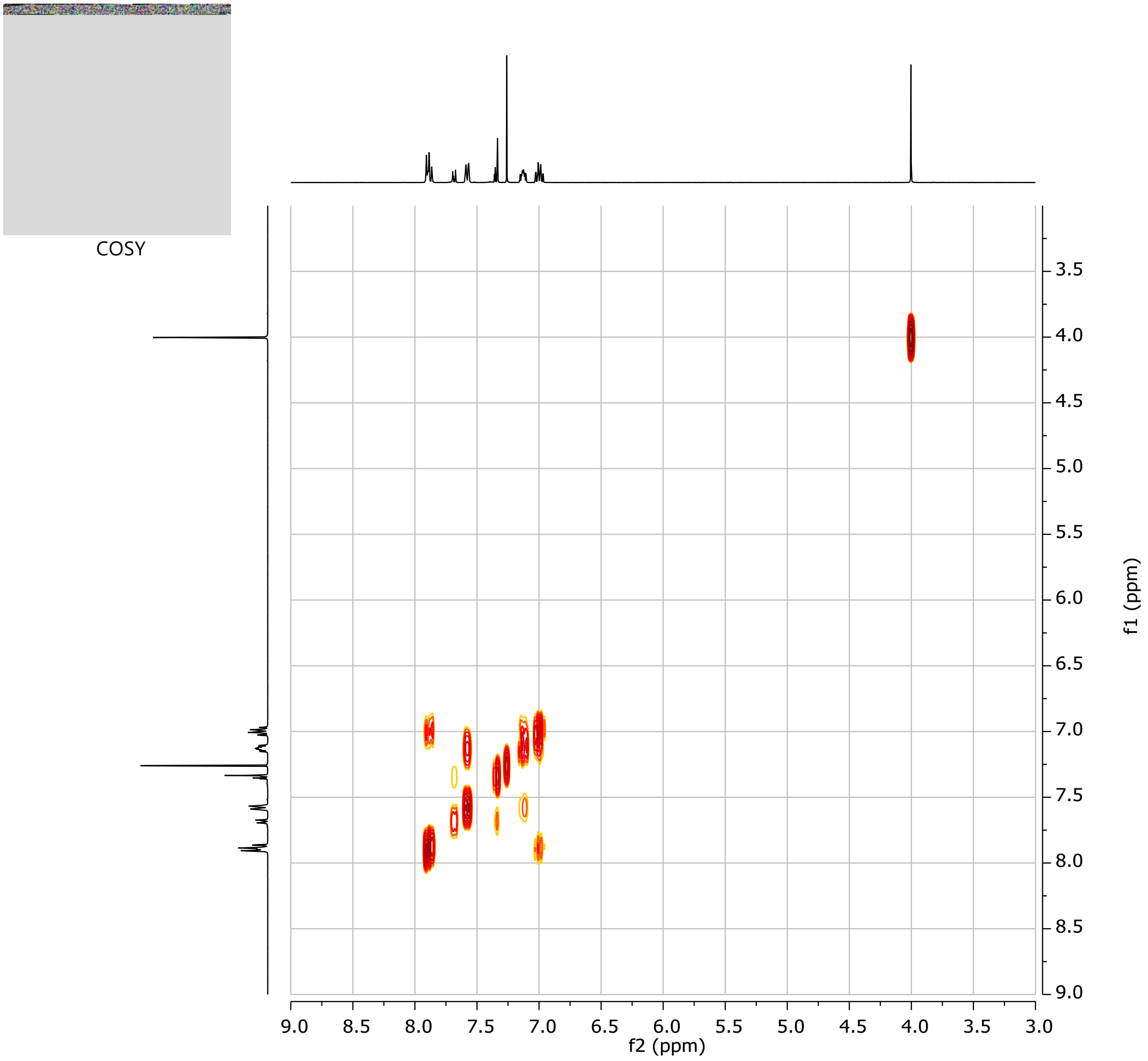

COSY
f1 (ppm)
f2 (ppm)

HSQC

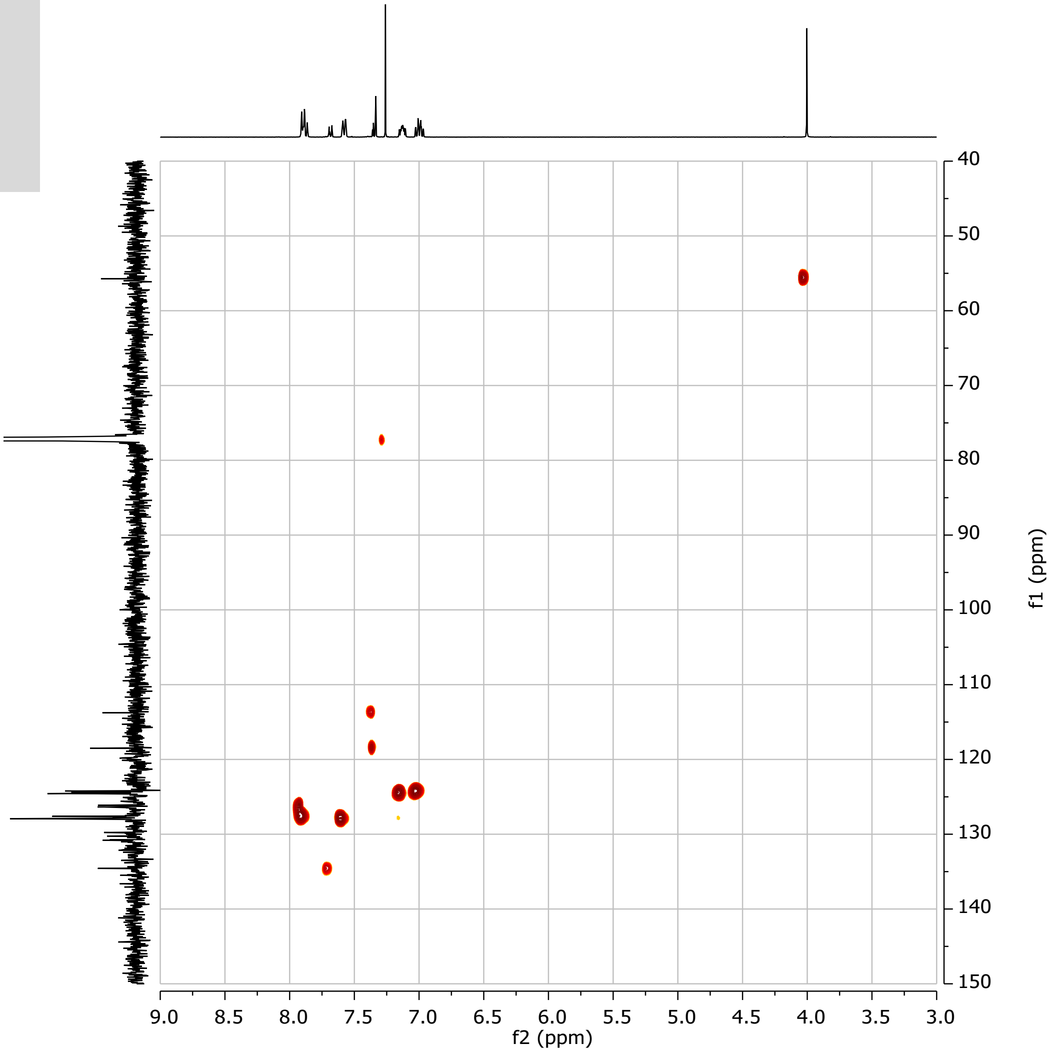
9.0 8.5 8.0 7.5 7.0 6.5 6.0 5.5 5.0 4.5 4.0 3.5 3.0
f2 (ppm)
40 50 60 70 80 90 100 110 120 130 140 150
f1 (ppm)

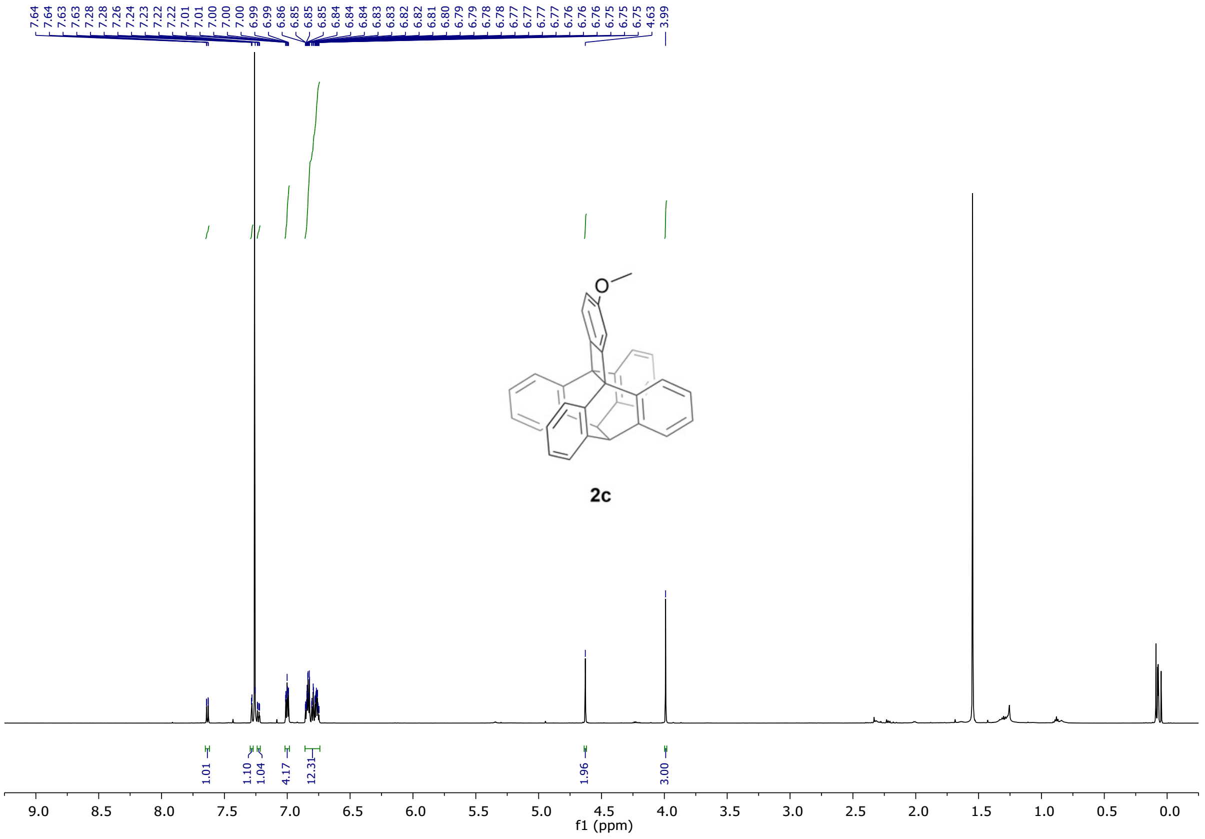
7.64 7.64 7.63 7.28 7.28 7.26 7.24 7.23 7.22 7.22 7.01 7.01 7.00 7.00 7.00 6.99 6.99 6.86 6.85 6.85 6.85 6.84 6.84 6.84 6.83 6.83 6.82 6.81 6.80 6.79 6.79 6.78 6.77 6.77 6.77 6.77 6.76 6.76 6.76 6.75 6.75 4.63 3.99
O
2c
1.01
1.10
1.04
4.17
12.31
1.96
3.00
9.0 8.5 8.0 7.5 7.0 6.5 6.0 5.5 5.0 4.5 4.0 3.5 3.0 2.5 2.0 1.5 1.0 0.5 0.0
f1 (ppm)

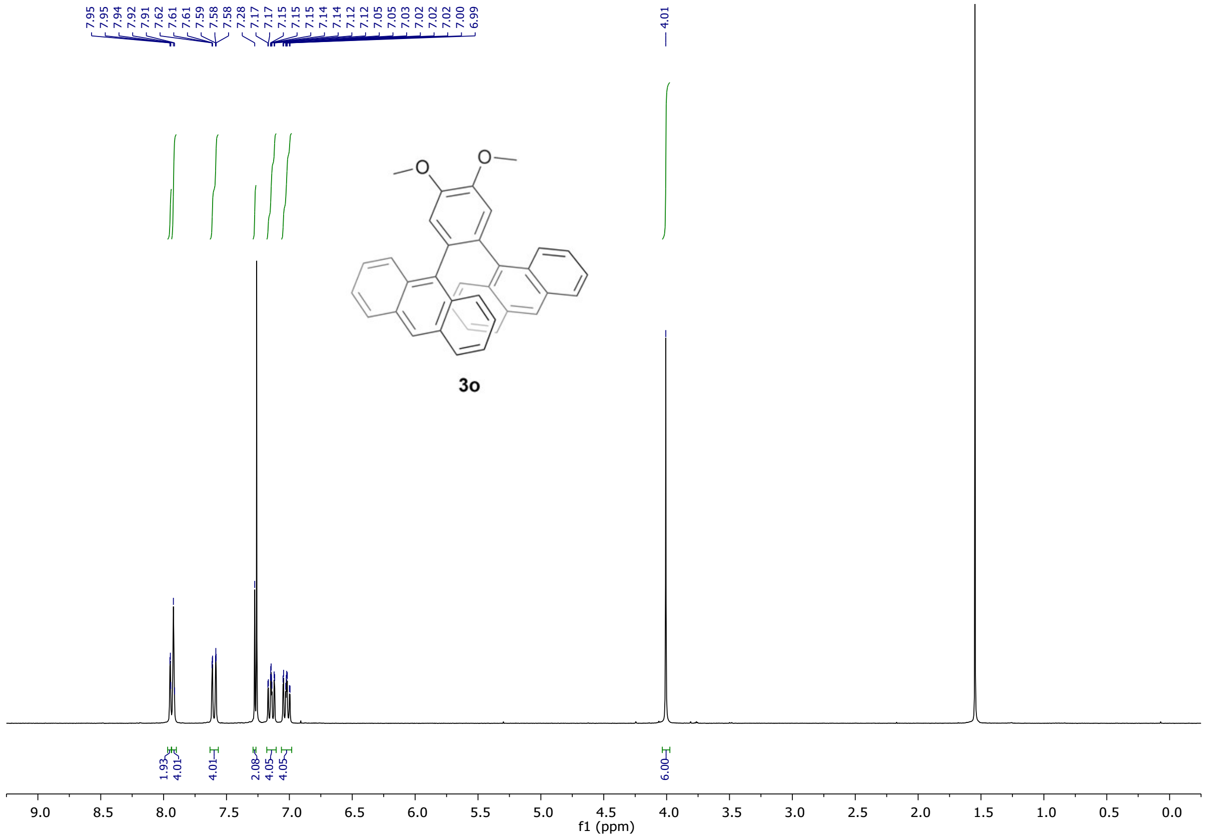
7.95 7.95 7.94 7.92 7.91 7.62 7.61 7.61 7.59 7.58 7.58 7.28 7.17 7.17 7.15 7.15 7.15 7.14 7.14 7.12 7.12 7.05 7.05 7.03 7.02 7.02 7.02 7.02 7.00 6.99
4.01
O
O
3o
1.93
4.01
4.01
2.08
4.05
4.05
6.00
9.0 8.5 8.0 7.5 7.0 6.5 6.0 5.5 5.0 4.5 4.0 3.5 3.0 2.5 2.0 1.5 1.0 0.5 0.0
f1 (ppm)

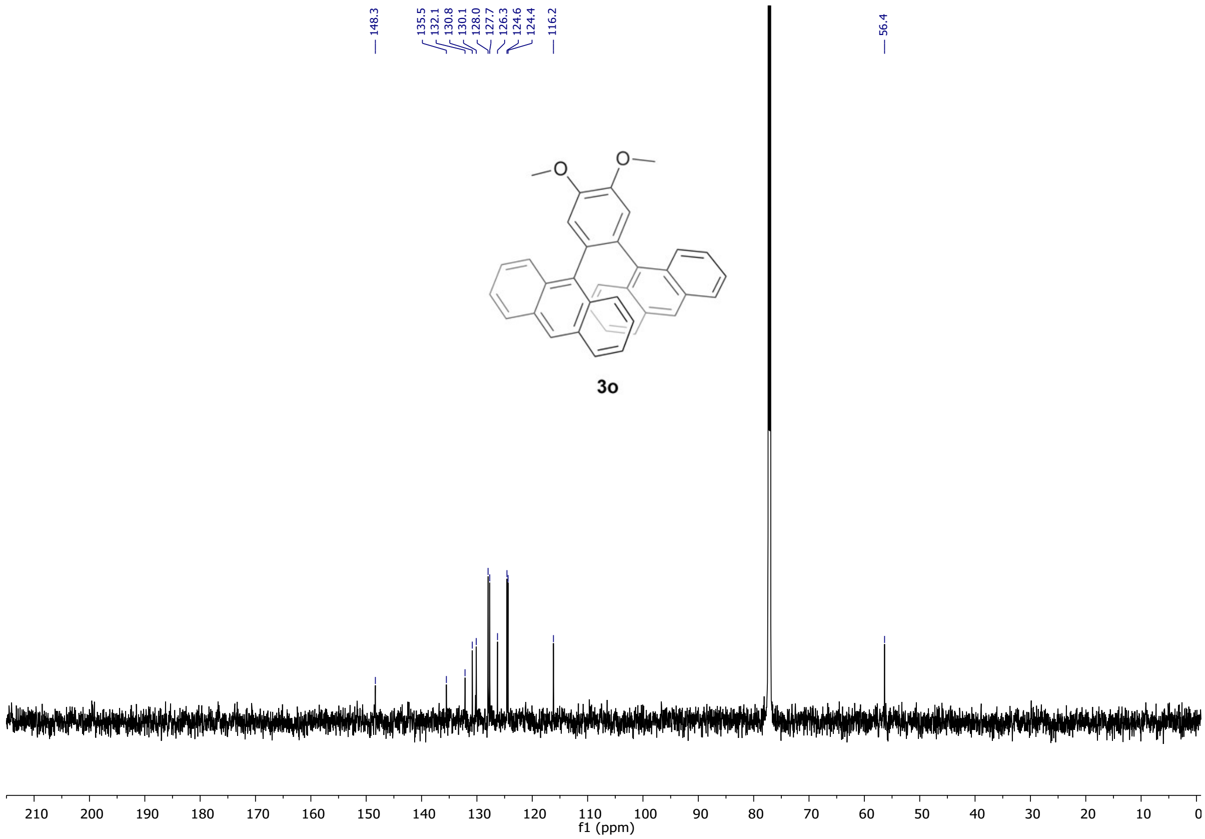
148.3
135.5 132.1 130.8 130.1 128.0 127.7 126.3 124.6 124.4
116.2
56.4
O
O
3o
210 200 190 180 170 160 150 140 130 120 110 100 90 80 70 60 50 40 30 20 10 0
f1 (ppm)

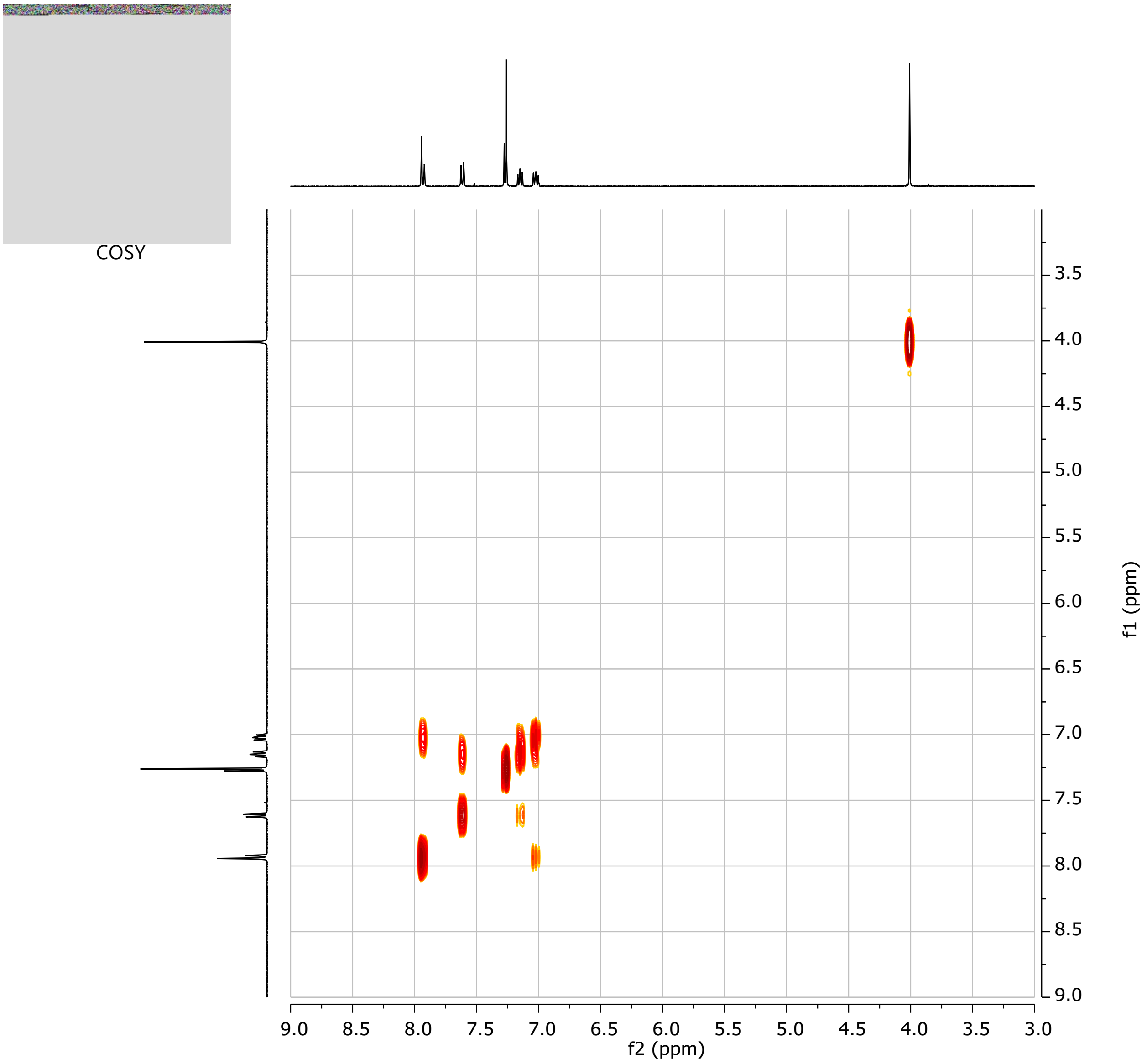
COSY
f1 (ppm)
f2 (ppm)
3.5
4.0
4.5
5.0
5.5
6.0
6.5
7.0
7.5
8.0
8.5
9.0
9.0 8.5 8.0 7.5 7.0 6.5 6.0 5.5 5.0 4.5 4.0 3.5 3.0

HSQC

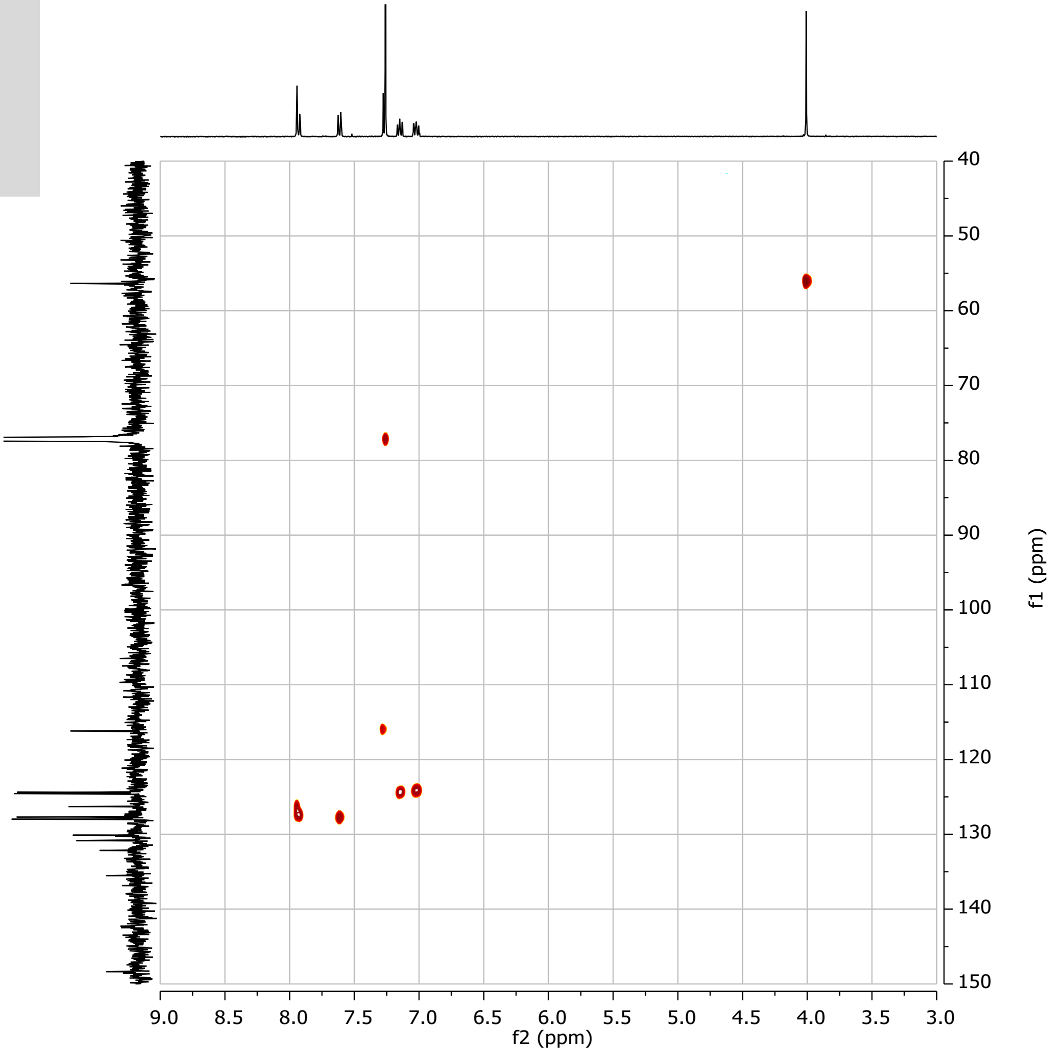

9.0
8.5
8.0
7.5
7.0
6.5
6.0
5.5
5.0
4.5
4.0
3.5
3.0
f2 (ppm)
40
50
60
70
80
90
100
110
120
130
140
150
f1 (ppm)

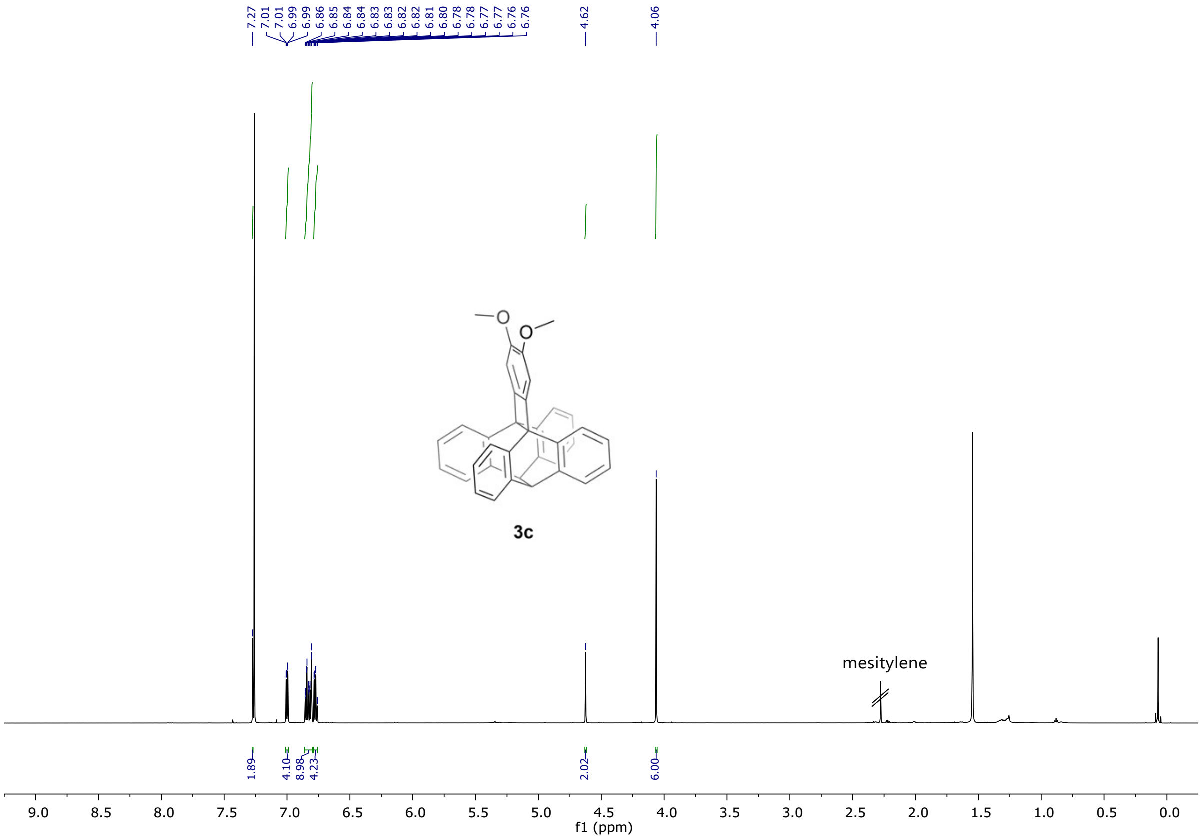

7.27
7.01
7.01
6.99
6.99
6.86
6.85
6.84
6.84
6.83
6.83
6.82
6.82
6.81
6.80
6.78
6.78
6.77
6.76
6.76
4.62
4.06
O
O
3c
mesitylene
1.89
4.10
8.98
4.23
2.02
6.00
9.0
8.5
8.0
7.5
7.0
6.5
6.0
5.5
5.0
4.5
4.0
3.5
3.0
2.5
2.0
1.5
1.0
0.5
0.0
f1 (ppm)

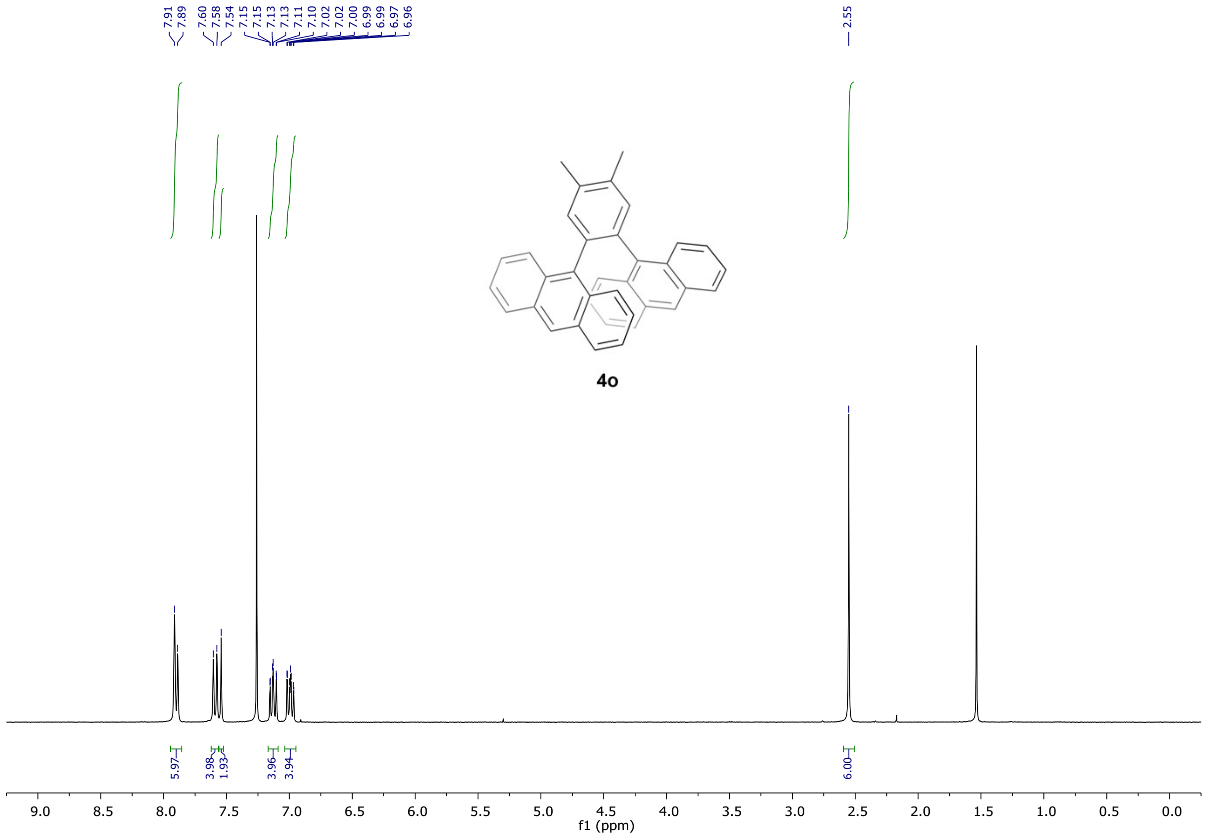
7.91
7.89
7.60
7.58
7.54
7.15
7.15
7.13
7.13
7.11
7.10
7.02
7.00
6.99
6.99
6.97
6.96
2.55
4o
5.97
3.98
1.93
3.96
3.94
6.00
9.0 8.5 8.0 7.5 7.0 6.5 6.0 5.5 5.0 4.5 4.0 3.5 3.0 2.5 2.0 1.5 1.0 0.5 0.0
f1 (ppm)

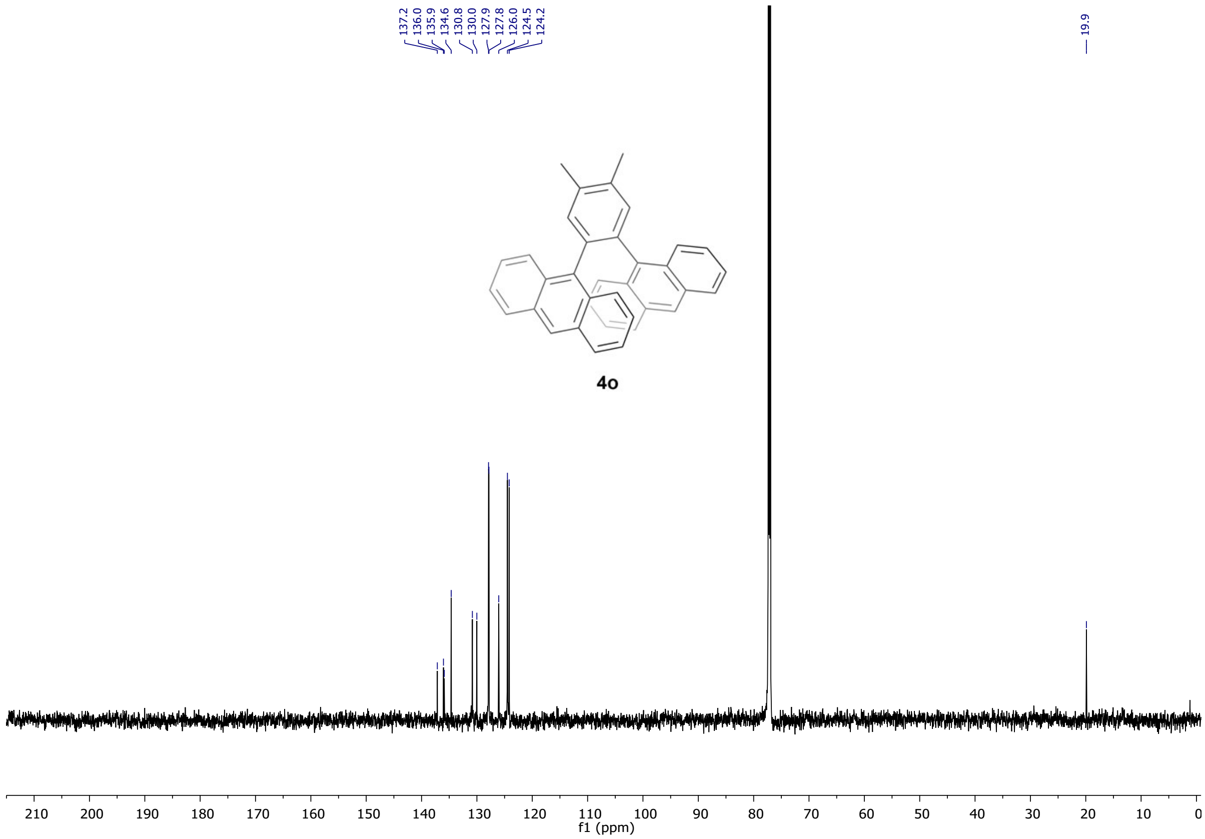
137.2
136.0
135.9
134.6
130.8
130.0
127.9
127.8
126.0
124.5
124.2
19.9
4o
210 200 190 180 170 160 150 140 130 120 110 100 90 80 70 60 50 40 30 20 10 0
f1 (ppm)

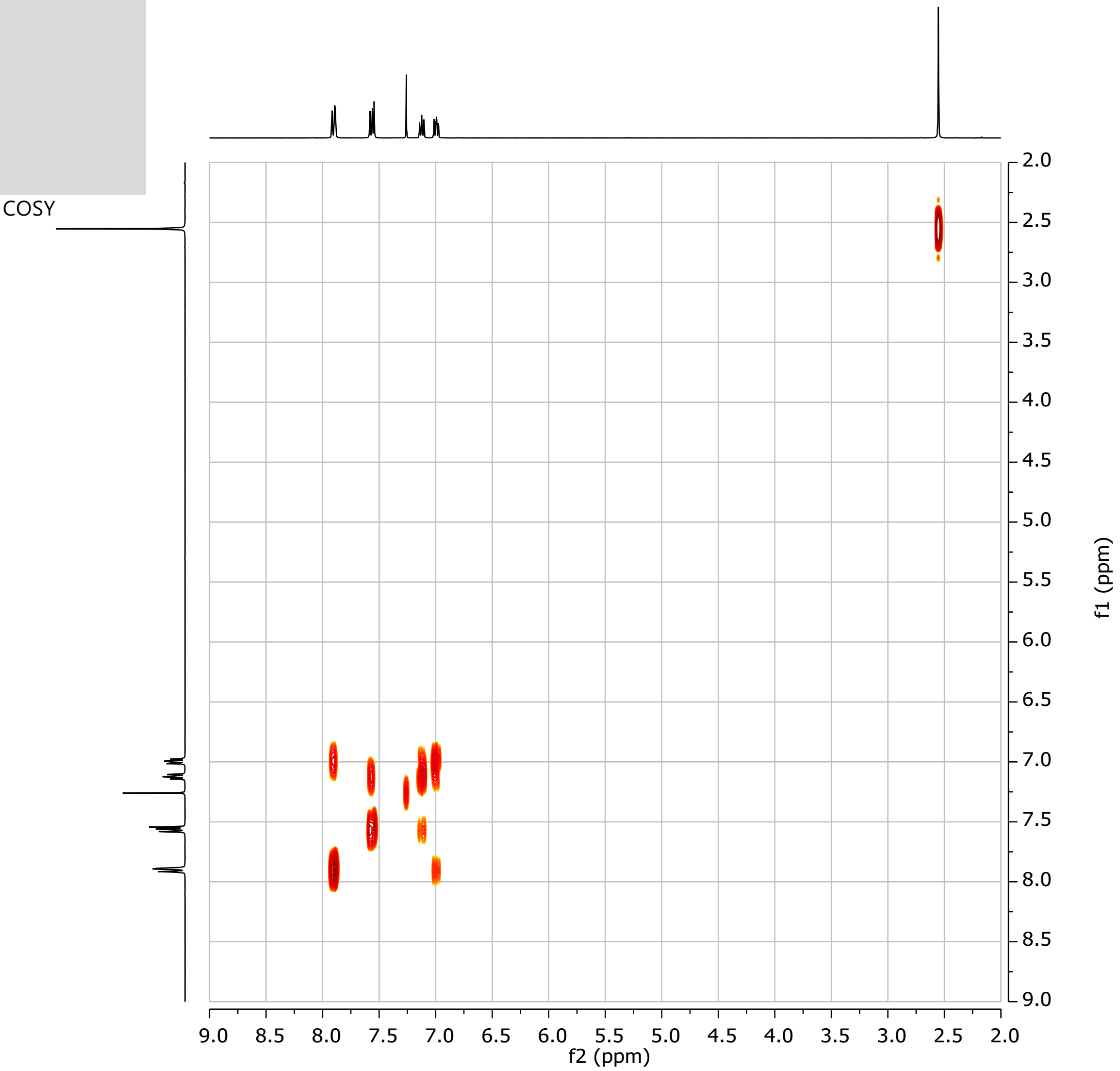
COSY
2.0
2.5
3.0
3.5
4.0
4.5
5.0
5.5
6.0
6.5
7.0
7.5
8.0
8.5
9.0
f1 (ppm)
9.0 8.5 8.0 7.5 7.0 6.5 6.0 5.5 5.0 4.5 4.0 3.5 3.0 2.5 2.0
f2 (ppm)

HSQC

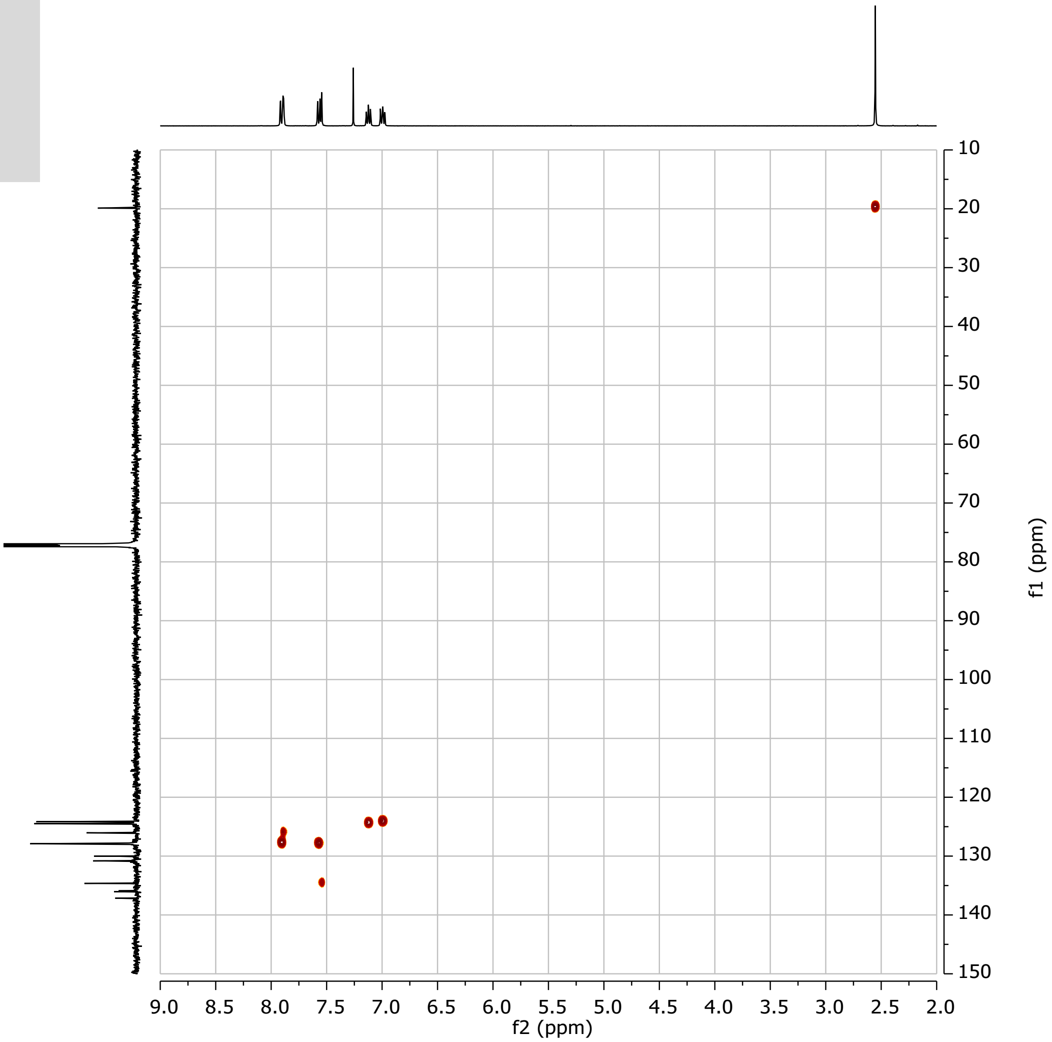

10
20
30
40
50
60
70
80
90
100
110
120
130
140
150
f1 (ppm)
9.0 8.5 8.0 7.5 7.0 6.5 6.0 5.5 5.0 4.5 4.0 3.5 3.0 2.5 2.0
f2 (ppm)

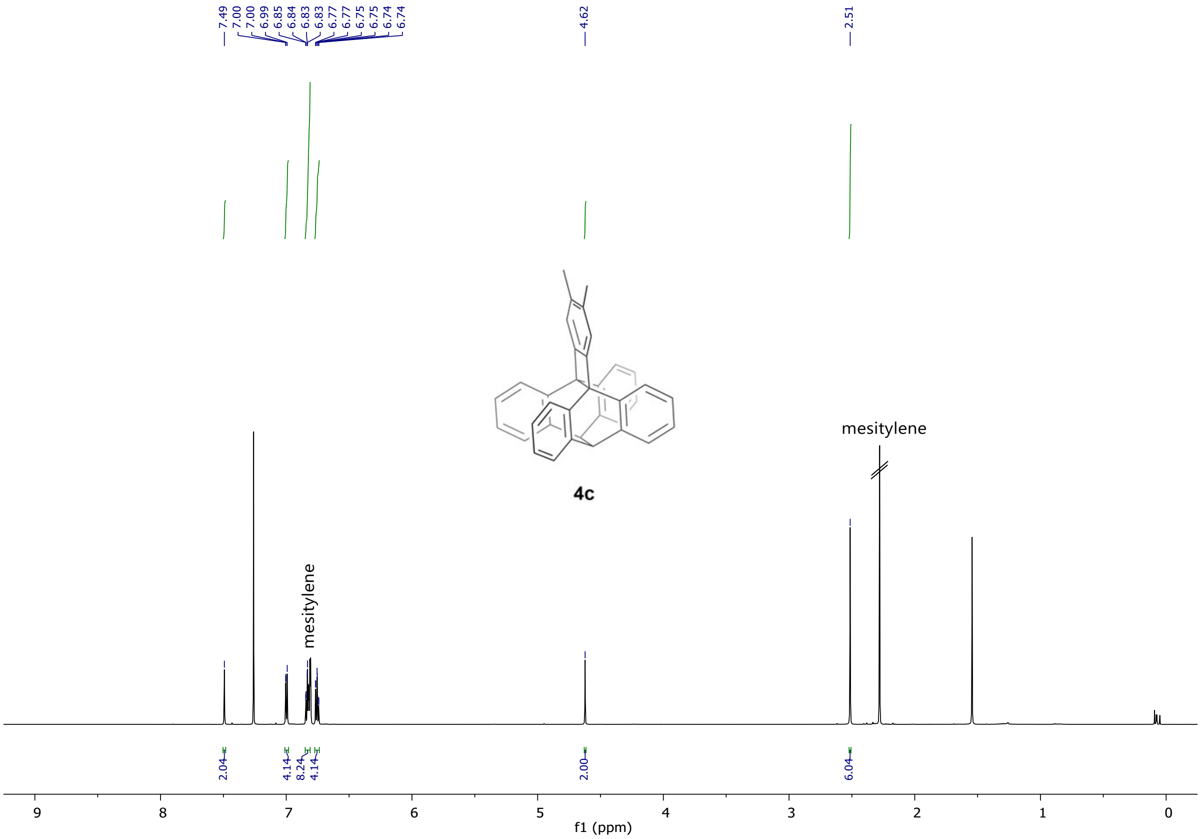
7.49
7.00
7.00
6.99
6.85
6.84
6.83
6.83
6.77
6.77
6.75
6.75
6.74
6.74
4.62
2.51
4c
mesitylene
mesitylene
2.04
4.14
8.24
4.14
2.00
6.04
f1 (ppm)

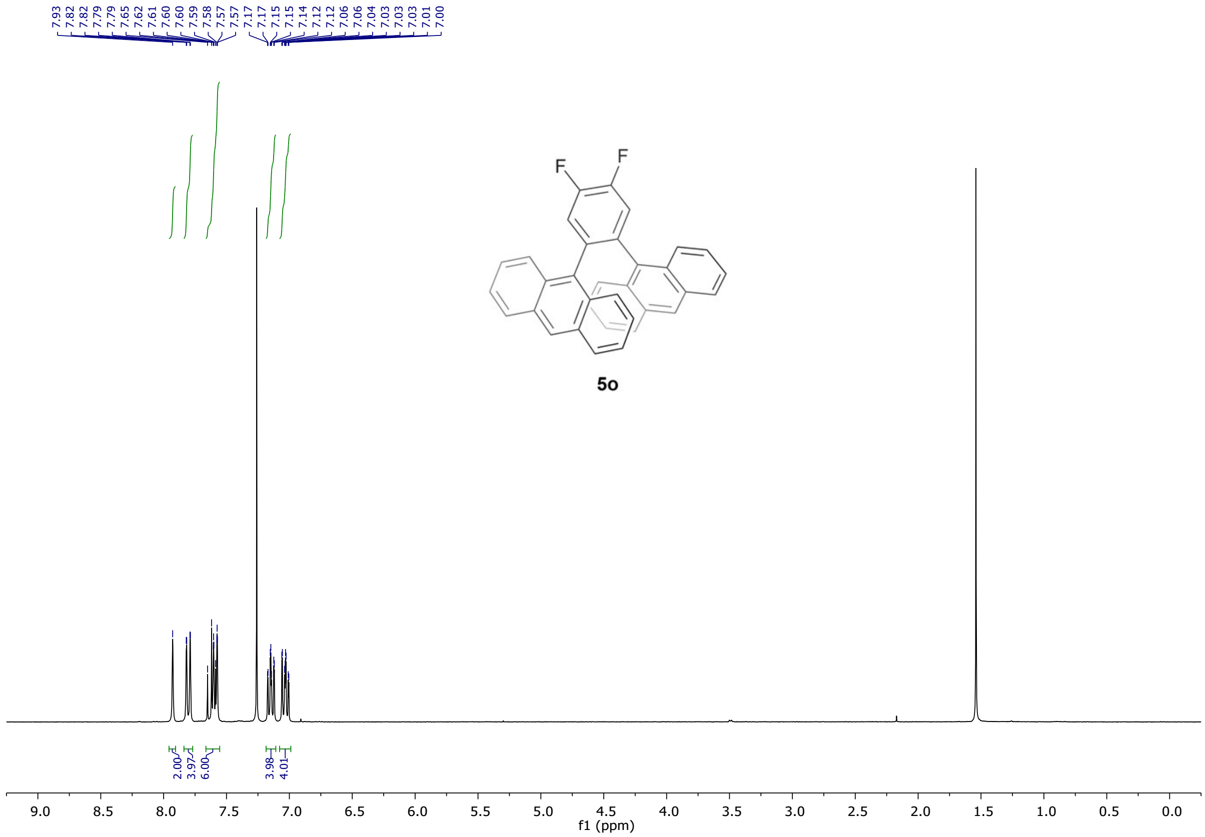
7.93 7.82 7.82 7.79 7.79 7.65 7.62 7.61 7.60 7.60 7.59 7.58 7.57 7.57 7.17 7.17 7.15 7.15 7.14 7.12 7.12 7.06 7.04 7.03 7.03 7.03 7.01 7.00
F
F
5o
2.00
3.97
6.00
3.98
4.01
9.0 8.5 8.0 7.5 7.0 6.5 6.0 5.5 5.0 4.5 4.0 3.5 3.0 2.5 2.0 1.5 1.0 0.5 0.0
f1 (ppm)

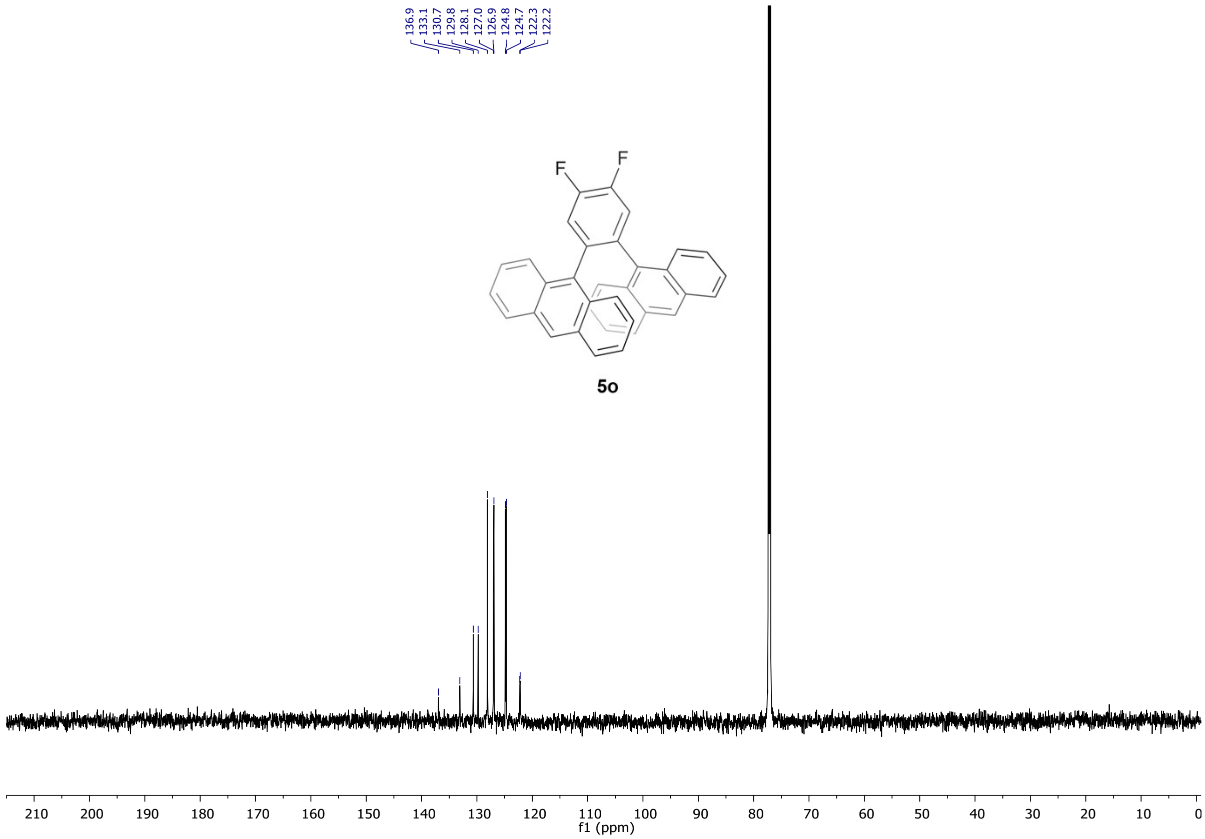
136.9 133.1 130.7 129.8 128.1 127.0 126.9 124.8 124.7 122.3 122.2
F
F
5o
210 200 190 180 170 160 150 140 130 120 110 100 90 80 70 60 50 40 30 20 10 0
f1 (ppm)

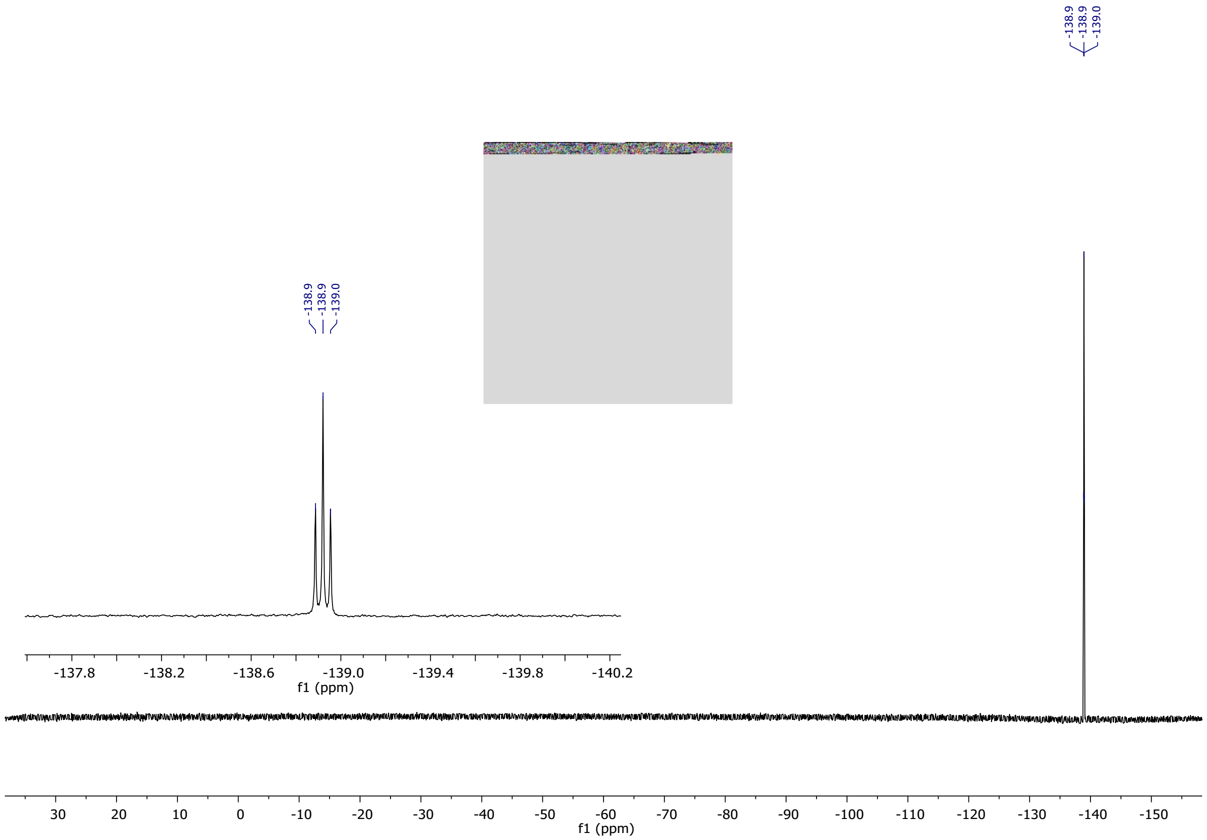

-138.9
-138.9
-139.0
-137.8
-138.2
-138.6
-139.0
-139.4
-139.8
-140.2
f1 (ppm)
30
20
10
0
-10
-20
-30
-40
-50
-60
-70
-80
-90
-100
-110
-120
-130
-140
-150
f1 (ppm)

COSY

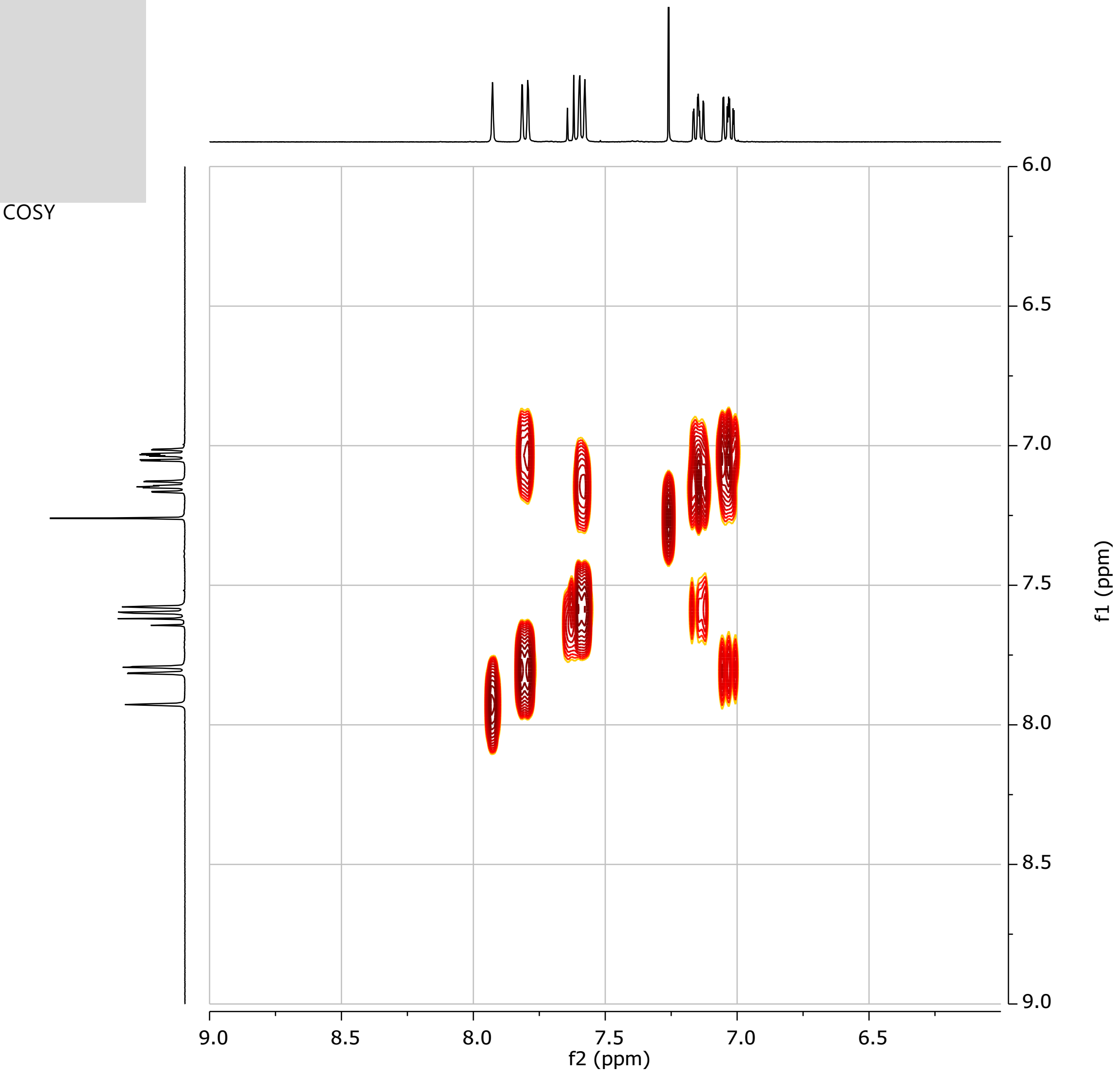

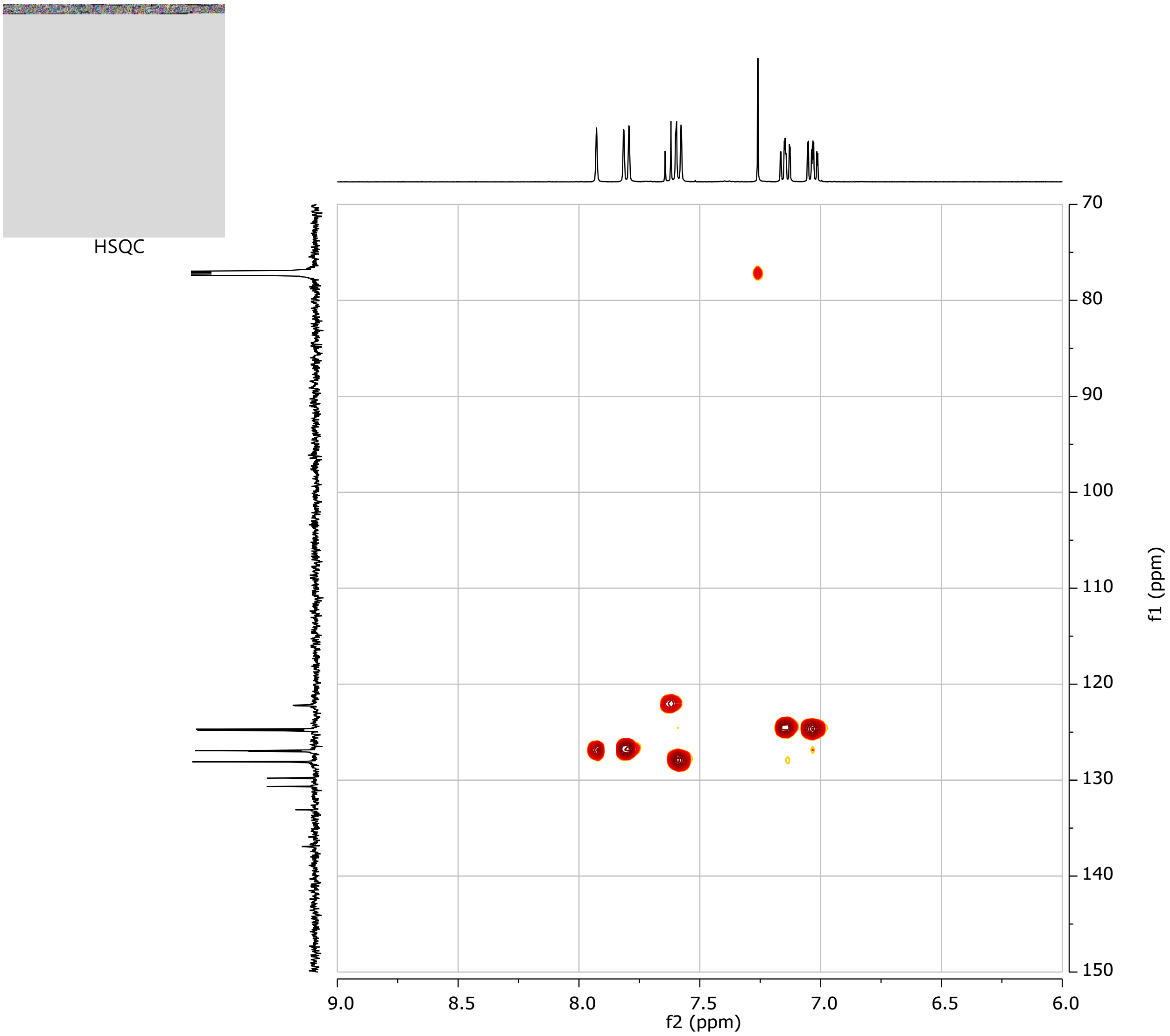
HSQC
70
80
90
100
110
120
130
140
150
f1 (ppm)
9.0
8.5
8.0
7.5
7.0
6.5
6.0
f2 (ppm)

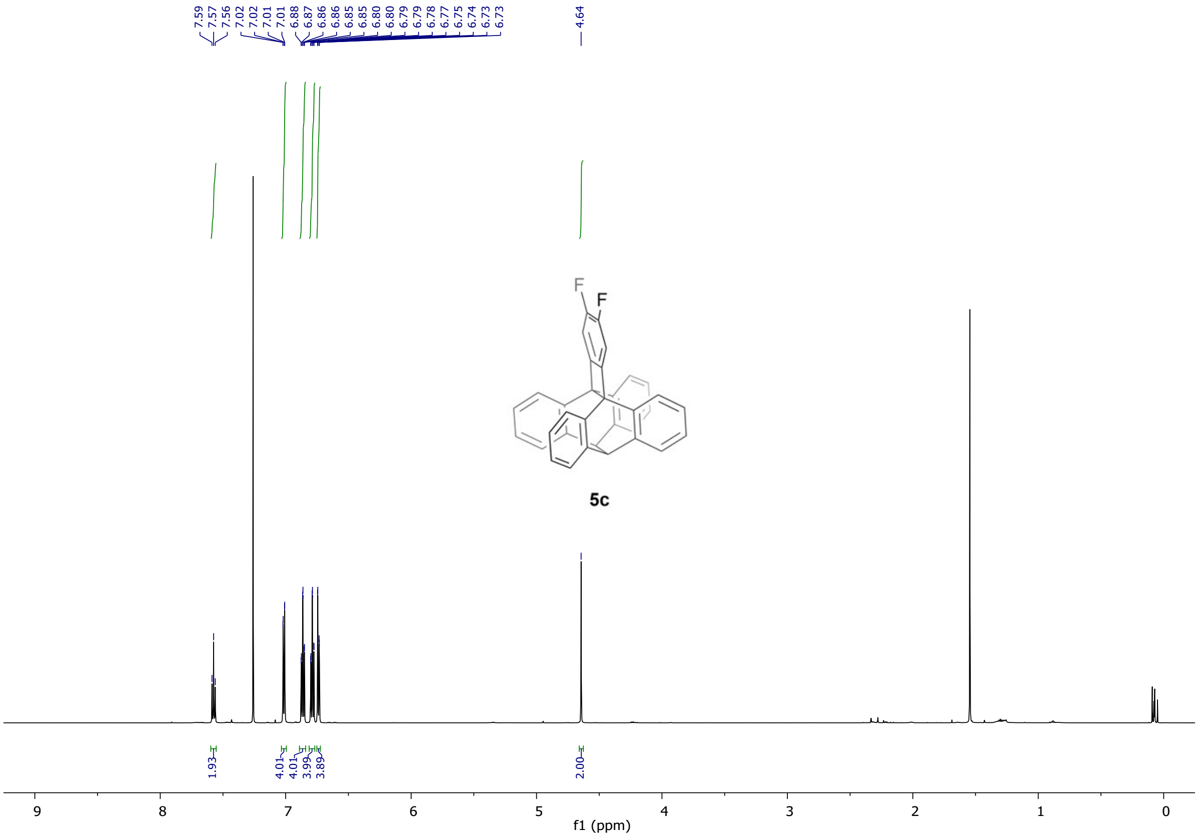

7.59
7.57
7.56
7.02
7.02
7.01
7.01
6.88
6.87
6.86
6.86
6.85
6.85
6.80
6.80
6.79
6.79
6.78
6.77
6.75
6.74
6.73
6.73
4.64
F
F
5c
1.93
4.01
4.01
3.99
3.89
2.00
9
8
7
6
5
4
3
2
1
0
f1 (ppm)

References

1. R. H. Blessing, *Acta Cryst. A*, 1995, **51**, 33–38.
2. Bruker AXS Inc., SAINT, Madison, Wisconsin, USA.
3. G. M. Sheldrick, *Acta Cryst. A*, 2015, **71**, 3–8.
4. C. G. Hatchard, C. A. Parker and E. J. Bowen, *Proc. R. Soc. Lond. A*, 1956, **235**, 518–536.
5. K. Stranius and K. Börjesson, *Sci. Rep.*, 2017, **7**, 41145.
6. J. V. Morris, M. A. Mahaney and J. R. Huber, *J. Phys. Chem.*, 1976, **80**, 969–974.
7. PerkinElmer Informatics, ChemDraw, Release 19.1, 1998, see https://perkinelmerinformatics.com/products/research/chemdraw
8. RDKit: Open-source cheminformatics, Release 2021_09_4, 2022, see https://doi.org/10.5281/zenodo.5835217
9. C. Bannwarth, S. Ehlert and S. Grimme, *J. Chem. Theory Comput.*, 2019, **15**, 1652–1671.
10. S. Grimme, A. Hansen, S. Ehlert and J.-M. Mewes, *J. Chem. Phys.*, 2021, **154**, 064103.
11. T. H. Dunning, Jr., *J. Chem. Phys.*, 1989, **90**, 1007–1023.
12. A. Najibi and L. Goerigk, *J. Chem. Theory Comput.*, 2018, **14**, 5725–5738.
13. S. Tsuzuki and T. Uchimaru, *Phys. Chem. Chem. Phys.*, 2020, **22**, 22508–22519.
14. G. Henkelman, B. P. Uberuaga and H. Jónsson, *J. Chem. Phys.*, 2000, **113**, 9901–9904.
15. F. Neese, *WIREs Comput. Mol. Sci.*, 2012, **2**, 73–78.
16. R. A. Kendall, T. H. Dunning, Jr. and R. J. Harrison, *J. Chem. Phys.*, 1992, **96**, 6796–6806.
17. A. D. Becke, *J. Chem. Phys.*, 1993, **98**, 5648–5652.
18. T. Yanai, D. P. Tew and N. C. Handy, *Chem. Phys. Lett.*, 2004, **393**, 51–57.
19. Y. Zhao and D. G. Truhlar, *Theor. Chem. Account*, 2008, **120**, 215–241.
20. J. P. Perdew, K. Burke and M. Ernzerhof, *Phys. Rev. Lett.*, 1996, **77**, 3865–3868.
21. J. P. Perdew, K. Burke and M. Ernzerhof, *Phys. Rev. Lett.*, 1997, **78**, 1396.
22. M. Ernzerhof and G. E. Scuseria, *J. Chem. Phys.*, 1999, **110**, 5029–5036.
23. C. Adamo and V. Barone, *J. Chem. Phys.*, 1999, **110**, 6158–6170.
24. V. Barone and M. Cossi, *J. Phys. Chem. A*, 1998, **102**, 1995–2001.
25. M. Cossi, N. Rega, G. Scalmani and V. Barone, *J. Comput. Chem.*, 2003, **24**, 669–681.
26. Ch. Wohlfarth, in *Landolt-Börnstein IV/17: Static Dielectric Constants of Pure Liquids and Binary Liquid Mixtures · Supplement to IV/6*, ed. M. D. Lechner, SpringerMaterials, Springer-Verlag Berlin Heidelberg © 2008, **17**, 456.
27. W. M. Haynes (Ed.), *CRC Handbook of Chemistry and Physics*, 73th ed., CRC Press, Boca Raton, 1992.
